# Proton transfers in the Strecker reaction revealed by DFT calculations

**DOI:** 10.3762/bjoc.10.184

**Published:** 2014-08-01

**Authors:** Shinichi Yamabe, Guixiang Zeng, Wei Guan, Shigeyoshi Sakaki

**Affiliations:** 1 Fukui Institute for Fundamental Chemistry, Kyoto University, Takano-Nishihiraki-cho 34-4, Sakyo-ku, Kyoto 606-8103, JAPAN. Phone: +81-075-711-7907

**Keywords:** amide intermediate, DFT calculations, hydrogen bonds, Strecker reaction, transition state

## Abstract

The Strecker reaction of acetaldehyde, NH_3_, and HCN to afford alanine was studied by DFT calculations for the first time, which involves two reaction stages. In the first reaction stage, the aminonitrile was formed. The rate-determining step is the deprotonation of the NH_3_^+^ group in MeCH(OH)-NH_3_^+^ to form 1-aminoethanol, which occurs with an activation energy barrier (Δ*E*^≠^) of 9.6 kcal/mol. The stereochemistry (*R* or *S*) of the aminonitrile product is determined at the NH_3_ addition step to the carbonyl carbon of the aldehyde. While the addition of CN^−^ to the carbon atom of the protonated imine **7** appears to scramble the stereochemistry, the water cluster above the imine plane reinforces the CN^−^ to attack the imine group below the plane. The enforcement hinders the scrambling. In the second stage, the aminonitrile transforms to alanine, where an amide Me-CH(NH_2_)-C(=O)-NH_2_ is the key intermediate. The rate-determining step is the hydrolysis of the cyano group of N(amino)-protonated aminonitrile which occurs with an Δ*E*^≠^ value of 34.7 kcal/mol. In the Strecker reaction, the proton transfer along the hydrogen bonds plays a crucial role.

## Introduction

**Scheme 1:**
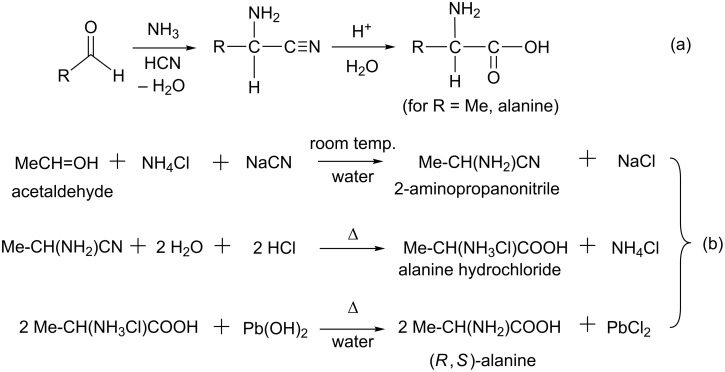
The general form of the Strecker reaction. The reaction (b) is taken from [2].

In 1850, Adolph Strecker reported a reaction that affords alanine from acetaldehyde, ammonia and hydrogen cyanide [1]. The original form of Strecker amino acid synthesis is shown in Scheme 1(a). In this reaction, the aldehyde reacts with hydrogen cyanide to form an aminonitrile, which undergoes hydrolysis to afford alanine in the acidic solution. The traditional Strecker reaction gave racemic α-aminonitriles (mixtures of equal amounts of *R* and *S* forms), where an imine RCH=NH was considered to be the key intermediate [2]. Three typical reactions are presented in Scheme 1(b) [3].

**Scheme 2:**
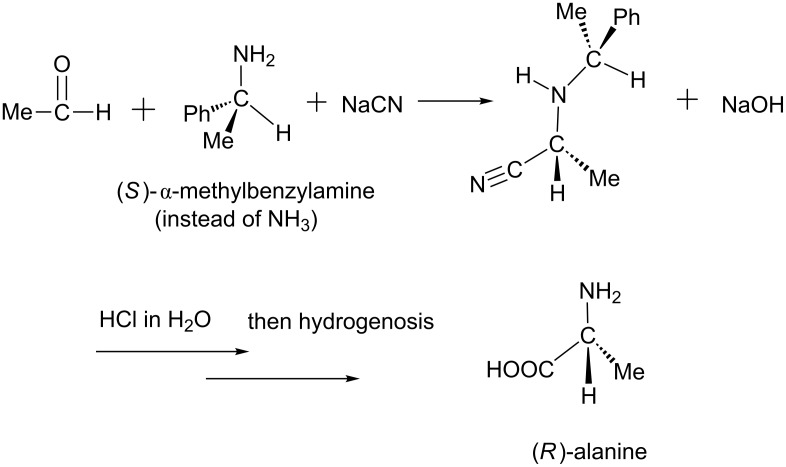
The first asymmetric Strecker reaction [4].

**Scheme 3:**
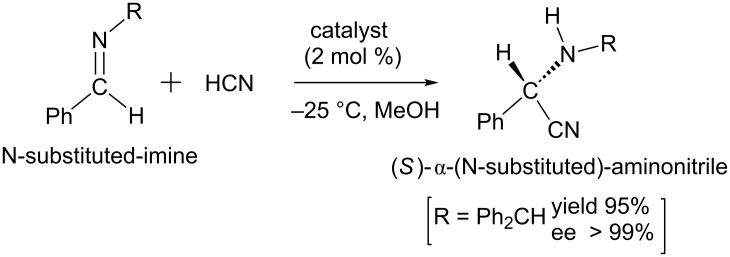
The first asymmetric synthesis of α-aminonitirles via a chiral catalyst [5].

In 1963, Harada reported the first asymmetric Strecker reaction, in which an (S)-α-phenylethylamine was employed as the chiral auxiliary [4]. In this reaction, he obtained a chiral alanine with 95% optically activity; see Scheme 2. In 1996, Lipton et al. succeeded in a series of asymmetric Strecker reactions by employing a chiral catalyst, a cyclic dipeptide [5]. In these reactions, N-substituted imines react with HCN to yield (S)-α-aminonitriles with remarkably high enantiomeric excess (ee). One example is shown in Scheme 3.

However, when benzaldehyde and NH_3_ instead of the N-substituted imine were employed as the substrates, the reaction afforded an initial product Ph-CH(NH_2_)-CN of configurational instability[5]. In the following, Sigman and Jacobsen used a parallel combinatorial library synthesis for the discovery and optimization of a chiral catalyst for the reaction of imines and HCN [6]. From then on, various catalytic asymmetric Strecker reactions have been reported to gain high enantioselectivity of the hydrocyanation reaction of imines [7–12]. However, the origin of the enantioselectivity in the asymmetric Strecker reactions has not been clarified.

To our knowledge, the elementary processes of the whole Strecker reaction have not been elucidated. As shown in Scheme 1, the Strecker reaction includes two reaction stages. The first reaction stage is the condensation of aldehydes with ammonia and hydrogen cyanide leading to α-aminonitriles . The second reaction stage is the hydrolysis of the nitrile group. In these reactions, K^+^ (or Na^+^) and Cl^−^ ions are not involved, as shown in Scheme 1(a). Therefore, it is suitable to the theoretical investigation of the reaction mechanism, because the effect of counter ions does not need to be considered.

Actually, several theoretical studies were reported of the last step of the first reaction stage of the Strecker reaction [10–16], i.e. the hydrocyanation of imines (or protonated imines + CN^−^) to aminonitriles. In those works, how the nucleophile CN^−^ is generated has not been examined. Because HCN is a very weak acid with a dissociation constant of *K*_a_ = 1.3 × 10^−9^ mol/L (in water, 18 °C), direct dissociation reaction HCN → H^+^ + CN^−^ is difficult to occur.

In the second reaction stage, i.e., the acid-catalyzed hydrolysis of the cyano group, protonation of the group appears to cause the addition of OH_2_ to the cyano carbon:

R-CH(NH_2_)-CN + H^+^ → R-CH(NH_2_)-CNH^+^

R-CH(NH_2_)-CNH^+^ + OH_2_ → R-CH(NH_2_)-C(OH)=NH + H^+^

However, the proton affinity (PA) of the nitrile is much smaller than that of the amino group, for example, the PAs of the cyano and amino groups of 2-amino-propanonitrile (Me-CH(NH_2_)-CN) are 190.7 and 199.6 kcal/mol, respectively. Thus, in the acidic solution (2H_2_O + 2HCl), Me-CH(NH_3_^+^)-CN should be afforded; see Scheme 1(b). The reaction mechanism of this hydrolysis is also unclear.

To address the above issues, we performed DFT calculations of the Strecker reaction shown in Scheme 1(a). Here, ten specific water molecules were considered.

## Methods of calculation

Geometry optimizations were performed by density functional theory (DFT) with the B3LYP [17–18] functional. The basis set 6-311+G(d,p) was employed for all the atoms in the calculations. The solution (water) effect was considered by the Polarizable Continuum Model (PCM) [19–21]. Vibrational analyses were carried out to make sure whether a stationary point is an equilibrium structure or a transition state (TS). From TSs, reaction paths were traced by the intrinsic reaction coordinate (IRC) method [22–23] to obtain the energy-minimum geometries. All the calculations were carried out using the *GAUSSIAN* 09 [24] program package. Throughout this paper, the discussion was presented based on the potential energy changes with zero-point vibrational energy (ZPE) correction unless otherwise noted.

## Results and Discussion

### Formation reaction of aminonitrile (the first stage)

**Scheme 4:**
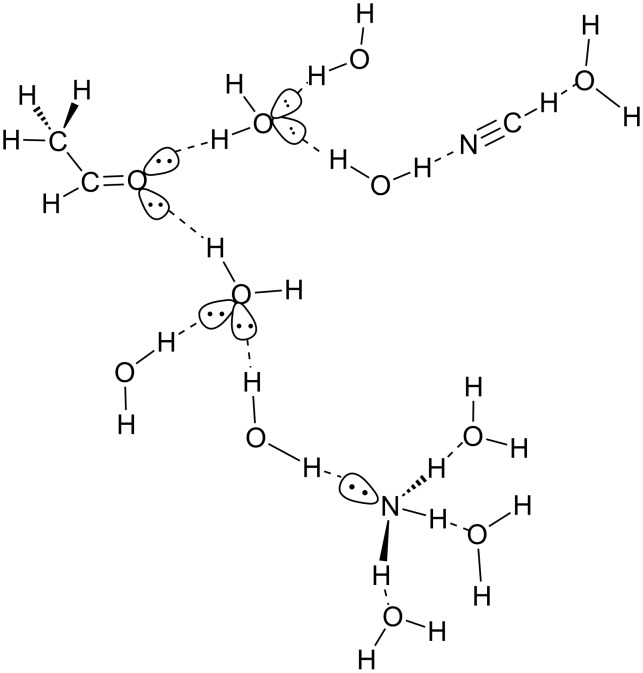
A reaction model composed of Me-CH=O, HCN, NH_3_ and (H_2_O)_10_ for geometry optimizations to trace elementary processes. Broken lines stand for hydrogen bonds.

The reaction model is shown in Scheme 4. In the model, lone-pair electrons of the oxygen and nitrogen atoms participate in hydrogen bonds. In calculating each TS, ten water molecules were placed so that the large hydrogen-bond stabilization is gained. After geometries of TSs were determined, those of energy minima were obtained by IRC and the subsequent optimizations. By the use of the similar water cluster models, TS geometries and activation energies (*E*_a_'s) in the base promoted ester hydrolyses were calculated [25]. In Ph-COOE_t_ + OH^−^(H_2_O)*_n_*→Ph-COO^−^ + HO-E_t_ + (H_2_O)*_n_*, *E*_a_ = +14.7 kcal/mol (*n* = 5), +16.3 (*n* = 8), +16.3 (*n* = 12) and +15.6 (*n* = 16) were obtained, where the experimental *E*_a_ is + 14.6 kcal/mol. Also, in the Bamberger rearrangement Ph-NH(OH) + (H_3_O^+^)_2_(H_2_O)_13_ → para-HO-C_6_H_4_-NH_3_^+^ + H_3_O^+^(H_2_O)_14_ [26], *E*_a_ = +26.3 kcal/mol was calculated, where the experimental *E*_a_ is +24.8 kcal/mol.

**Scheme 5:**
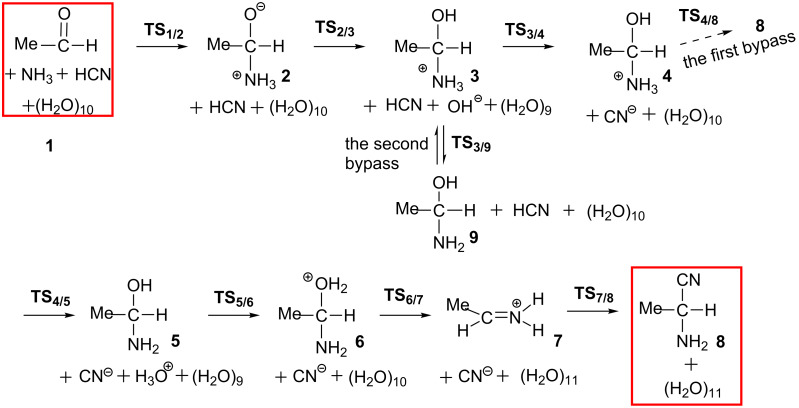
Possible pathways for the formation of aminonitrile from acetaldehyde.

**Figure 1:**
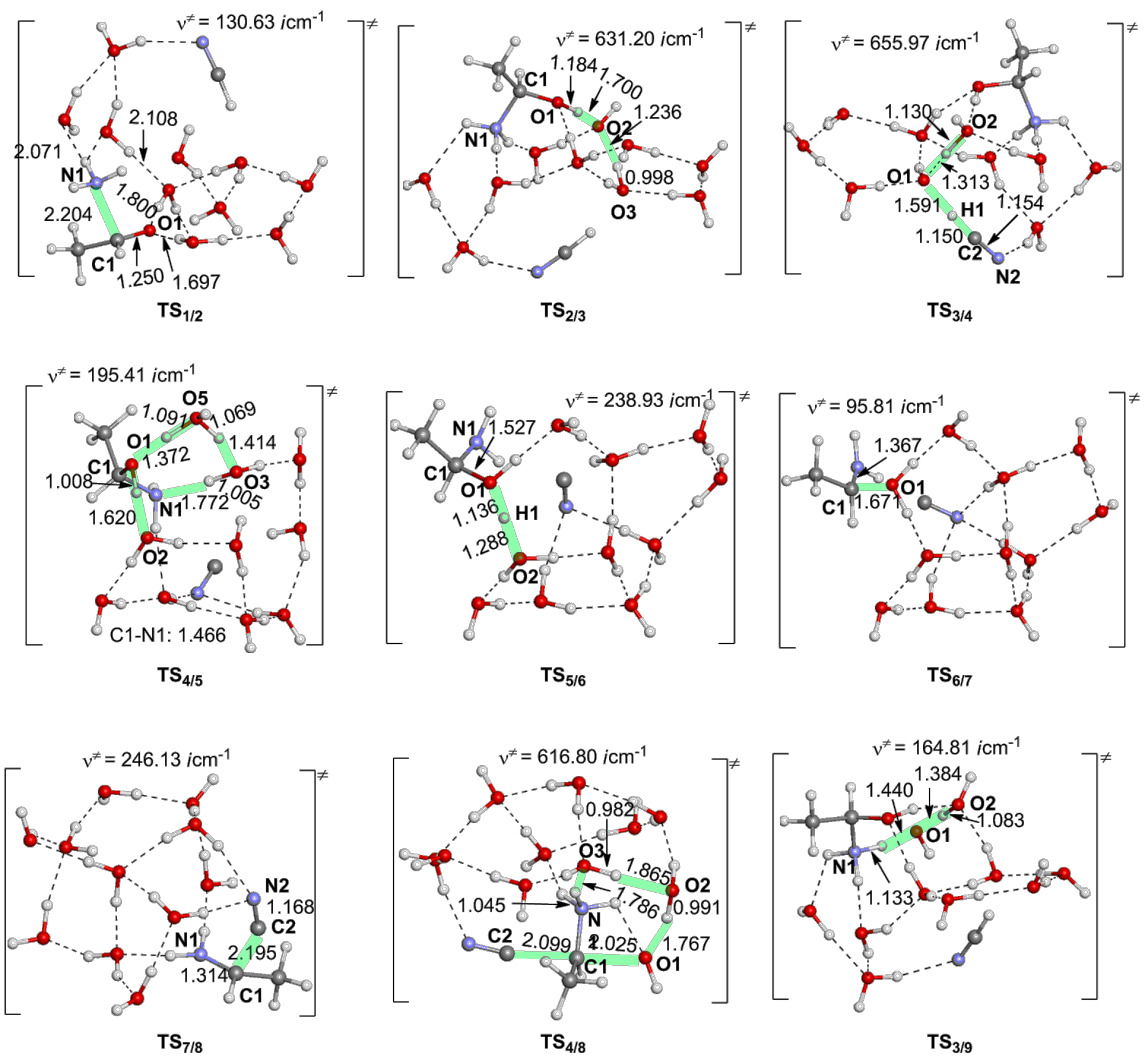
Geometries of transition states along the reaction from acetaldehyde (**1**) to the aminonitrile **8**. Distances are in Å. **TS_1/2_** means, for instance, a transition state for the step **1** → **2**.

Our proposed reaction pathways are shown in Scheme 5. Geometries of TSs are shown in Figure 1 and those of precursor **1**, intermediates and product **8** are provided in Supporting Information File 1 Figure S1.

**Figure 2:**
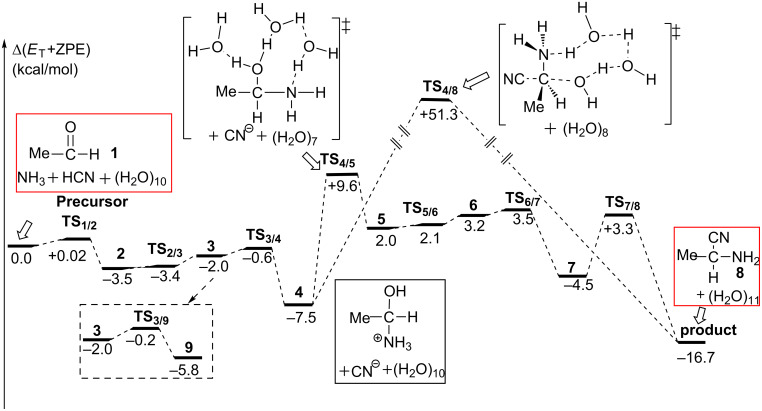
Energy changes along elementary processes from acetaldehyde to aminonitrile. Bold numbers are defined in Scheme 5.

As shown in Figure S1, Supporting Information File 1, MeCH=O, NH_3_ and HCN are separated by the H_2_O cluster in the precursor complex **1**. The reaction begins with the addition of NH_3_ to the carbonyl carbon of acetaldehyde to form a Mulliken charge-transfer complex **2**. This complex was firstly proposed here. In **2**, the C–O bond is elongated to1.345 Å, which has an alkoxide character and the complex is not stable in the gas phase. However, it is more stable than the precursor complex by 3.5 kcal/mol when ten specific water molecules are considered; see Figure 2. This result indicates that the consideration of water molecules in the reaction is necessary to describe the step **1** → **2**. Then, the alkoxide oxygen atom captures a proton from a surrounding water molecule to form Me(H)C(OH)-NH_3_^+^ and a remaining OH^−^ anion in the surrounding. They form an ion pair **3** [Me(H)C(OH)-NH_3_^+^ and OH^−^]. Next, the hydroxide ion catches a proton from HCN through the transition state **TS_3/4_** to afford a more stable ion-pair intermediate **4** [Me(H)C(OH)-NH_3_^+^ and CN^−^]. This step is exothermic by 5.5 kcal/mol with a small activation energy barrier (Δ*E*^≠^) of 1.4 kcal/mol. Starting from **4**, a proton of the NH_3_^+^ group migrates to one water molecule to form **5** [Me(H)C(OH)-NH_2_(H_3_O^+^) and CN^−^] via a transition state **TS_4/5_**. After that, the proton migrates from H_3_O^+^ to the hydroxy group of Me(H)C(OH)-NH_2_ via **TS_5/6_** to yield Me(H)C(OH_2_^+^)-NH_2_ **6**. From **6**, H_2_O is easily eliminated through **TS_6/7_** to afford the protonated imine **7** with a Δ*E*^≠^ value of 0.3 kcal/mol. At last, CN^−^ nucleophilically attacks the carbon atom of MeCH=NH_2_^+^ through **TS_7/8_** to afford a 2-aminopropanonitrile **8**.

**Scheme 6:**
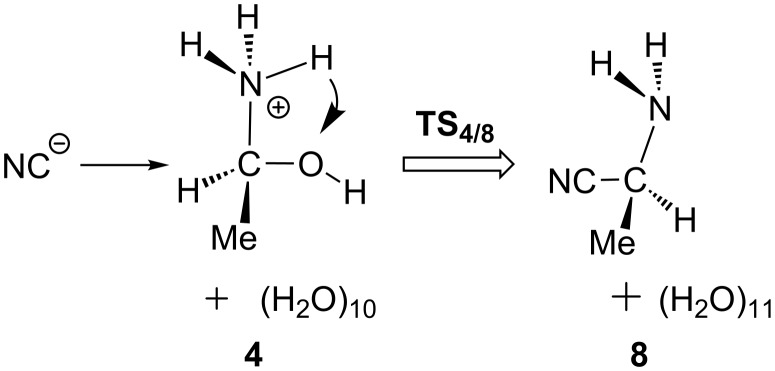
A short-cut path by the nucleophilic displacement and the concomitant proton transfer. “The first bypass” in Scheme 5.

Starting from **4**, a concerted S_N_2-type pathway was also examined, which directly leads to the nitrile compound **8**; see Scheme 6. In this pathway, the proton is transferred from the NH_3_^+^ group to the hydroxy group via a two-water-molecule bridge. At the same time, the H_2_O elimination and the approach of CN^−^ to **4** concomitantly take place with a Walden inversion. The transition state **TS_4/8_** was successfully located; see Figure 1. However, this pathway needs a large energy barrier of 58.8 = [+51.3 − (−7.5)] kcal/mol, indicating that it is difficult to occur.

Starting from the ion-pair intermediate **3**, we also investigated the possibility that the hydroxide ion captures a proton from the NH_3_^+^ group to form **9**, Me-C(H)(OH)-NH_3_^+^ + OH^−^ → Me-C(H)(OH)-NH_2_ + OH_2_. This reaction step occurs through a transition state **TS_3/9_** with a Δ*E*^≠^ value of 1.9 kcal/mol, which is comparable to that (1.4 kcal/mol) of the proton transfer step from HCN to OH^−^. However, **9** is less stable than **4** by 1.8 kcal/mol, indicating that the OH^−^ prefers to capture a proton from HCN rather than from the NH_3_^+^ group.

As shown in Figure 2, the rate-determining step of this reaction stage is the proton migration from the NH_3_^+^ group to the water cluster (from **4** to **5**), where the energy barrier is 17.1= [+9.6 − (−7.5)] kcal/mol. Other proton transfer steps facilely occur. These results are in consistent with the room-temperature experimental condition in Scheme 1. For the rate-determining step, we also checked an extended model "**TS_4/5_–ext**", where ten water molecules are added (the molecular formula, C_3_H_48_N_2_O_21_). The geometry of **TS_4/5_–ext** is shown in Supporting Information File 1 Figure S2. The geometrical parameters of the proton-transfer region of **TS_4/5_–ext** are similar to that of **TS_4/5_**. Also, the energy difference between **TS_4/5_–ext** and **4–ext** (17.3 kcal/mol) is very close to that (17.1 kcal/mol) between **TS_4/5_** and **4**.

**Scheme 7:**
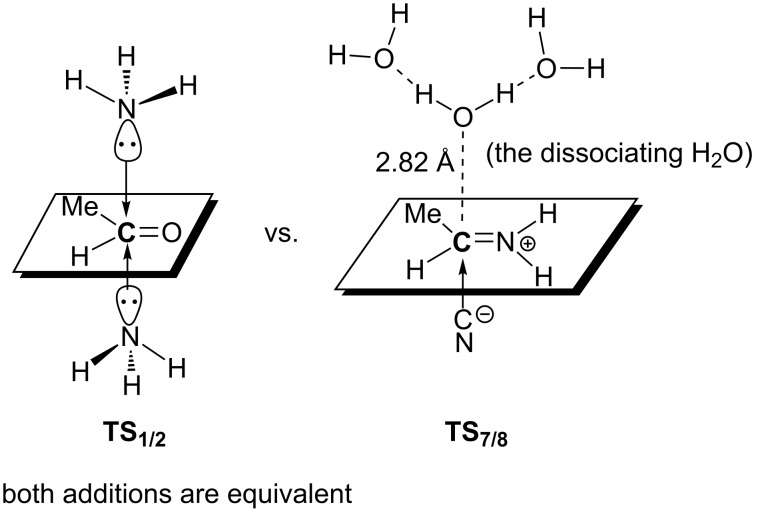
A contrast of the nucleophilic addition.

**Figure 3:**
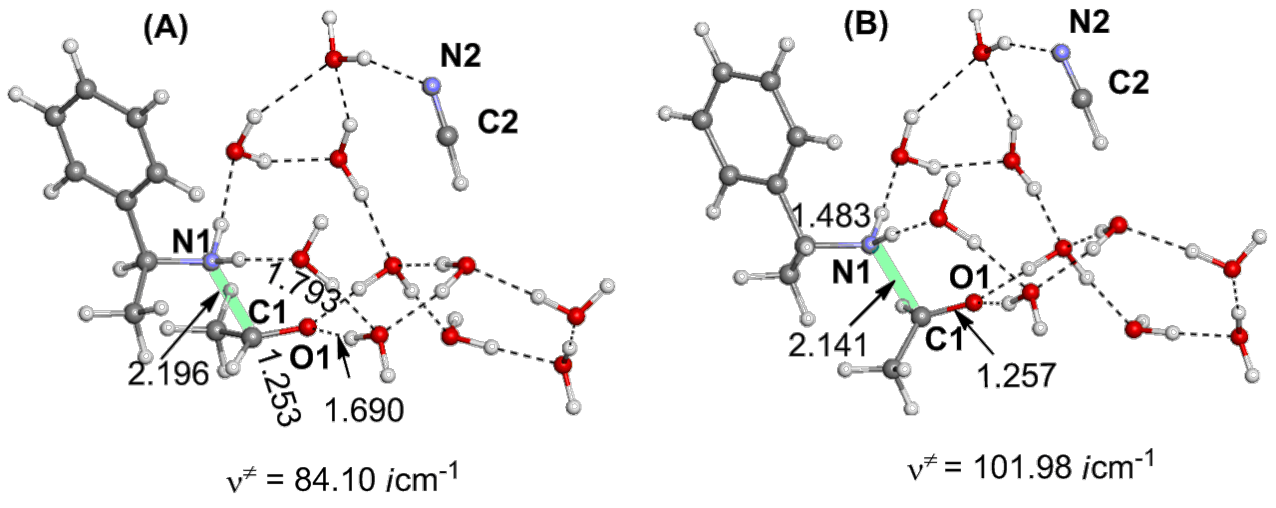
Two transition states (A and B) of the nucleophilic addition of (S)-α-phenylethylamine to acetaldehyde. (H_2_O)_10_ is also included, and the molecular formula of the reaction system is C_11_H_36_N_2_O_11_.

As shown in Figure 1, the product, aminonitrile **8**, is in an *S* form. However, racemic α-aminonitriles are obtained experimentally. This stereochemical scrambling is explicable on the basis of the computational results, as follows. In **TS_1/2_**, the NH_3_ molecule may add to MeCH=O from both upper and lower directions equivalently, which leads to the racemic products. However, in **TS_7/8_**, the nucleophile CN^−^ is obligated to attack MeC(H)=NH_2_^+^ at the plane opposite to the OH_2_ dissociating side (see Scheme 7). The addition model of **TS_1/2_** was examined by the use of the amine in Scheme 2. The activation energy of the less crowded **TS_1/2_–A** is 1.8 kcal/mol smaller than the more crowded **TS_1/2_–B**; see Figure 3. This calculation result is consistent with Harada's work that a chiral product [4] was obtained in the Strecker reaction.

### Hydrolysis of amino nitrile to amino acid (the second stage)

**Scheme 8:**
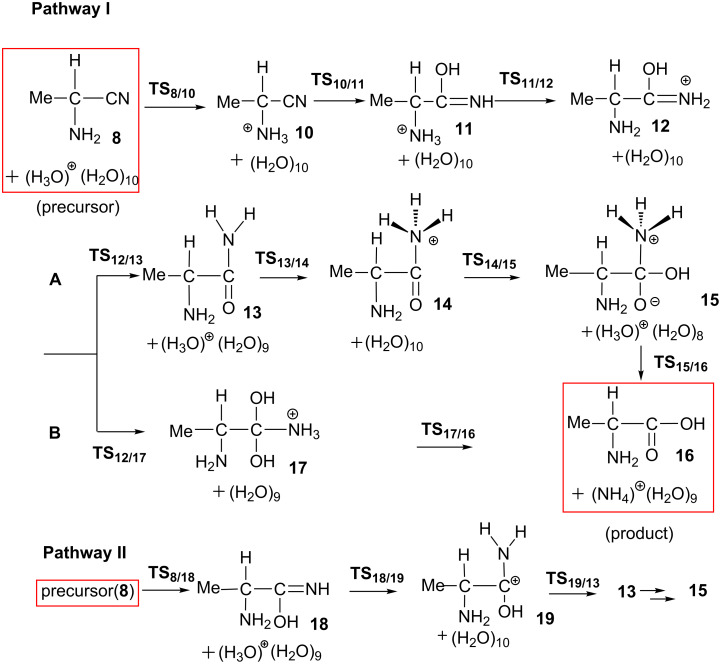
Elementary processes of the acid-catalyzed hydrolysis of 2-amino-propanonitrile.

In the acidic hydrolysis of amino nitrile, we take 2-amino-propanonitrile +H_3_O^+^(H_2_O)_10_ (**8**) as a precursor complex; see Figure S3 in Supporting Information File 1 for the geometry of **8**. Our proposed pathways are shown in Scheme 8.

**Figure 4:**
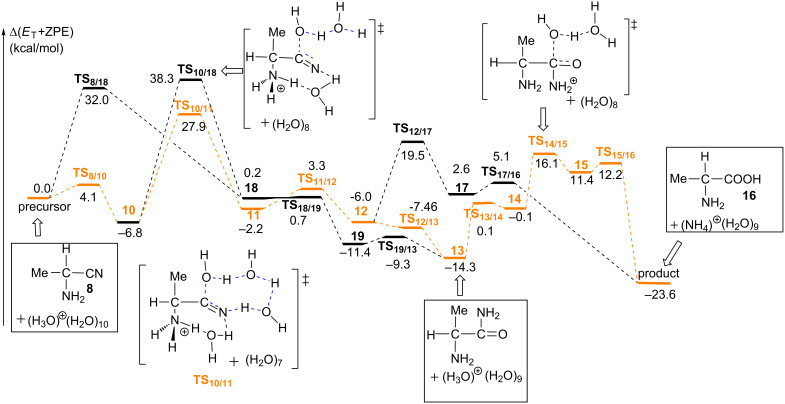
Energy changes along elementary processes from 2-amino nitrile **8** to 2-amino acid **16**. Brown-color lines stand for the most favorable route.

There are two competitive pathways from the precursor complex: One (**I**) is the protonation of the amino group to form a N(on amino)-protonated aminonitrile **10**. This step occurs through **TS_8/10_** with the Δ*E*^≠^ and Δ*E* values of 4.1 and −6.8 kcal/mol, respectively; see Figure 4.

**Figure 5:**
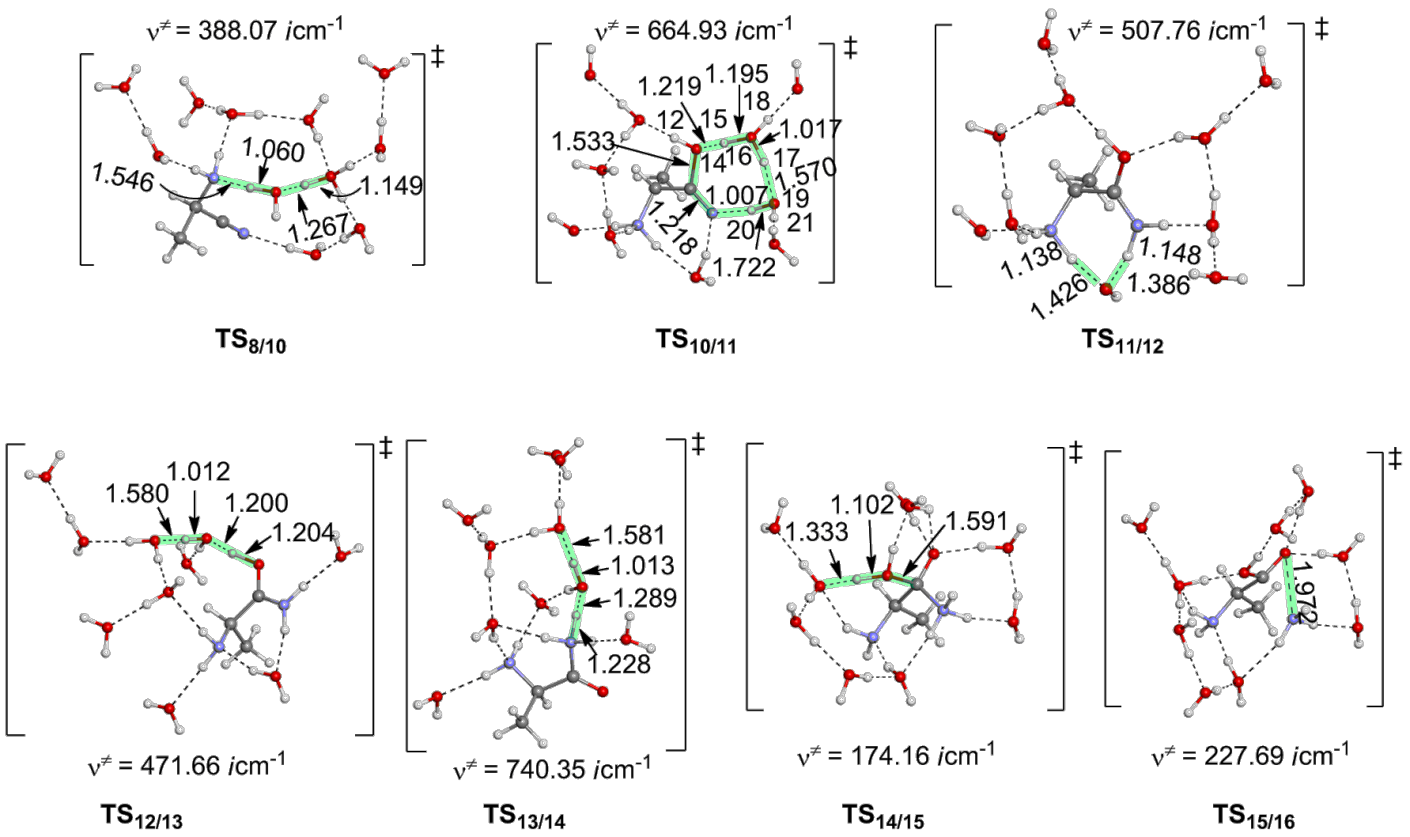
Geometries of transition states along the most favorable route from 2-aminonitrile **8** to 2-amino acid **16**.

The other pathway (**II**) is the hydrolysis of the C≡N group to form a compound **18** through **TS_8/18_**, where the OH group is added to the carbon atom and the hydrogen atom attachs to the nitrogen atom. This step needs a considerably large energy barrier of 32.0 kcal/mol with an endothermicity of 0.2 kcal/mol. Obviously, the protonation of the amino group (**I**) is much more favorable than the hydrolysis of the cyano group (**II**). As a result, compound **10** is the starting point for the following reactions. From **10**, a water trimer reacts with the cyano group through **TS_10/11_** to afford the N(on amino)-protonated 2-amino-1-hydroxypropanimine **11**. At **TS_10/11_**, the hydroxy group adds to the carbon atom of the cyano carbon nucleophilically. Simultaneously, the proton migrates to the nitrogen atom through a two-water-bridge; see Figure 5 for the geometry of **TS_10/11_**. The C≡N group in **10** convers to a C(OH)=NH group in **11**. This reaction step is endothermic by 4.6 = [−2.2 − (−6.8)] kcal/mol with a large Δ*E*^≠^ value of 34.7 = [+27.9 − (−6.8)] kcal/mol. Although this Δ*E*^≠^ value is apparently larger than that of **TS_8/18_**, **TS_10/11_** lies lower than **TS_8/18_** by 4.1 kcal/mol when taking the energy of the precursor complex as a reference; see Figure 4. We examined the role of the NH_3_^+^ group in the reaction by investigating the hydrolysis of the cyano group of a methyl-substituted model **10**(Me). In this model, we replaced the NH_3_^+^ group in **10** with a methyl group. The Δ*E*^≠^ value of the hydrolysis step increases to 34.6 kcal/mol. It indicates that the NH_3_^+^ group enhances the electrophilicity of the cyano carbon, which is favorable for the OH_2_ addition. The TS geometry of the methyl substituted model, **TS_10/11_**(Me), is shown in Supporting Information File 1 Figure S6.

In the following, a proton on the NH_3_^+^ group in **11** is transferred to the imine nitrogen to afford **12**. This proton transfer step is facilitated by a water molecule bridge. The Δ*E*^≠^ and Δ*E* values are 5.5 = [+3.3 − (−2.2)] and −3.8 = [−6.0 −(2.2)] kcal/mol, respectively. Starting from **12**, there are two possible pathways, paths **A** and **B**, to form the final alanine product; see Scheme 8. In path **A**, the deprotonation of the amino group occurs to produce an amide intermediate **13** with an exothermicity of −8.3 = [−14.3 − (−6.0)] kcal/mol; see Figure 4. In the following, the protonation of the amide nitrogen atom occurs to produce a cationic species MeC(NH_2_)H-C(=O)-NH_3_^+ ^**14**. The amide carbon in **14** is subject to the OH_2_ addition to afford a zwitterion compound MeC(NH_2_)H-C(OH)(O^−^)-NH_3_^+^ **15**. The Δ*E*^≠^ and Δ*E* values of the H_2_O addition step relative to the energy of **13** are 30.5 and 25.8 kcal/mol, respectively. After that, the NH_3_ moiety is ready to dissociate from **15** to produce the product (*R*)-alanine **16** with a Δ*E*^≠^ value of 0.7 kcal/mol of **TS_15/16_**. In path **B**, the second H_2_O molecule is added to the C=N double bond in **12** to form an intermediate **17** through **TS_12/17_**. The Δ*E*^≠^ and Δ*E* values of this step are 25.5 and 8.6 kcal/mol, respectively. However, **TS_12/17_** lies higher than **TS_14/15_** by 3.1 kcal/mol and **17** in path **B** is much more unstable than the intermediate **13** in path **A** by 17.0 kcal/mol. These energy differences suggest that the deprotonation of the amino group occurs more favorably. Then, from **17** the elimination of the NH_4_^+^ group takes place to afford the alanine **16** with a Δ*E*^≠^ value of 2.5 kcal/mol. According to the above discussion, the path-**B** is less favorable than the path **A**.

The most favorable pathway for the second stage of the Strecker reaction was shown in brown color in Figure 4. The rate-determining step is the OH_2_ addition to the cyano group (**TS_10/11_**) with the activation energy of 27.9 kcal/mol. The competitive **TS_8/18_** has an energy barrier of 32.0 kcal/mol. Geometries along the unfavorable routes pathway **II** and pathway **IB**, are shown in Figures S4 and S5, Supporting Information File 1, respectively. The relative stability of these two transition states were checked with extended models **TS_10/11_**-**ext** and **TS_8/18_**-**ext**, which have a molecular formula of C_3_H_49_N_2_O_21_^+^. Their geometries are shown in Figure S7. **TS_8/18_**-**ext** lies higher than **TS_10/11_**-**ext** by 3.4 kcal/mol, which is consistent with the small model system.

## Conclusion

**Scheme 9:**
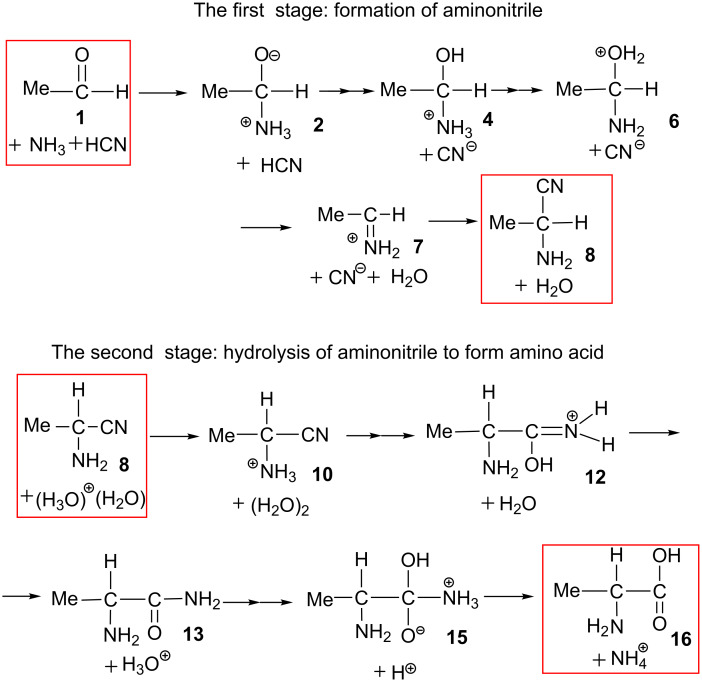
Summary of the present computational work expressed by minimal models.

In this work, we theoretically investigated the whole Strecker reaction shown in Scheme 1(a), which includes two reaction stages. The most favorable pathways are summarized in Scheme 9.

As shown in the upper half of Scheme 9, the first reaction stage, acetaldehyde + NH_3_ + HCN + (H_2_O)_10_ (**1**) → 2-aminopropanonitrile (H_2_O)_11_(**8**), is composed of seven elementary processes. The rate-determining step is the deprotonation of the NH_3_^+^ group in MeCH(OH)-NH_3_^+^ to form 1-aminoethanol, which occurs with an activation energy barrier of 9.6 kcal/mol. The stereochemistry (*R* or *S*) of the product aminonitrile is determined by equal addition of NH_3_ to the carbonyl carbon of the aldehyde in both sides. While the addition of CNˉ to the carbon atom of the protonated imine **7** appears to give the scrambling of the stereochemistry, the water cluster above the imine plane reinforces the CNˉ to attack the carbon atom below the plane; see Scheme 7. While HCN is a very weak acid, CN^−^ may be generated by the proton transfer, HCN + OH^−^ → CN^−^ + H_2_O (**3** → **TS_3/4_** → **4**) in this reaction stage. As shown in the lower half of Scheme 9, the second reaction stage, aminonitrile + H_3_O^+^(H_2_O)_10_ → alanine + NH_4_^+^(H_2_O)_9_, is also composed of seven elementary processes. In this reaction stage, the protonation to the amino nitrogen occurs first, which enhances the subsequent hydrolysis of the cyano group to form an imine C(OH)=NH moiety. Its rate-determining step is the hydrolysis of the cyano group of N(amino)-protonated aminonitrile to afford a N(amino)-protonated 1-hydroxypropanimine **11**. The Δ*E*^≠^ value of this step is 34.7 kcal/mol and the large value corresponds to the high temperature conditions in Scheme 1(b).

## Supporting Information

Suppoting Information File 1:

File 1 File Format: PDF.
Cartesian coordinates of optimized geometries in Figures 1, 3, and 6 and Figures S1–S7.
