# Supplementary material for: Proton transfers in the Strecker reaction revealed by DFT calculations
Source: Beilstein J Org Chem. 2014 Aug 1;10:1765–74. doi: 10.3762/bjoc.10.184 (PMC4143099; doi:10.3762/bjoc.10.184)
Supplement: File 1 — File Format: PDF. Cartesian coordinates of optimized geometries in Figures 1, 3, and 6 and Figures S1–S7. [file Beilstein_J_Org_Chem-10-1765-s001.pdf]

Supporting Information

for

Proton transfers in the Strecker reaction revealed by DFT calculations

Shinichi Yamabe*, Guixiang Zeng, Wei Guan and Shigeyoshi Sakaki

Address: Fukui Institute for Fundamental Chemistry, Kyoto University, Takano-Nishihiraki-cho 34-4, Sakyo-ku, Kyoto 606-8103, JAPAN. Phone: +81-075-711-7907.

Email: Shinichi Yamabe - yamabes@fukui.kyoto-u.ac.jp

*Corresponding author

Cartesian coordinates of optimized geometries in Figures 1, 3, and 6 and Figures S1–S7

Contents

Figure S1: Geometries of the precursor **1**, intermediates and the product (amino-nitrile, **8**).---page s2

Figure S2: The TS geometry of **$TS_{4/5}$-ext** which is an extended model of TS (**$TS_{4/5}$**) in Figure 1 and has the molecular formula $C_3H_{48}N_2O_{21}$.---------------------------page s3

Figure S3: Geometries of the precursor **1**, intermediates and the product (alanine, **16**) along the most favorable route.---page s3

Figure S4: Geometry changes of the species along the Pathway II (Scheme 8) of the 2-amino-nitrile **8** to the amide intermediate **13**.------------------------------------page s4

Figure S5: The other route in B of Pathway I in Scheme 8 from N-protonated α-hydoxyimine **12** to the alanine product **16**. ---page s4

Figure S6: A transition state, **$TS_{10/11}$(Me)**, of iso-CH(Me)$_2$-CN + $(H_2O)_3$ + $(H_2O)_8$ → iso-CH(Me)-C(OH)=NH + $(H_2O)_{10}$. --page s5

Figure S7: Geometries of **$TS_{10/11}$-ext** and **$TS_{8/18}$-ext**, which are extended models of **$TS_{10/11}$** and **$TS_{8/18}$**, respectively. --page s5

Cartesian coordinates--start from page s6

Figure S1: Geometries of the precursor **1**, intermediates and the product (amino-nitrile **8)**.

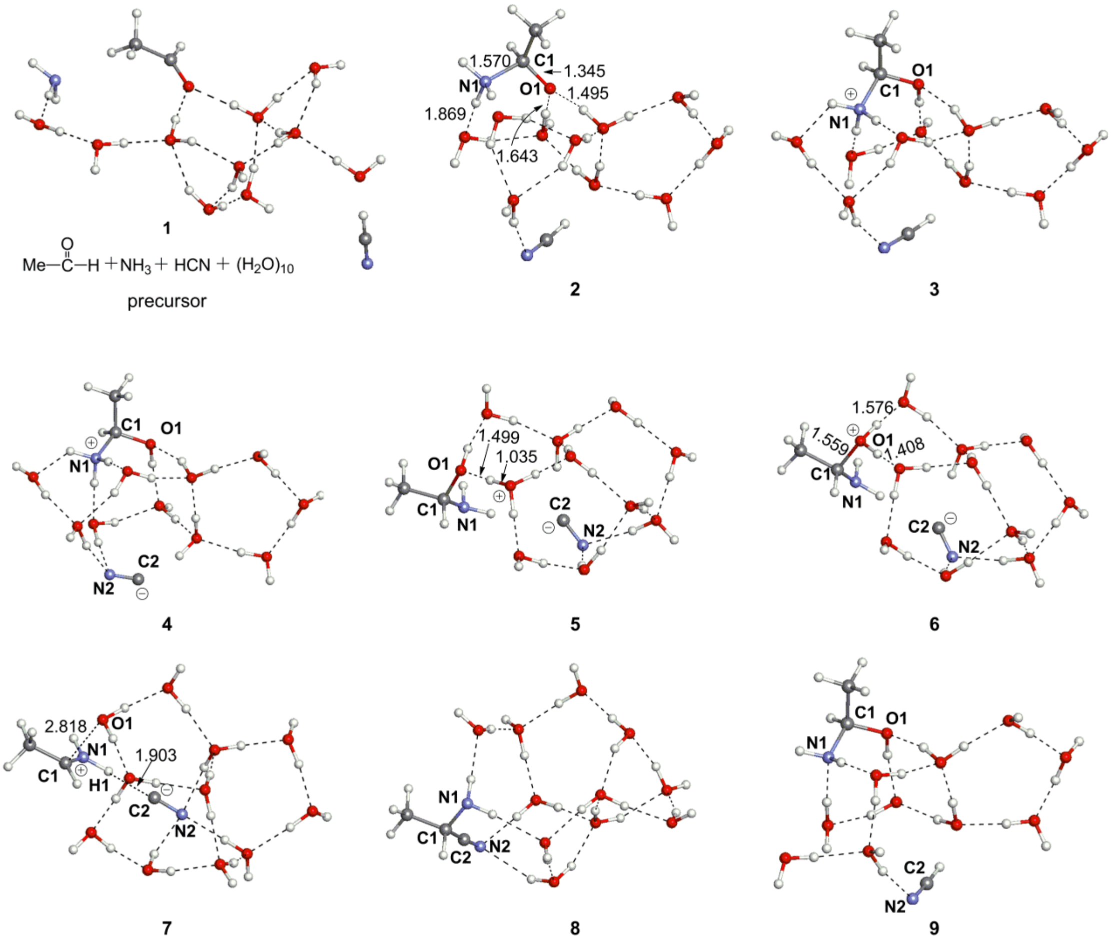

Figure S2: The TS geometry of **$TS_{4/5}$-ext** which is an extended model of TS (**$TS_{4/5}$**) in Figure 1 and has the molecular formula $C_3H_{48}N_2O_{21}$.

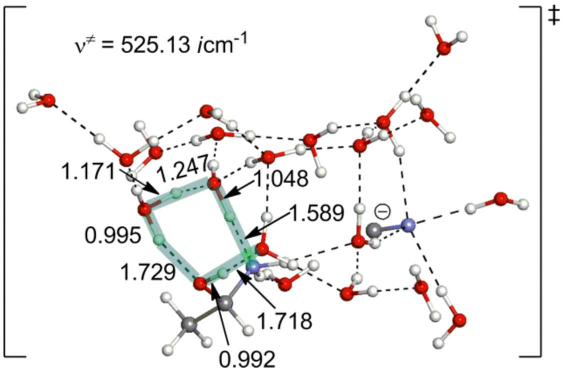

$TS_{4/5}$-ext

Figure S3: Geometries of the precursor **1**, intermediates and the product (alanine, **16**) along the most favorable route.

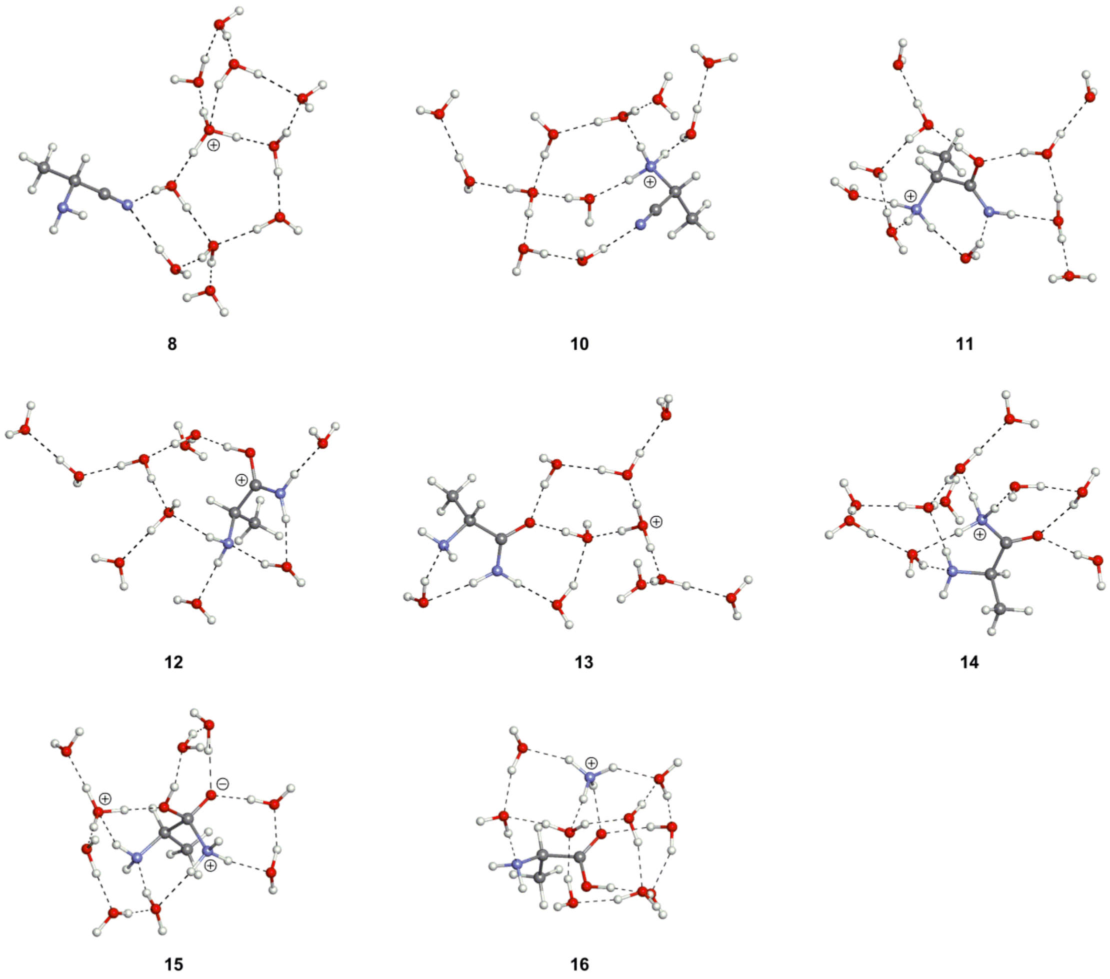

Figure S4: Geometry changes of the species along the pathway II (Scheme 8) of the 2-aminonitrile **8** to the amide intermediate **13**.

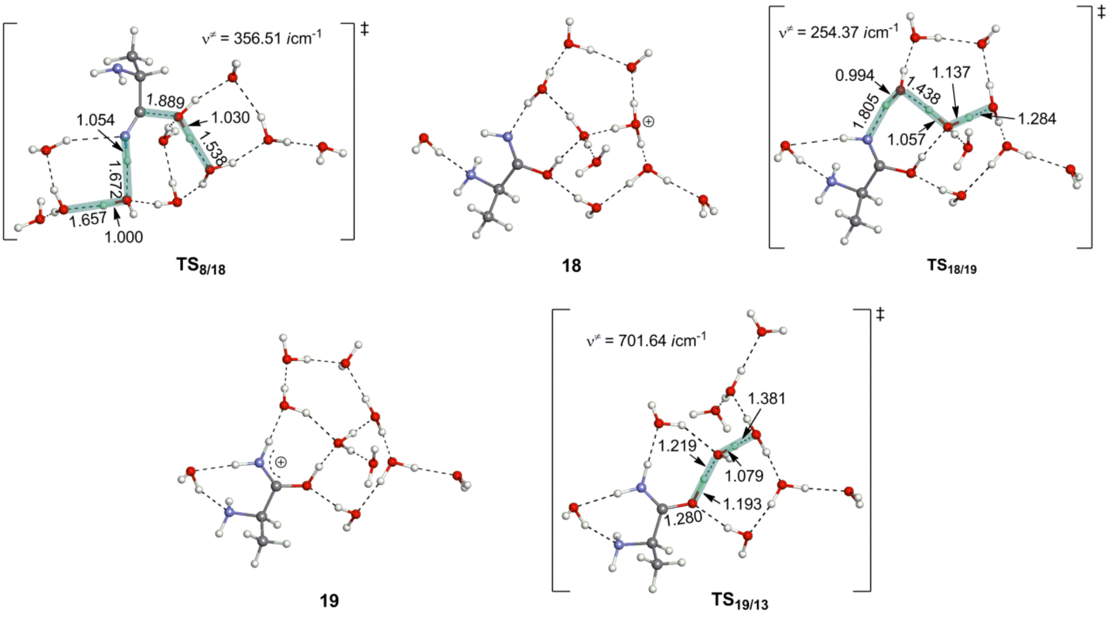

Figure S5: The other route in B of pathway I in Scheme 8 from N-protonated α-hydoxyimine **12** to the alanine product **16**.

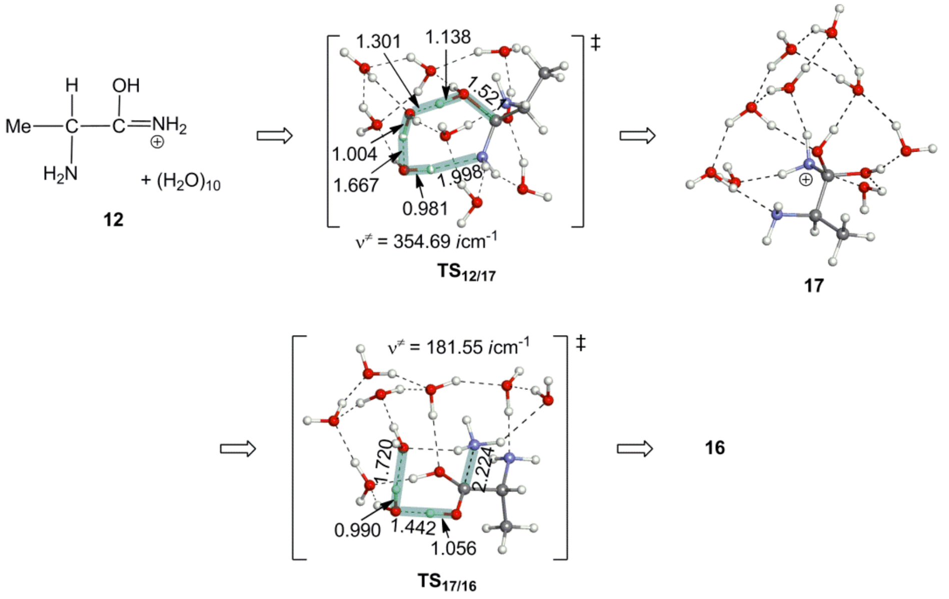

Figure S6: A transition state, **$TS_{10/11}$(Me)**, of iso-CH(Me)$_2$-CN + $(H_2O)_3$ + $(H_2O)_8$ → iso-CH(Me)-C(OH)=NH + $(H_2O)_{10}$. The difference of (E_T + ZPE) between the precursor iso-CH(Me)$_2$-CN + $(H_2O)_3$ + $(H_2O)_8$ and **$TS_{10/11}$(Me)** is +34.56 kcal/mol.

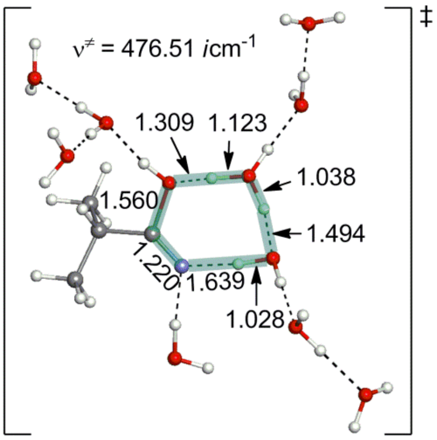

$TS_{10/11}$(Me)

Figure S7: Geometries of **$TS_{10/11}$-ext** and **$TS_{8/18}$-ext**, which are extended models of **$TS_{10/11}$** and **$TS_{8/18}$**, respectively.

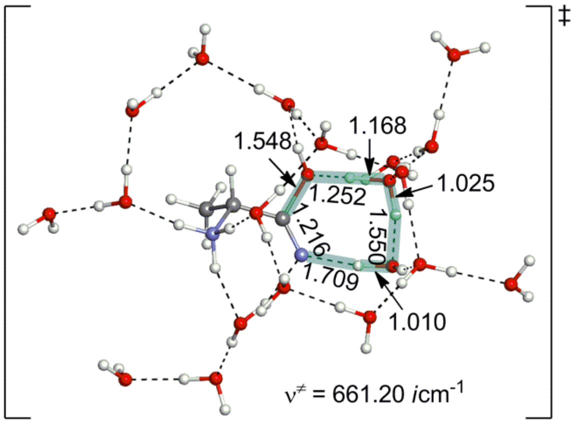

$TS_{10/11}$-ext

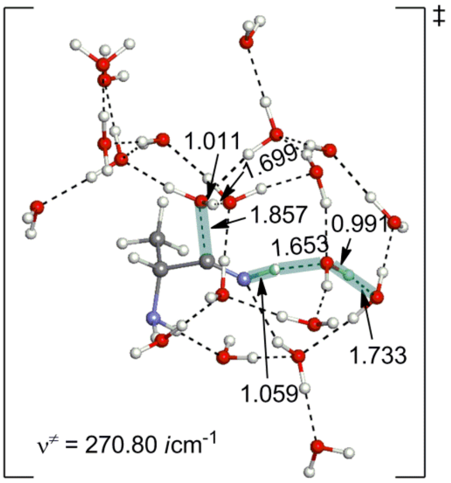

$TS_{8/18}$-ext

Cartesian coordinates of geometries optimized by B3LYP/6-311+G(d,p) scrf=(pcm,solvent=water)

[1] The first reaction in Scheme 5 with stoichiometry $C_3H_{28}N_2O_{11}$. Each species is specified by blue bold numbers. Odd numbers are for precursor, intermediates and product, of which geometries are shown in Figure S1. Even numbers are for transition states, of which ones are in Figure 1.

==blue bold number 1==

str10c.rev.high.log precursor

Standard orientation:

Center Number	Atomic Number	Atomic Type	Coordinates (Angstroms) X	Y	Z
1	6	0	2.407899	2.373791	0.147028
2	8	0	1.509366	1.556362	-0.001754
3	6	0	3.786439	2.217346	-0.376202
4	1	0	4.452349	2.000092	0.475678
5	1	0	3.853966	1.406374	-1.101241
6	1	0	4.125884	3.161372	-0.811776
7	1	0	2.200346	3.289231	0.726268
8	7	0	5.962486	0.977367	2.062320
9	1	0	6.905827	1.350824	2.123057
10	1	0	5.680107	0.751612	3.012228
11	1	0	6.018184	0.098099	1.546085
12	1	0	-0.255038	1.741250	0.524805
13	8	0	-1.212365	1.697310	0.717392
14	1	0	-1.611494	2.502442	0.337357
15	1	0	-2.053426	0.740303	-0.696924
16	8	0	-2.538116	0.535529	-1.520315
17	1	0	-2.065349	-0.233920	-1.898372
18	1	0	-5.863247	-0.607127	0.479334
19	6	0	-6.241987	-0.982099	1.432279
20	7	0	-6.640259	-1.375513	2.437384
21	1	0	1.591727	-0.180890	-0.694386

22	8	0	1.546764	-1.125698	-0.936666
23	1	0	0.842822	-1.204076	-1.604466
24	8	0	-2.468359	3.403790	-1.129472
25	1	0	-2.664737	2.599004	-1.636662
26	1	0	-3.298488	3.890548	-1.076975
27	8	0	-1.018091	-1.739410	-2.099543
28	1	0	-1.065682	-2.163014	-1.210356
29	1	0	-1.240040	-2.417188	-2.748047
30	8	0	-0.612928	-2.462140	0.506857
31	1	0	0.273575	-2.085905	0.375822
32	1	0	-1.060060	-1.852084	1.130777
33	8	0	-5.222378	-0.018186	-1.172325
34	1	0	-4.269976	0.193869	-1.295357
35	1	0	-5.708006	0.652834	-1.664052
36	8	0	-1.783538	-0.579349	2.226588
37	1	0	-1.699639	0.291981	1.790779
38	1	0	-2.709827	-0.661804	2.478524
39	8	0	3.949512	-2.452489	-1.282228
40	1	0	3.739878	-3.392300	-1.250488
41	1	0	3.090736	-1.986809	-1.181568
42	8	0	6.055774	-1.685794	0.378585
43	1	0	6.856438	-1.837843	-0.134437
44	1	0	5.318338	-1.953459	-0.209126

SCF Done: E(RB3LYP) = -1068.70606526 A.U. after 1 cycles

Zero-point correction= 0.356890 (a.u.)

Thermal correction to Energy= 0.397348

Thermal correction to Enthalpy= 0.398292

Thermal correction to Gibbs Free Energy= 0.273255

Sum of electronic and zero-point Energies= -1068.349175

Sum of electronic and thermal Energies= -1068.308718

Sum of electronic and thermal Enthalpies= -1068.307773

Sum of electronic and thermal Free Energies= -1068.432811

E (Thermal)	CV	S
KCal/Mol	Cal/Mol-Kelvin	Cal/Mol-Kelvin

Total 249.339 126.627 263.163

==blue bold number 2==

str10cqq.high.log

Standard orientation:

Center Number	Atomic Number	Atomic Type	Coordinates (Angstroms) X	Y	Z
1	6	0	1.653552	2.832031	0.026374
2	8	0	0.594546	2.201808	0.234970
3	6	0	2.088126	3.187738	-1.367736
4	1	0	3.134841	3.489237	-1.397783
5	1	0	1.914472	2.360196	-2.056708
6	1	0	1.476629	4.038006	-1.692968
7	1	0	2.063071	3.454197	0.830178
8	7	0	3.291569	1.429367	0.479270
9	1	0	4.211858	1.854546	0.551982
10	1	0	3.056542	1.012601	1.382223
11	1	0	3.330222	0.692624	-0.230268
12	1	0	0.062109	1.787989	1.792361
13	8	0	-0.215683	1.333653	2.631509
14	1	0	-0.644369	2.000041	3.180962
15	1	0	-1.278791	-0.015077	1.928891
16	8	0	-1.684386	-0.691750	1.346819
17	1	0	-1.458741	-0.387845	0.436008
18	1	0	-0.733630	-2.318799	1.483358
19	6	0	-0.082170	-3.183709	1.337463
20	7	0	0.628911	-4.063855	1.130858
21	1	0	-0.356298	1.066121	-0.785742
22	8	0	-0.897640	0.316335	-1.118813
23	1	0	-1.678816	0.702984	-1.576054
24	8	0	-4.435992	-0.724600	1.448508
25	1	0	-3.451416	-0.749374	1.457792
26	1	0	-4.726356	-1.628068	1.614773
27	8	0	-3.167788	1.324622	-2.301433

28	1	0	-3.328665	1.118754	-3.228618
29	1	0	-3.974132	1.037993	-1.814038
30	8	0	2.157391	-3.478393	-1.408221
31	1	0	2.435449	-4.231881	-1.942009
32	1	0	1.793724	-3.857065	-0.592184
33	8	0	-5.334621	0.485990	-0.842820
34	1	0	-5.056725	0.050098	-0.004358
35	1	0	-5.983879	1.152685	-0.593852
36	8	0	2.374363	0.468768	3.301277
37	1	0	1.431726	0.709872	3.202561
38	1	0	2.376482	-0.448387	3.595335
39	8	0	0.818328	-1.218471	-2.726595
40	1	0	0.950688	-2.069119	-2.278363
41	1	0	0.169880	-0.717712	-2.189044
42	8	0	3.408307	-0.798664	-1.666379
43	1	0	3.365173	-1.717352	-1.360448
44	1	0	2.563951	-0.716582	-2.152066

SCF Done: E(RB3LYP) = -1068.70979529 A.U. after 1 cycles

	1	2	3
	A	A	A
Frequencies --	-130.6308	13.5670	17.1137

Zero-point correction= 0.360645 (a.u.)

Thermal correction to Energy= 0.397636

Thermal correction to Enthalpy= 0.398581

Thermal correction to Gibbs Free Energy= 0.286687

Sum of electronic and zero-point Energies= -1068.349150

Sum of electronic and thermal Energies= -1068.312159

Sum of electronic and thermal Enthalpies= -1068.311215

Sum of electronic and thermal Free Energies= -1068.423108

	E (Thermal)	CV	S
	KCal/Mol	Cal/Mol-Kelvin	Cal/Mol-Kelvin
Total	249.521	120.615	235.500

==blue bold number 3==

str10cqq.for.high.log

Standard orientation:

Center Number	Atomic Number	Atomic Type	Coordinates (Angstroms) X	Y	Z
1	6	0	1.621030	2.597725	0.228048
2	8	0	0.511706	1.850333	0.367874
3	6	0	1.785214	3.243756	-1.146071
4	1	0	2.720462	3.804777	-1.227975
5	1	0	1.753622	2.485088	-1.932271
6	1	0	0.956392	3.936351	-1.303655
7	1	0	1.725816	3.366503	1.011555
8	7	0	2.883082	1.697477	0.478391
9	1	0	3.746465	2.241840	0.468422
10	1	0	2.793999	1.250844	1.409567
11	1	0	2.961258	0.952793	-0.244293
12	1	0	0.041303	1.455684	1.731284
13	8	0	-0.256261	1.024619	2.627349
14	1	0	-0.737728	1.692435	3.128122
15	1	0	-1.262655	-0.295982	1.884432
16	8	0	-1.676377	-0.949246	1.276003
17	1	0	-1.450340	-0.606511	0.377150
18	1	0	-0.650669	-2.533357	1.333973
19	6	0	0.092226	-3.315457	1.155934
20	7	0	0.904220	-4.094460	0.917650
21	1	0	-0.310199	0.883005	-0.675477
22	8	0	-0.866944	0.175633	-1.116894
23	1	0	-1.625704	0.621062	-1.550228
24	8	0	-4.417063	-0.912755	1.385869
25	1	0	-3.432301	-0.967194	1.386819
26	1	0	-4.734156	-1.818356	1.472392
27	8	0	-3.124251	1.349337	-2.252093
28	1	0	-3.313069	1.164219	-3.178344
29	1	0	-3.928039	1.079061	-1.752660

30	8	0	2.528300	-3.164782	-1.440420
31	1	0	3.001519	-3.833469	-1.949244
32	1	0	2.148615	-3.634736	-0.680650
33	8	0	-5.293183	0.553233	-0.759654
34	1	0	-5.018889	0.021456	0.022947
35	1	0	-5.861594	1.252095	-0.418235
36	8	0	2.409746	0.630442	3.130252
37	1	0	1.427059	0.675485	3.131220
38	1	0	2.638366	-0.273277	3.373344
39	8	0	0.900273	-1.125734	-2.800800
40	1	0	1.150894	-1.974813	-2.402720
41	1	0	0.210183	-0.735983	-2.218203
42	8	0	3.249299	-0.303359	-1.547448
43	1	0	3.376201	-1.217247	-1.248041
44	1	0	2.449299	-0.390800	-2.109282

SCF Done: E(RB3LYP) = -1068.71956246 A.U. after 1 cycles

Zero-point correction= 0.364832 (a.u.)

Thermal correction to Energy= 0.400002

Thermal correction to Enthalpy= 0.400946

Thermal correction to Gibbs Free Energy= 0.294474

Sum of electronic and zero-point Energies= -1068.354730

Sum of electronic and thermal Energies= -1068.319561

Sum of electronic and thermal Enthalpies= -1068.318617

Sum of electronic and thermal Free Energies= -1068.425088

	E (Thermal)	CV	S
	KCal/Mol	Cal/Mol-Kelvin	Cal/Mol-Kelvin
Total	251.005	116.545	224.088

==blue bold numer 4==

str10bf.high.log

Standard orientation:

Center Atomic Atomic Coordinates (Angstroms)

Number	Number	Type	X	Y	Z
1	6	0	-1.359004	-2.421279	0.744385
2	8	0	-0.057596	-1.986006	0.758761
3	6	0	-1.582893	-3.550843	-0.249715
4	1	0	-2.628713	-3.868051	-0.268307
5	1	0	-1.286243	-3.240388	-1.254599
6	1	0	-0.969677	-4.403856	0.044566
7	1	0	-1.702390	-2.720485	1.743336
8	7	0	-2.269522	-1.241185	0.369314
9	1	0	-2.214247	-0.515132	1.110933
10	1	0	-1.966859	-0.830916	-0.540764
11	1	0	-3.257155	-1.485546	0.268086
12	1	0	0.222899	-1.327693	1.702297
13	8	0	0.508764	-0.519134	2.591706
14	1	0	0.940955	-0.955905	3.332599
15	1	0	1.372460	0.633626	1.688019
16	8	0	1.748207	1.232334	0.983854
17	1	0	1.619699	0.711804	0.158649
18	1	0	0.485572	2.593819	0.657440
19	6	0	-0.379821	3.156840	0.293156
20	7	0	-1.313741	3.682887	-0.123491
21	1	0	0.771061	-1.146361	-0.554218
22	8	0	1.151516	-0.457588	-1.151428
23	1	0	1.954518	-0.832785	-1.573483
24	8	0	4.458515	1.536685	1.096021
25	1	0	3.472282	1.475535	1.098634
26	1	0	4.667254	2.474434	1.024960
27	8	0	3.529119	-1.387358	-2.220702
28	1	0	3.700520	-1.258403	-3.159759
29	1	0	4.273165	-0.951154	-1.746091
30	8	0	-3.107466	2.172485	-1.911116
31	1	0	-3.462216	2.646188	-2.673006
32	1	0	-2.671393	2.843487	-1.356982
33	8	0	5.522327	-0.128555	-0.806908
34	1	0	5.173897	0.477394	-0.112278

35	1	0	6.167129	-0.692918	-0.366761
36	8	0	-1.965740	0.379383	2.693126
37	1	0	-1.002937	0.139400	2.807771
38	1	0	-2.022950	1.338675	2.751600
39	8	0	-1.434915	-0.142166	-2.121590
40	1	0	-1.828011	0.745943	-2.179352
41	1	0	-0.472342	-0.045067	-1.980910
42	8	0	-4.675240	0.128937	-0.629504
43	1	0	-4.239477	0.892027	-1.051544
44	1	0	-5.503582	0.469955	-0.276313

	1	2	3
	A	A	A
Frequencies --	-631.2032	18.0210	20.4523

Zero-point correction= 0.361135 (a.u.)

Thermal correction to Energy= 0.395872

Thermal correction to Enthalpy= 0.396816

Thermal correction to Gibbs Free Energy= 0.291440

Sum of electronic and zero-point Energies= -1068.354652

Sum of electronic and thermal Energies= -1068.319915

Sum of electronic and thermal Enthalpies= -1068.318971

Sum of electronic and thermal Free Energies= -1068.424347

	E (Thermal)	CV	S
	KCal/Mol	Cal/Mol-Kelvin	Cal/Mol-Kelvin
Total	248.414	115.316	221.783

==bold blue 5==

str10bf.for.high.log

Standard orientation:

Center	Atomic	Atomic	Coordinates (Angstroms)		
Number	Number	Type	X	Y	Z

1	6	0	-1.502942	-2.333693	0.800160
2	8	0	-0.142723	-2.053190	0.771225
3	6	0	-1.844394	-3.457492	-0.160317
4	1	0	-2.916209	-3.666855	-0.157070
5	1	0	-1.534242	-3.201068	-1.175954
6	1	0	-1.315554	-4.359985	0.148582
7	1	0	-1.838618	-2.571588	1.814602
8	7	0	-2.263829	-1.073713	0.411878
9	1	0	-2.140723	-0.349160	1.153715
10	1	0	-1.911585	-0.713139	-0.503021
11	1	0	-3.273858	-1.205446	0.293411
12	1	0	0.148097	-1.462540	1.585087
13	8	0	0.540825	-0.460137	2.596083
14	1	0	0.978009	-0.819157	3.374498
15	1	0	1.368309	0.567931	1.672304
16	8	0	1.765909	1.163454	0.955390
17	1	0	1.654487	0.635673	0.135861
18	1	0	0.517418	2.500104	0.605110
19	6	0	-0.344816	3.074115	0.242241
20	7	0	-1.274634	3.610007	-0.171564
21	1	0	0.818153	-1.248377	-0.628109
22	8	0	1.200107	-0.555509	-1.204220
23	1	0	2.017900	-0.919806	-1.610180
24	8	0	4.456968	1.495634	1.104786
25	1	0	3.469668	1.421724	1.092698
26	1	0	4.655184	2.435766	1.035605
27	8	0	3.600250	-1.442409	-2.221861
28	1	0	3.783439	-1.313644	-3.158739
29	1	0	4.330896	-0.993962	-1.737129
30	8	0	-3.100246	2.135665	-1.954461
31	1	0	-3.461829	2.614068	-2.710210
32	1	0	-2.660005	2.804013	-1.400091
33	8	0	5.554942	-0.153470	-0.787625
34	1	0	5.193933	0.448853	-0.095590
35	1	0	6.205654	-0.707674	-0.343315
36	8	0	-1.841846	0.507760	2.693841

37	1	0	-0.876144	0.210684	2.791344
38	1	0	-1.850066	1.469073	2.742678
39	8	0	-1.398554	-0.153555	-2.121381
40	1	0	-1.786437	0.732931	-2.222374
41	1	0	-0.431216	-0.064209	-2.025099
42	8	0	-4.689255	0.188869	-0.535533
43	1	0	-4.257418	0.890138	-1.058060
44	1	0	-5.536108	0.029124	-0.965232

SCF Done: E(RB3LYP) = -1068.71649445   A.U. after   1 cycles

Zero-point correction=   0.364092 (a.u.)
Thermal correction to Energy=   0.398941
Thermal correction to Enthalpy=   0.399885
Thermal correction to Gibbs Free Energy=   0.294930
Sum of electronic and zero-point Energies=   -1068.352403
Sum of electronic and thermal Energies=   -1068.317554
Sum of electronic and thermal Enthalpies=   -1068.316609
Sum of electronic and thermal Free Energies=   -1068.421564

	E (Thermal)	CV	S
	KCal/Mol	Cal/Mol-Kelvin	Cal/Mol-Kelvin
Total	250.339	115.923	220.897

==boild blue 6==

str11aa.high.log

Standard orientation:

Center Number	Atomic Number	Atomic Type	Coordinates (Angstroms) X	Y	Z
1	6	0	-1.631985	-2.252955	0.955899
2	8	0	-0.247802	-2.063996	0.891488
3	6	0	-2.031621	-3.442670	0.106874
4	1	0	-3.112738	-3.592008	0.129198
5	1	0	-1.711675	-3.300057	-0.927666

6	1	0	-1.551004	-4.338128	0.502149
7	1	0	-1.965445	-2.375145	1.989520
8	7	0	-2.310065	-0.995406	0.449708
9	1	0	-2.160490	-0.218670	1.127542
10	1	0	-1.927969	-0.731331	-0.489238
11	1	0	-3.325653	-1.079304	0.326228
12	1	0	0.070527	-1.435878	1.599281
13	8	0	0.642758	-0.165444	2.536159
14	1	0	1.211734	-0.369959	3.285604
15	1	0	1.206584	0.470449	1.791101
16	8	0	1.748122	1.187529	0.834098
17	1	0	1.682028	0.605817	0.056871
18	1	0	0.683123	2.305527	0.449770
19	6	0	-0.178688	2.970580	0.079610
20	7	0	-1.085525	3.543752	-0.344283
21	1	0	0.832327	-1.439196	-0.644969
22	8	0	1.187679	-0.806493	-1.293138
23	1	0	2.041080	-1.165182	-1.621486
24	8	0	4.333712	1.560723	1.035193
25	1	0	3.329281	1.463196	0.976144
26	1	0	4.525366	2.489985	0.870379
27	8	0	3.684131	-1.662592	-2.094750
28	1	0	3.923244	-1.587649	-3.024828
29	1	0	4.366056	-1.154724	-1.596822
30	8	0	-2.950845	2.083248	-2.050788
31	1	0	-3.282061	2.563084	-2.819334
32	1	0	-2.458560	2.735651	-1.517941
33	8	0	5.511055	-0.218862	-0.644500
34	1	0	5.111342	0.439526	-0.023060
35	1	0	6.146426	-0.722847	-0.124811
36	8	0	-1.856896	0.754222	2.622115
37	1	0	-0.896900	0.544376	2.750356
38	1	0	-1.929416	1.714323	2.597024
39	8	0	-1.407592	-0.318303	-2.124321
40	1	0	-1.736403	0.584931	-2.275698
41	1	0	-0.432632	-0.293414	-2.068028

42	8	0	-4.660423	0.311070	-0.560231
43	1	0	-4.188132	0.961131	-1.114068
44	1	0	-5.498347	0.148518	-1.006080

SCF Done: E(RB3LYP) = -1068.71133625   A.U. after   1 cycles

	1	2	3
	A	A	A
Frequencies --	-358.5591	20.9442	27.9909

Zero-point correction=   0.361204 (a.u.)

Thermal correction to Energy=   0.395328

Thermal correction to Enthalpy=   0.396272

Thermal correction to Gibbs Free Energy=   0.293323

Sum of electronic and zero-point Energies=   -1068.350132

Sum of electronic and thermal Energies=   -1068.316008

Sum of electronic and thermal Enthalpies=   -1068.315064

Sum of electronic and thermal Free Energies=   -1068.418013

	E (Thermal)	CV	S
	KCal/Mol	Cal/Mol-Kelvin	Cal/Mol-Kelvin
Total	248.072	113.940	216.675

==bold blue 7==

str11aa.rev.high.log

Standard orientation:

Center Number	Atomic Number	Atomic Type	Coordinates (Angstroms) X	Y	Z
1	6	0	1.762798	2.163157	1.155261
2	8	0	0.375231	1.946353	1.193147
3	6	0	2.056069	3.468047	0.444825
4	1	0	3.132084	3.638613	0.377118
5	1	0	1.631695	3.459998	-0.561694
6	1	0	1.609968	4.288294	1.007835

7	1	0	2.185160	2.155013	2.162412
8	7	0	2.400407	1.007928	0.421471
9	1	0	2.263278	0.116575	0.951151
10	1	0	1.974738	0.891741	-0.529210
11	1	0	3.412481	1.102142	0.272763
12	1	0	0.137017	1.280488	1.875694
13	8	0	-0.447696	-0.181411	2.764255
14	1	0	-0.865189	-0.119201	3.631023
15	1	0	-1.091612	-0.624350	2.158442
16	8	0	-1.906373	-1.319658	0.748269
17	1	0	-1.705453	-0.623857	0.080115
18	1	0	-1.352376	-2.098042	0.489237
19	6	0	0.089118	-3.280045	-0.032186
20	7	0	1.212957	-3.313221	-0.355850
21	1	0	-0.807201	1.391949	-0.291832
22	8	0	-1.225505	0.786079	-0.928866
23	1	0	-2.022789	1.241624	-1.288128
24	8	0	-4.651033	-1.562834	0.727553
25	1	0	-3.666668	-1.556923	0.742250
26	1	0	-4.906851	-2.448857	0.448668
27	8	0	-3.526906	1.940385	-1.841131
28	1	0	-3.665399	2.005451	-2.792242
29	1	0	-4.306183	1.455151	-1.483303
30	8	0	2.843085	-1.848375	-2.091219
31	1	0	3.096025	-2.365441	-2.864757
32	1	0	2.347654	-2.463341	-1.496825
33	8	0	-5.613702	0.528012	-0.767222
34	1	0	-5.304962	-0.230616	-0.219996
35	1	0	-6.237341	1.015197	-0.217875
36	8	0	1.978303	-1.346468	1.922071
37	1	0	1.158280	-1.115004	2.397945
38	1	0	1.723039	-2.055254	1.305876
39	8	0	1.308811	0.531153	-2.126474
40	1	0	1.671230	-0.360149	-2.288540
41	1	0	0.354327	0.442035	-1.943926
42	8	0	4.721650	-0.179739	-0.750357

43	1	0	4.184257	-0.806365	-1.274444
44	1	0	5.462414	0.065254	-1.314702

SCF Done: E(RB3LYP) = -1068.72945013 A.U. after 1 cycles

Zero-point correction= 0.368279 (a.u.)

Thermal correction to Energy= 0.403358

Thermal correction to Enthalpy= 0.404303

Thermal correction to Gibbs Free Energy= 0.298101

Sum of electronic and zero-point Energies= -1068.361172

Sum of electronic and thermal Energies= -1068.326092

Sum of electronic and thermal Enthalpies= -1068.325148

Sum of electronic and thermal Free Energies= -1068.431349

	E (Thermal)	CV	S
	KCal/Mol	Cal/Mol-Kelvin	Cal/Mol-Kelvin
Total	253.111	116.563	223.520

==bold blue 8==

str09dk.high.log

Standard orientation:

Center Number	Atomic Number	Atomic Type	Coordinates (Angstroms) X	Y	Z
1	6	0	2.539728	0.254048	1.445647
2	7	0	1.095022	0.383917	1.231338
3	6	0	3.070152	1.338648	2.364071
4	1	0	2.819126	2.330641	1.979276
5	1	0	2.635578	1.232019	3.360011
6	1	0	4.154200	1.258215	2.450274
7	1	0	2.789502	-0.734330	1.840012
8	8	0	3.241658	0.354676	0.178215
9	1	0	3.031478	-0.451507	-0.388601
10	1	0	3.084062	1.499814	-0.560993
11	1	0	0.654261	-0.523492	1.064952

12	1	0	0.653391	0.776369	2.058071
13	8	0	2.809377	2.387596	-1.132178
14	1	0	1.747952	2.288912	-1.212014
15	1	0	3.225511	2.380753	-2.005842
16	8	0	0.425562	1.822988	-1.027575
17	1	0	0.539043	1.338357	-0.153984
18	1	0	-0.326870	2.465431	-0.939162
19	6	0	-3.108681	0.091578	2.444524
20	7	0	-2.786689	-1.011279	2.215760
21	8	0	1.703425	-3.628784	0.472622
22	1	0	1.551900	-4.551126	0.239718
23	1	0	0.824748	-3.258177	0.705178
24	8	0	2.591739	-1.696130	-1.327084
25	1	0	2.301955	-2.452004	-0.772328
26	1	0	1.797587	-1.392861	-1.822620
27	8	0	-1.905303	-2.011429	-1.770405
28	1	0	-2.735339	-1.576221	-1.475287
29	1	0	-2.169778	-2.807289	-2.246292
30	8	0	0.387764	-0.583704	-2.626719
31	1	0	0.303021	0.284952	-2.193389
32	1	0	-0.440459	-1.053964	-2.413506
33	1	0	-3.817643	-0.929266	0.394005
34	8	0	-4.046898	-0.755957	-0.540576
35	1	0	-4.932190	-1.118133	-0.669393
36	1	0	-3.548003	1.644597	1.163380
37	8	0	-3.756057	2.040856	0.291454
38	1	0	-3.960237	1.259694	-0.250724
39	1	0	-1.335942	-1.938328	1.372826
40	8	0	-0.618045	-2.220127	0.768101
41	1	0	-1.022020	-2.215262	-0.120850
42	8	0	-1.699017	3.447267	-0.822997
43	1	0	-2.453235	2.977536	-0.391460
44	1	0	-1.610764	4.292546	-0.369266

SCF Done: E(RB3LYP) = -1068.69883053   A.U. after   1 cycles

	1	2	3
	A	A	A
Frequencies --	-195.4082	28.8791	34.6878

Zero-point correction= 0.364928 (a.u.)

Thermal correction to Energy= 0.397738

Thermal correction to Enthalpy= 0.398682

Thermal correction to Gibbs Free Energy= 0.300167

Sum of electronic and zero-point Energies= -1068.333903

Sum of electronic and thermal Energies= -1068.301093

Sum of electronic and thermal Enthalpies= -1068.300149

Sum of electronic and thermal Free Energies= -1068.398664

	E (Thermal)	CV	S
	KCal/Mol	Cal/Mol-Kelvin	Cal/Mol-Kelvin
Total	249.584	111.473	207.342

==bold blue 9==

str09ex.for.high.log

Standard orientation:

Center Number	Atomic Number	Atomic Type	Coordinates (Angstroms) X	Y	Z
1	6	0	3.755764	0.196230	0.734049
2	7	0	2.865440	0.663624	1.759009
3	6	0	5.124792	0.849980	0.852054
4	1	0	5.040045	1.937141	0.760208
5	1	0	5.570410	0.618692	1.821462
6	1	0	5.786543	0.487508	0.064399
7	1	0	3.844225	-0.888546	0.817127
8	8	0	3.246854	0.390697	-0.630250
9	1	0	2.347452	-0.627828	-1.263288
10	1	0	2.903588	1.311404	-0.732641
11	1	0	1.931380	0.271457	1.677080
12	1	0	2.785881	1.676190	1.755108

13	8	0	2.047368	2.835881	-0.858440
14	1	0	1.100206	2.604125	-0.719686
15	1	0	2.088895	3.315171	-1.693360
16	8	0	-0.484951	1.892019	-0.369240
17	1	0	-0.488905	1.410961	0.492820
18	1	0	-1.282380	2.471669	-0.366053
19	6	0	-0.722117	0.093447	1.923125
20	7	0	-1.362613	-0.838619	2.225357
21	8	0	1.512191	-3.429503	-0.317568
22	1	0	0.596671	-3.456684	0.069985
23	1	0	1.660184	-4.276283	-0.753744
24	8	0	1.736337	-1.311697	-1.742596
25	1	0	1.665594	-2.171456	-1.194548
26	1	0	0.789842	-0.902196	-1.875923
27	8	0	-2.623619	-1.977909	-1.261416
28	1	0	-3.228565	-1.573270	-0.602036
29	1	0	-3.180893	-2.384358	-1.935419
30	8	0	-0.542833	-0.266825	-2.076598
31	1	0	-0.602705	0.538914	-1.510407
32	1	0	-1.294634	-0.843139	-1.825021
33	1	0	-3.106164	-0.822586	1.496332
34	8	0	-3.906886	-0.832955	0.923404
35	1	0	-4.553315	-1.388701	1.376215
36	1	0	-5.524889	1.982085	0.786837
37	8	0	-4.841938	1.700897	0.167991
38	1	0	-4.542860	0.822278	0.480734
39	1	0	-1.048472	-2.575327	1.305584
40	8	0	-0.979399	-3.308221	0.662949
41	1	0	-1.553772	-3.023297	-0.072935
42	8	0	-2.767545	3.413641	-0.412237
43	1	0	-3.536706	2.849950	-0.170038
44	1	0	-2.801719	4.174936	0.177294

SCF Done: E(RB3LYP) = -1068.71119445   A.U. after   1 cycles

Zero-point correction=   0.365311 (a.u.)

Thermal correction to Energy= 0.399584

Thermal correction to Enthalpy= 0.400528

Thermal correction to Gibbs Free Energy= 0.296806

Sum of electronic and zero-point Energies= -1068.345884

Sum of electronic and thermal Energies= -1068.311610

Sum of electronic and thermal Enthalpies= -1068.310666

Sum of electronic and thermal Free Energies= -1068.414389

	E (Thermal)	CV	S
	KCal/Mol	Cal/Mol-Kelvin	Cal/Mol-Kelvin
Total	250.743	114.667	218.303

==bold blue 10==

str09ex.high.log

Standard orientation:

Center Number	Atomic Number	Atomic Type	Coordinates (Angstroms) X	Y	Z
1	6	0	3.645234	0.273246	0.812174
2	7	0	2.687922	0.732734	1.743051
3	6	0	5.008577	0.906535	1.008514
4	1	0	4.942091	1.995584	0.936555
5	1	0	5.390753	0.647176	1.997070
6	1	0	5.708328	0.548301	0.253558
7	1	0	3.699830	-0.814319	0.840967
8	8	0	3.256636	0.529044	-0.640806
9	1	0	2.622518	-0.287527	-1.112296
10	1	0	2.805631	1.426286	-0.741944
11	1	0	1.760901	0.329535	1.644862
12	1	0	2.623161	1.743337	1.793566
13	8	0	1.977341	2.802531	-0.843781
14	1	0	1.018402	2.589525	-0.726368
15	1	0	2.054784	3.301064	-1.665186
16	8	0	-0.538579	1.906363	-0.407268
17	1	0	-0.556959	1.417316	0.448840

18	1	0	-1.360141	2.449255	-0.441425
19	6	0	-0.754167	0.077712	1.874383
20	7	0	-1.393581	-0.853937	2.179577
21	8	0	1.706963	-3.429224	-0.249742
22	1	0	0.779487	-3.447844	0.094404
23	1	0	1.856553	-4.281217	-0.674138
24	8	0	1.940088	-1.200085	-1.713373
25	1	0	1.878551	-2.050262	-1.201379
26	1	0	1.006829	-0.891710	-1.896043
27	8	0	-2.550980	-2.079282	-1.324851
28	1	0	-3.175554	-1.683106	-0.679905
29	1	0	-3.088476	-2.528325	-1.987627
30	8	0	-0.512150	-0.270455	-2.171491
31	1	0	-0.606200	0.518418	-1.595689
32	1	0	-1.236850	-0.874849	-1.919388
33	1	0	-3.115783	-0.926710	1.422763
34	8	0	-3.905760	-0.937925	0.834673
35	1	0	-4.551740	-1.512616	1.264023
36	1	0	-5.233129	1.936704	1.322204
37	8	0	-4.874098	1.673764	0.467289
38	1	0	-4.536349	0.762802	0.595421
39	1	0	-0.976603	-2.586562	1.262574
40	8	0	-0.860967	-3.315536	0.623819
41	1	0	-1.424540	-3.051774	-0.128052
42	8	0	-2.902821	3.275478	-0.606712
43	1	0	-3.634617	2.760447	-0.197901
44	1	0	-2.994512	4.177144	-0.280353

SCF Done: E(RB3LYP) = -1068.70853849 A.U. after 1 cycles

	1	2	3
	A	A	A
Frequencies --	-238.9260	16.3289	20.9937

Zero-point correction= 0.362765 (a.u.)

Thermal correction to Energy= 0.396883

Thermal correction to Enthalpy= 0.397827

Thermal correction to Gibbs Free Energy= 0.293798

Sum of electronic and zero-point Energies= -1068.345774

Sum of electronic and thermal Energies= -1068.311656

Sum of electronic and thermal Enthalpies= -1068.310712

Sum of electronic and thermal Free Energies= -1068.414740

	E (Thermal)	CV	S
	KCal/Mol	Cal/Mol-Kelvin	Cal/Mol-Kelvin
Total	249.048	114.251	218.946

==bold blue 11==

str09ex.rev.high.log

Standard orientation:

Center Number	Atomic Number	Atomic Type	Coordinates (Angstroms) X	Y	Z
1	6	0	3.755764	0.196230	0.734049
2	7	0	2.865440	0.663624	1.759009
3	6	0	5.124792	0.849980	0.852054
4	1	0	5.040045	1.937141	0.760208
5	1	0	5.570410	0.618692	1.821462
6	1	0	5.786543	0.487508	0.064399
7	1	0	3.844225	-0.888546	0.817127
8	8	0	3.246854	0.390697	-0.630250
9	1	0	2.347452	-0.627828	-1.263288
10	1	0	2.903588	1.311404	-0.732641
11	1	0	1.931380	0.271457	1.677080
12	1	0	2.785881	1.676190	1.755108
13	8	0	2.047368	2.835881	-0.858440
14	1	0	1.100206	2.604125	-0.719686
15	1	0	2.088895	3.315171	-1.693360
16	8	0	-0.484951	1.892019	-0.369240
17	1	0	-0.488905	1.410961	0.492820
18	1	0	-1.282380	2.471669	-0.366053

19	6	0	-0.722117	0.093447	1.923125
20	7	0	-1.362613	-0.838619	2.225357
21	8	0	1.512191	-3.429503	-0.317568
22	1	0	0.596671	-3.456684	0.069985
23	1	0	1.660184	-4.276283	-0.753744
24	8	0	1.736337	-1.311697	-1.742596
25	1	0	1.665594	-2.171456	-1.194548
26	1	0	0.789842	-0.902196	-1.875923
27	8	0	-2.623619	-1.977909	-1.261416
28	1	0	-3.228565	-1.573270	-0.602036
29	1	0	-3.180893	-2.384358	-1.935419
30	8	0	-0.542833	-0.266825	-2.076598
31	1	0	-0.602705	0.538914	-1.510407
32	1	0	-1.294634	-0.843139	-1.825021
33	1	0	-3.106164	-0.822586	1.496332
34	8	0	-3.906886	-0.832955	0.923404
35	1	0	-4.553315	-1.388701	1.376215
36	1	0	-5.524889	1.982085	0.786837
37	8	0	-4.841938	1.700897	0.167991
38	1	0	-4.542860	0.822278	0.480734
39	1	0	-1.048472	-2.575327	1.305584
40	8	0	-0.979399	-3.308221	0.662949
41	1	0	-1.553772	-3.023297	-0.072935
42	8	0	-2.767545	3.413641	-0.412237
43	1	0	-3.536706	2.849950	-0.170038
44	1	0	-2.801719	4.174936	0.177294

SCF Done: E(RB3LYP) = -1068.71119445 A.U. after 1 cycles

Zero-point correction= 0.365311 (a.u.)
Thermal correction to Energy= 0.399584
Thermal correction to Enthalpy= 0.400528
Thermal correction to Gibbs Free Energy= 0.296806
Sum of electronic and zero-point Energies= -1068.345884
Sum of electronic and thermal Energies= -1068.311610
Sum of electronic and thermal Enthalpies= -1068.310666

Sum of electronic and thermal Free Energies= -1068.414389

	E (Thermal)	CV	S
	KCal/Mol	Cal/Mol-Kelvin	Cal/Mol-Kelvin
Total	250.743	114.667	218.303

==bold blue 12==

str09fyyy.high.log

Standard orientation:

Center Number	Atomic Number	Atomic Type	Coordinates (Angstroms) X	Y	Z
1	6	0	3.648250	-0.474823	-0.668660
2	7	0	2.998742	-1.034378	-1.733107
3	6	0	5.114282	-0.805959	-0.543781
4	1	0	5.270368	-1.886782	-0.573655
5	1	0	5.649664	-0.352321	-1.380761
6	1	0	5.522349	-0.415227	0.387347
7	1	0	3.420105	0.582904	-0.561937
8	8	0	3.038088	-1.004171	0.793534
9	1	0	2.571425	-0.232995	1.297583
10	1	0	2.372222	-1.750562	0.668709
11	1	0	2.043441	-0.740717	-1.939833
12	1	0	3.202712	-2.000866	-1.947780
13	8	0	1.265455	-2.928642	0.569280
14	1	0	0.353760	-2.550228	0.546963
15	1	0	1.276491	-3.577809	1.281871
16	8	0	-1.134234	-1.640630	0.429903
17	1	0	-1.138474	-1.158198	-0.416678
18	1	0	-1.991137	-2.128838	0.478782
19	6	0	-0.026025	-0.093447	-2.339087
20	7	0	-1.114964	0.196195	-2.018734
21	8	0	2.252905	3.055652	0.336297
22	1	0	1.383726	3.117255	-0.126316
23	1	0	2.460661	3.945362	0.641732

24	8	0	1.935100	0.929987	2.031480
25	1	0	2.057264	1.753959	1.506514
26	1	0	0.959241	0.807904	2.129414
27	8	0	-2.325523	2.496862	0.924240
28	1	0	-2.926731	2.072306	0.272963
29	1	0	-2.849083	3.151550	1.400354
30	8	0	-0.760527	0.487398	2.242111
31	1	0	-0.944935	-0.287944	1.673549
32	1	0	-1.313948	1.202909	1.876683
33	1	0	-2.739504	0.769744	-1.529799
34	8	0	-3.579493	1.121688	-1.135005
35	1	0	-3.977318	1.685673	-1.809415
36	1	0	-5.957655	-1.059696	-0.793553
37	8	0	-5.252762	-0.882407	-0.161037
38	1	0	-4.709712	-0.166538	-0.555279
39	1	0	-0.297196	2.175380	-1.363449
40	8	0	-0.195406	3.010301	-0.877799
41	1	0	-0.904370	2.980072	-0.206156
42	8	0	-3.553505	-2.884140	0.622043
43	1	0	-4.227995	-2.236458	0.313414
44	1	0	-3.691644	-3.682369	0.100400

SCF Done: E(RB3LYP) = -1068.70854957 A.U. after 1 cycles

	1	2	3
	A	A	A
Frequencies --	-95.8055	16.7764	27.2612

Zero-point correction= 0.364956 (a.u.)

Thermal correction to Energy= 0.399340

Thermal correction to Enthalpy= 0.400284

Thermal correction to Gibbs Free Energy= 0.296755

Sum of electronic and zero-point Energies= -1068.343593

Sum of electronic and thermal Energies= -1068.309210

Sum of electronic and thermal Enthalpies= -1068.308266

Sum of electronic and thermal Free Energies= -1068.411794

	E (Thermal)	CV	S
	KCal/Mol	Cal/Mol-Kelvin	Cal/Mol-Kelvin
Total	250.590	115.562	217.895

==bold blue 13==

str09fy.high.log

Standard orientation:

Center Number	Atomic Number	Atomic Type	Coordinates (Angstroms) X	Y	Z
1	6	0	-3.685685	-0.115908	1.191356
2	7	0	-2.983200	-0.666538	2.111897
3	6	0	-5.122987	-0.378873	0.985167
4	1	0	-5.501939	-1.177509	1.622724
5	1	0	-5.674107	0.545991	1.188011
6	1	0	-5.281773	-0.626121	-0.065910
7	1	0	-3.191997	0.632881	0.575372
8	8	0	-3.053037	-1.500975	-1.179540
9	1	0	-2.617356	-0.822527	-1.735735
10	1	0	-2.356861	-2.159717	-0.996534
11	1	0	-1.967024	-0.434250	2.240061
12	1	0	-3.393521	-1.364613	2.724889
13	8	0	-0.950600	-3.334088	-0.604635
14	1	0	-0.134401	-2.795490	-0.516060
15	1	0	-0.749873	-4.003587	-1.267601
16	8	0	1.238020	-1.645347	-0.325519
17	1	0	1.125651	-1.099416	0.470744
18	1	0	2.131986	-2.056991	-0.255071
19	6	0	-0.130261	0.062260	2.273659
20	7	0	0.936940	0.445442	1.989232
21	8	0	-2.455696	2.448615	-0.593872
22	1	0	-1.595341	2.739121	-0.209017
23	1	0	-2.965525	3.250868	-0.751137
24	8	0	-1.832497	0.594475	-2.621504
25	1	0	-2.057295	1.312149	-1.997528

26	1	0	-0.856666	0.506672	-2.570149
27	8	0	2.258779	2.480283	-1.121236
28	1	0	2.825717	2.155192	-0.388223
29	1	0	2.783758	3.123033	-1.611876
30	8	0	0.943577	0.260741	-2.398578
31	1	0	1.082523	-0.436974	-1.727106
32	1	0	1.411100	1.039732	-2.045423
33	1	0	2.590873	1.039360	1.548530
34	8	0	3.431528	1.398314	1.167820
35	1	0	3.747529	2.059999	1.795233
36	1	0	5.943504	-0.654030	1.231419
37	8	0	5.289654	-0.575822	0.528108
38	1	0	4.677120	0.137881	0.807011
39	1	0	0.163440	2.283119	1.096008
40	8	0	-0.013309	3.024787	0.494590
41	1	0	0.718287	2.980575	-0.151266
42	8	0	3.749213	-2.719425	-0.207874
43	1	0	4.367518	-2.016126	0.095855
44	1	0	3.887027	-3.468879	0.381704

SCF Done: E(RB3LYP) = -1068.71968574 A.U. after 1 cycles

Zero-point correction= 0.363317 (a.u.)

Thermal correction to Energy= 0.400233

Thermal correction to Enthalpy= 0.401177

Thermal correction to Gibbs Free Energy= 0.292127

Sum of electronic and zero-point Energies= -1068.356369

Sum of electronic and thermal Energies= -1068.319453

Sum of electronic and thermal Enthalpies= -1068.318508

Sum of electronic and thermal Free Energies= -1068.427558

	E (Thermal)	CV	S
	KCal/Mol	Cal/Mol-Kelvin	Cal/Mol-Kelvin
Total	251.150	121.613	229.515

==bold blue 14==

str12bx.high.log

Standard orientation:

Center Number	Atomic Number	Atomic Type	Coordinates (Angstroms) X	Y	Z
1	6	0	3.131131	0.493687	-1.070204
2	7	0	1.885207	0.753617	-1.396867
3	6	0	4.160558	1.579012	-1.099006
4	1	0	5.061378	1.273119	-0.572013
5	1	0	4.413767	1.769823	-2.147827
6	1	0	3.780696	2.504040	-0.664564
7	1	0	3.463870	-0.520524	-1.245681
8	8	0	0.007699	-1.501916	-1.481585
9	1	0	0.456226	-2.372989	-1.514639
10	1	0	-0.716955	-1.475561	-2.140418
11	1	0	1.216751	-0.015147	-1.498587
12	1	0	1.507485	1.700915	-1.290650
13	8	0	-2.279465	-0.877209	-2.913216
14	1	0	-2.715187	-0.437054	-2.153676
15	1	0	-2.921377	-1.508922	-3.255469
16	8	0	-3.161663	0.256255	-0.499329
17	1	0	-2.375621	0.083973	0.040763
18	1	0	-3.334318	1.217838	-0.375382
19	6	0	3.143269	0.024518	1.074946
20	7	0	2.655835	-0.296548	2.086991
21	8	0	1.523147	-3.724267	-0.876503
22	1	0	1.483174	-3.549740	0.091044
23	1	0	1.298300	-4.654063	-0.989630
24	8	0	-1.132261	-1.466181	1.015251
25	1	0	-2.010341	-1.899088	1.013141
26	1	0	-0.772002	-1.518116	0.097612
27	8	0	-0.847620	3.089349	1.383925
28	1	0	-0.572881	2.233543	1.806891
29	1	0	-0.767159	3.765427	2.067044
30	8	0	-3.827117	-2.200804	0.707223

31	1	0	-4.004473	-1.329320	0.309421
32	1	0	-4.445069	-2.289556	1.441503
33	1	0	0.821821	0.496234	2.435431
34	8	0	-0.126598	0.719364	2.466312
35	1	0	-0.563295	-0.028598	2.012057
36	1	0	1.169216	4.178697	-0.966588
37	8	0	0.663990	3.358481	-0.961839
38	1	0	0.166267	3.354581	-0.118093
39	1	0	1.803522	-2.162425	1.946379
40	8	0	1.221641	-2.923933	1.771926
41	1	0	0.335055	-2.523360	1.696713
42	8	0	-3.334046	2.930015	0.105821
43	1	0	-2.478870	3.065591	0.566971
44	1	0	-3.358671	3.584668	-0.600929

SCF Done: E(RB3LYP) = -1068.70807979 A.U. after 1 cycles

	1	2	3
	A	A	A
Frequencies --	-246.1252	25.7612	29.1816

Zero-point correction= 0.364080 (a.u.)

Thermal correction to Energy= 0.400073

Thermal correction to Enthalpy= 0.401018

Thermal correction to Gibbs Free Energy= 0.294551

Sum of electronic and zero-point Energies= -1068.343999

Sum of electronic and thermal Energies= -1068.308006

Sum of electronic and thermal Enthalpies= -1068.307062

Sum of electronic and thermal Free Energies= -1068.413528

	E (Thermal)	CV	S
	KCal/Mol	Cal/Mol-Kelvin	Cal/Mol-Kelvin
Total	251.050	119.576	224.077

==bold bule 15==

str12a.rev.high.log

Standard orientation:

Center Number	Atomic Number	Atomic Type	Coordinates (Angstroms) X	Y	Z
1	6	0	3.912038	-0.908036	-0.439284
2	7	0	3.037518	-0.588071	-1.562660
3	6	0	5.185244	-0.051742	-0.451294
4	1	0	5.838733	-0.305980	0.384965
5	1	0	5.717603	-0.234879	-1.385500
6	1	0	4.935887	1.009676	-0.391935
7	1	0	4.195460	-1.962734	-0.509787
8	8	0	-1.822108	-1.050110	-1.376780
9	1	0	-1.022647	-1.590959	-1.544193
10	1	0	-2.589045	-1.659868	-1.375261
11	1	0	2.219545	-1.195944	-1.570998
12	1	0	2.721407	0.381708	-1.512948
13	8	0	-4.208971	-2.385146	-0.850605
14	1	0	-4.926867	-2.471408	-1.487324
15	1	0	-4.490087	-1.690709	-0.220347
16	8	0	-3.341097	1.356239	-1.104813
17	1	0	-2.674862	0.706024	-1.386748
18	1	0	-2.849961	2.181548	-0.903579
19	6	0	3.211571	-0.770861	0.864838
20	7	0	2.600999	-0.661666	1.839267
21	8	0	0.526695	-2.562290	-1.314407
22	1	0	0.479069	-2.765820	-0.352529
23	1	0	0.599223	-3.410784	-1.766058
24	8	0	-1.667661	-0.723399	1.390111
25	1	0	-2.609610	-0.596830	1.601958
26	1	0	-1.662020	-0.750731	0.406278
27	8	0	0.452289	3.217081	0.738775
28	1	0	0.373123	2.454642	1.371140
29	1	0	0.787420	3.960133	1.254380
30	8	0	-4.420677	-0.178945	0.859418
31	1	0	-4.147548	0.519837	0.214710

32	1	0	-5.191583	0.154866	1.331219
33	1	0	1.020744	0.546999	2.345606
34	8	0	0.215013	1.088729	2.369407
35	1	0	-0.497764	0.493251	2.052395
36	1	0	2.678040	3.070142	-1.658102
37	8	0	2.081311	2.355786	-1.410610
38	1	0	1.554116	2.703143	-0.663950
39	1	0	1.016947	-2.332436	1.827445
40	8	0	0.256120	-2.790638	1.444036
41	1	0	-0.497918	-2.189281	1.602660
42	8	0	-1.990488	3.703705	-0.508952
43	1	0	-1.120523	3.570693	-0.074600
44	1	0	-1.823035	4.289586	-1.255345

SCF Done: E(RB3LYP) = -1068.74245236 A.U. after 1 cycles

Zero-point correction= 0.366652 (a.u.)

Thermal correction to Energy= 0.402427

Thermal correction to Enthalpy= 0.403371

Thermal correction to Gibbs Free Energy= 0.296471

Sum of electronic and zero-point Energies= -1068.375800

Sum of electronic and thermal Energies= -1068.340025

Sum of electronic and thermal Enthalpies= -1068.339081

Sum of electronic and thermal Free Energies= -1068.445981

	E (Thermal)	CV	S
	KCal/Mol	Cal/Mol-Kelvin	Cal/Mol-Kelvin
Total	252.527	119.755	224.991

==bold blue 16==

str09dd.high.log

Standard orientation:

Center Number	Atomic Number	Atomic Type	Coordinates (Angstroms) X	Y	Z

1	6	0	0.655502	-1.467905	-1.872911
2	7	0	0.885341	-0.428736	-0.851769
3	6	0	-0.168850	-2.696239	-1.563592
4	1	0	-1.065429	-2.442326	-1.000257
5	1	0	-0.474132	-3.155073	-2.504192
6	1	0	0.419205	-3.410086	-0.991873
7	1	0	1.161374	-1.313844	-2.803605
8	8	0	2.423274	-2.206848	-1.216129
9	1	0	2.725444	-2.902584	-1.810415
10	1	0	2.489235	-2.360644	0.542396
11	1	0	1.872178	-0.522240	-0.584902
12	1	0	0.737251	0.538405	-1.192886
13	8	0	2.401659	-2.199849	1.516068
14	1	0	0.609523	-1.747708	1.771685
15	1	0	2.773513	-1.306771	1.654307
16	8	0	-0.167739	-1.163650	1.630743
17	1	0	0.311676	-0.604588	0.003665
18	1	0	-1.002520	-1.662984	1.778452
19	6	0	-0.990459	-0.402108	-2.623292
20	7	0	-1.956688	0.223239	-2.797066
21	8	0	3.584290	2.344496	-0.338502
22	1	0	3.945282	3.218963	-0.156345
23	1	0	2.696726	2.495299	-0.720260
24	8	0	3.012577	0.588062	1.811044
25	1	0	3.324557	1.180583	1.099704
26	1	0	2.159074	0.973902	2.096042
27	8	0	-1.010888	2.973414	0.488751
28	1	0	-1.829225	2.569704	0.121879
29	1	0	-1.239070	3.881944	0.719340
30	8	0	0.387890	1.412182	2.528020
31	1	0	0.058910	0.512764	2.329384
32	1	0	-0.079204	1.989346	1.898589
33	1	0	-2.842377	1.223612	-1.498833
34	8	0	-3.192050	1.697991	-0.709772
35	1	0	-3.865003	2.304423	-1.043829
36	1	0	-5.169332	-0.237939	1.000809

37	8	0	-4.239058	-0.093881	1.206086
38	1	0	-3.904290	0.516114	0.517621
39	1	0	0.780864	2.781705	-2.193414
40	8	0	0.947973	2.383699	-1.330716
41	1	0	0.257297	2.736326	-0.722808
42	8	0	-2.666014	-2.223408	1.970804
43	1	0	-3.267463	-1.508114	1.664989
44	1	0	-2.917108	-3.009868	1.474184

SCF Done: E(RB3LYP) = -1068.63394443 A.U. after 1 cycles

	1	2	3
	A	A	A
Frequencies --	-655.8315	24.3090	26.2192

Zero-point correction= 0.366497 (a.u.)

Thermal correction to Energy= 0.401043

Thermal correction to Enthalpy= 0.401988

Thermal correction to Gibbs Free Energy= 0.300051

Sum of electronic and zero-point Energies= -1068.267447

Sum of electronic and thermal Energies= -1068.232901

Sum of electronic and thermal Enthalpies= -1068.231957

Sum of electronic and thermal Free Energies= -1068.333893

	E (Thermal)	CV	S
	KCal/Mol	Cal/Mol-Kelvin	Cal/Mol-Kelvin
Total	251.659	117.137	214.543

==bold blue 17==

Standard orientation:

Center Number	Atomic Number	Atomic Type	Coordinates (Angstroms) X	Y	Z
1	6	0	-0.911994	-3.187801	-0.114544
2	8	0	0.340244	-2.540030	0.109371
3	6	0	-0.886328	-3.739735	-1.533364

4	1	0	-1.821442	-4.259992	-1.751620
5	1	0	-0.764001	-2.927136	-2.254681
6	1	0	-0.061081	-4.445175	-1.654332
7	1	0	-1.024144	-4.009878	0.600687
8	7	0	-2.009452	-2.241074	0.147681
9	1	0	-2.078096	-1.544251	1.785520
10	1	0	-2.002857	-1.491927	-0.551888
11	1	0	-2.898562	-2.728141	0.054554
12	1	0	0.428237	-2.285604	1.049536
13	8	0	0.662335	-1.391125	2.692937
14	1	0	1.058927	-1.841107	3.447571
15	1	0	1.402924	0.151613	1.981892
16	8	0	1.672122	0.901000	1.411387
17	1	0	1.499497	0.580846	0.493964
18	1	0	0.395243	2.316913	1.470335
19	6	0	-0.438320	2.975129	1.219527
20	7	0	-1.333249	3.622072	0.899559
21	1	0	0.884391	-1.054882	-0.808217
22	8	0	1.059578	-0.129019	-1.078364
23	1	0	1.836161	-0.138325	-1.682076
24	8	0	4.346888	1.532798	1.434251
25	1	0	3.383971	1.330904	1.486054
26	1	0	4.446503	2.436921	1.751759
27	8	0	3.329092	-0.074661	-2.633863
28	1	0	3.314278	0.415029	-3.463265
29	1	0	4.072830	0.300066	-2.108377
30	8	0	-3.021030	2.715236	-1.345223
31	1	0	-3.013896	3.325873	-2.092502
32	1	0	-2.559721	3.178385	-0.623738
33	8	0	5.331134	0.953547	-1.061669
34	1	0	5.015640	1.171924	-0.154505
35	1	0	6.114421	0.405013	-0.944861
36	8	0	-2.009958	-1.220874	2.731125
37	1	0	-0.299685	-1.270908	2.896087
38	1	0	-2.436746	-0.357332	2.755288
39	8	0	-1.668427	0.171028	-1.835384

40	1	0	-2.064467	1.044708	-1.677633
41	1	0	-0.714371	0.261143	-1.655252
42	8	0	-5.595327	1.700650	-0.657098
43	1	0	-4.731423	2.092160	-0.882746
44	1	0	-6.136980	2.442211	-0.367697

SCF Done: E(RB3LYP) = -1068.72221025 A.U. after 1 cycles

Zero-point correction= 0.363872 (a.u.)
Thermal correction to Energy= 0.400493
Thermal correction to Enthalpy= 0.401437
Thermal correction to Gibbs Free Energy= 0.287451
Sum of electronic and zero-point Energies= -1068.358338
Sum of electronic and thermal Energies= -1068.321717
Sum of electronic and thermal Enthalpies= -1068.320773
Sum of electronic and thermal Free Energies= -1068.434759

	E (Thermal)	CV	S
	KCal/Mol	Cal/Mol-Kelvin	Cal/Mol-Kelvin
Total	251.313	119.180	239.905

==bold blue 18==
str27a.high.log
Standard orientation:

Center Number	Atomic Number	Atomic Type	Coordinates (Angstroms) X	Y	Z
1	6	0	-1.610572	-2.404070	0.731466
2	8	0	-0.220730	-2.170365	0.762927
3	6	0	-1.915128	-3.462896	-0.310697
4	1	0	-2.988653	-3.654627	-0.363904
5	1	0	-1.563344	-3.143544	-1.294503
6	1	0	-1.411159	-4.392346	-0.041619
7	1	0	-1.966340	-2.716166	1.717592
8	7	0	-2.305255	-1.110863	0.424709

9	1	0	-2.153376	-0.376903	1.274326
10	1	0	-1.948682	-0.722287	-0.468135
11	1	0	-3.314928	-1.216462	0.299251
12	1	0	0.026102	-1.648255	1.568424
13	8	0	0.402285	-0.426972	2.742231
14	1	0	0.626223	-0.670022	3.646690
15	1	0	1.419935	0.622068	1.748862
16	8	0	1.830909	1.143547	1.017765
17	1	0	1.677289	0.588392	0.219484
18	1	0	0.611569	2.564898	0.665077
19	6	0	-0.244166	3.147752	0.315964
20	7	0	-1.171395	3.699679	-0.081304
21	1	0	0.811326	-1.277241	-0.587264
22	8	0	1.213626	-0.551238	-1.101132
23	1	0	2.016363	-0.909471	-1.544270
24	8	0	4.557003	1.397919	1.120423
25	1	0	3.571645	1.352665	1.130043
26	1	0	4.780081	2.334345	1.082882
27	8	0	3.552865	-1.434197	-2.228407
28	1	0	3.696473	-1.285253	-3.169230
29	1	0	4.312714	-1.011590	-1.765544
30	8	0	-3.014070	2.268145	-1.889977
31	1	0	-3.347694	2.762266	-2.648466
32	1	0	-2.567745	2.919927	-1.321597
33	8	0	5.584761	-0.214413	-0.847161
34	1	0	5.251268	0.372449	-0.129429
35	1	0	6.241749	-0.787454	-0.437236
36	8	0	-1.900808	0.378042	2.474654
37	1	0	-0.592115	0.000583	2.724216
38	1	0	-2.001101	1.327729	2.355251
39	8	0	-1.363690	-0.067492	-2.115181
40	1	0	-1.733710	0.830378	-2.166325
41	1	0	-0.403734	0.004078	-1.960899
42	8	0	-4.690421	0.261479	-0.680542
43	1	0	-4.205651	1.009859	-1.074945
44	1	0	-5.502599	0.639922	-0.327782

SCF Done: E(RB3LYP) = -1068.71089585 A.U. after 1 cycles

	1	2	3
	A	A	A
Frequencies --	-164.8109	17.0253	21.8186

Zero-point correction= 0.361475 (a.u.)

Thermal correction to Energy= 0.395942

Thermal correction to Enthalpy= 0.396886

Thermal correction to Gibbs Free Energy= 0.292038

Sum of electronic and zero-point Energies= -1068.349421

Sum of electronic and thermal Energies= -1068.314954

Sum of electronic and thermal Enthalpies= -1068.314009

Sum of electronic and thermal Free Energies= -1068.418858

	E (Thermal)	CV	S
	KCal/Mol	Cal/Mol-Kelvin	Cal/Mol-Kelvin
Total	248.458	114.447	220.672

[2] The second reaction in Scheme 8 with stoichiometry C3H29N2O11+.
Each species is specified by orange bold numbers. Odd numbers are
for precursor, intermediates and product. Even numbers are
for transition states. They are shown in Figures 5 and S3
(the most likely route, 1 -> 14 -> 15 -> 16B -> 17 ->
18 -> 19 -> 20B -> 7 -> 8 -> 9 -> 10 -> 11 -> 12 -> 13).
Species of less favorable routes are shown in Figures S4 and S5.

==bold orange 1==

str06dc.for.high.log

Standard orientation:

Center	Atomic	Atomic	Coordinates (Angstroms)		
Number	Number	Type	X	Y	Z

1	6	0	-4.441000	2.545055	0.419683
2	7	0	-5.676300	2.269110	1.150280
3	6	0	-4.735050	3.154803	-0.956986
4	1	0	-5.295791	4.078826	-0.813175
5	1	0	-3.810656	3.379440	-1.491077
6	1	0	-5.331951	2.470047	-1.563317
7	1	0	-3.853162	3.256199	1.006804
8	6	0	-3.589767	1.337771	0.272383
9	7	0	-2.959063	0.376150	0.178553
10	1	0	-5.487293	1.921805	2.085173
11	1	0	-1.262932	-0.252286	-0.794689
12	1	0	-6.239933	1.574603	0.668710
13	8	0	-0.478965	-0.660983	-1.199176
14	1	0	0.824242	0.020353	-0.897406
15	1	0	-0.487218	-1.600762	-0.896398
16	8	0	-0.596829	-3.188685	-0.138739
17	1	0	-1.420963	-3.079274	0.384750
18	1	0	-0.739832	-3.940997	-0.751630
19	8	0	-2.908057	-2.472957	1.178354
20	1	0	-2.988074	-2.521935	2.137839
21	1	0	-2.996898	-1.531806	0.953155
22	8	0	-0.873395	-5.302616	-1.911286
23	1	0	-1.554622	-5.250395	-2.592258
24	1	0	-0.942045	-6.194396	-1.549980
25	8	0	3.169329	2.735791	1.129913
26	1	0	2.357921	2.309520	0.823984
27	1	0	3.614117	2.057320	1.671446
28	8	0	4.343310	0.546213	2.574026
29	1	0	5.308208	0.520724	2.613201
30	1	0	4.047273	0.457419	3.489270
31	8	0	1.732374	0.455562	-0.669478
32	1	0	2.205512	0.816831	-1.496662
33	1	0	2.344036	-0.222405	-0.150295
34	8	0	3.060168	1.446743	-2.635615
35	1	0	2.581151	1.799324	-3.394273
36	1	0	3.587797	2.192021	-2.253943

37	8	0	3.194735	-1.123244	0.603144
38	1	0	3.598324	-0.673909	1.370370
39	1	0	2.708797	-1.921056	0.927820
40	8	0	4.377943	3.375275	-1.274995
41	1	0	4.059178	3.199574	-0.361286
42	1	0	5.340493	3.356631	-1.234638
43	8	0	1.715058	-3.251900	1.369642
44	1	0	0.886502	-3.304924	0.841904
45	1	0	2.104908	-4.132678	1.347964

SCF Done: E(RB3LYP) = -1069.17455066 A.U. after 1 cycles

Zero-point correction= 0.374954 (a.u.)
Thermal correction to Energy= 0.412138
Thermal correction to Enthalpy= 0.413082
Thermal correction to Gibbs Free Energy= 0.297078
Sum of electronic and zero-point Energies= -1068.799597
Sum of electronic and thermal Energies= -1068.762413
Sum of electronic and thermal Enthalpies= -1068.761469
Sum of electronic and thermal Free Energies= -1068.877473

	E (Thermal)	CV	S
	KCal/Mol	Cal/Mol-Kelvin	Cal/Mol-Kelvin
Total	258.620	120.933	244.151

==bold orange 2==
str06dc.high.log

Standard orientation:

Center Number	Atomic Number	Atomic Type	Coordinates (Angstroms) X	Y	Z
1	6	0	-0.243131	3.025723	-0.020641
2	7	0	0.415673	3.994518	-0.875111
3	6	0	-0.000100	3.346238	1.461514
4	1	0	-0.398242	4.340202	1.665818

5	1	0	-0.491824	2.627095	2.115706
6	1	0	1.069609	3.342797	1.682131
7	1	0	-1.313873	3.058436	-0.230866
8	6	0	0.206736	1.617462	-0.324672
9	7	0	1.057766	0.985144	-0.855523
10	1	0	0.246808	3.817046	-1.859619
11	1	0	1.279500	-0.035546	-0.994443
12	1	0	1.418478	4.019796	-0.720880
13	8	0	1.808068	-1.602883	-1.241664
14	1	0	1.612348	-1.913666	-2.134659
15	1	0	2.804868	-1.576650	-1.168054
16	8	0	4.436206	-1.302576	-1.075852
17	1	0	4.534410	-0.330430	-1.087217
18	1	0	4.920258	-1.630182	-0.292474
19	8	0	4.296078	1.499021	-1.164210
20	1	0	4.711519	1.999694	-1.876159
21	1	0	3.351204	1.692114	-1.232226
22	8	0	5.801227	-2.354617	1.135381
23	1	0	5.632874	-1.971585	2.004501
24	1	0	6.761043	-2.426588	1.072374
25	8	0	-1.099216	0.560119	0.539189
26	1	0	-1.334499	-0.256558	-0.043026
27	1	0	-1.966767	0.982425	0.751393
28	8	0	-3.682536	1.333298	1.115429
29	1	0	-3.868482	1.489036	2.050784
30	1	0	-4.098390	2.067911	0.645262
31	8	0	-1.789471	-1.536934	-0.762669
32	1	0	-1.263903	-2.248572	-0.332749
33	1	0	-2.732004	-1.645452	-0.498252
34	8	0	0.113593	-3.069670	0.522685
35	1	0	0.827077	-2.694506	-0.026405
36	1	0	0.145220	-2.550090	1.349479
37	8	0	-4.359171	-1.373129	0.146397
38	1	0	-4.349262	-0.459312	0.473914
39	1	0	-5.147878	-1.458108	-0.424043
40	8	0	0.100342	-1.154792	2.608096

41	1	0	-0.348033	-0.447651	2.114628
42	1	0	-0.381714	-1.241313	3.438189
43	8	0	-6.620181	-1.651318	-1.494106
44	1	0	-6.468369	-1.822402	-2.431172
45	1	0	-7.286599	-2.294266	-1.224112

SCF Done: E(RB3LYP) = -1069.12306714 A.U. after 3 cycles

	1	2	3
	A	A	A
Frequencies --	-275.2153	11.0161	14.0413

Zero-point correction= 0.374468 (a.u.)

Thermal correction to Energy= 0.411558

Thermal correction to Enthalpy= 0.412502

Thermal correction to Gibbs Free Energy= 0.299975

Sum of electronic and zero-point Energies= -1068.748600

Sum of electronic and thermal Energies= -1068.711509

Sum of electronic and thermal Enthalpies= -1068.710565

Sum of electronic and thermal Free Energies= -1068.823092

	E (Thermal)	CV	S
	KCal/Mol	Cal/Mol-Kelvin	Cal/Mol-Kelvin
Total	258.256	121.103	236.832

==bold orange 3==

str14bg.rev.higha

Standard orientation:

Center Number	Atomic Number	Atomic Type	Coordinates (Angstroms) X	Y	Z
1	6	0	-2.924351	-1.642301	-0.624548
2	7	0	-4.060295	-1.069488	-1.363656
3	6	0	-3.435533	-2.518477	0.528223
4	1	0	-4.024745	-3.348222	0.130676

5	1	0	-2.601537	-2.934107	1.094348
6	1	0	-4.065920	-1.935437	1.203752
7	1	0	-2.294826	-2.262461	-1.274481
8	6	0	-2.026146	-0.536081	-0.088053
9	7	0	-2.316630	0.695123	0.047405
10	1	0	-3.743446	-0.662086	-2.240601
11	1	0	-4.698687	-1.818658	-1.618310
12	1	0	-5.263391	0.169952	-0.579482
13	8	0	-0.821705	-1.022345	0.271736
14	1	0	-0.251786	-0.335381	0.709034
15	1	0	0.316089	-2.306349	-0.558549
16	8	0	1.041304	-2.854530	-0.909013
17	1	0	0.693230	-3.282560	-1.699553
18	1	0	2.513249	-1.977255	-1.075328
19	8	0	3.322297	-1.411284	-1.125437
20	1	0	3.168544	-0.224132	-0.496425
21	1	0	4.091265	-1.942138	-0.815652
22	8	0	3.048401	0.736236	0.008718
23	1	0	3.038184	1.491759	-0.634149
24	1	0	2.205312	0.753569	0.585647
25	8	0	-5.825390	0.906424	-0.246345
26	1	0	-3.274517	0.873401	-0.250336
27	1	0	-6.170399	0.599862	0.598990
28	8	0	3.031082	2.818303	-1.720589
29	1	0	3.806488	3.385564	-1.610311
30	1	0	3.044045	2.542303	-2.647308
31	8	0	5.533621	-2.787158	-0.299479
32	1	0	5.516972	-3.216828	0.564123
33	1	0	5.905278	-3.440438	-0.904314
34	8	0	0.914049	0.730628	1.441172
35	1	0	0.411872	1.594903	1.370283
36	1	0	1.077821	0.546678	2.394777
37	8	0	-0.656669	2.786414	0.971935
38	1	0	-1.318305	2.161381	0.589536
39	1	0	-0.298209	3.312957	0.228815
40	8	0	1.435483	0.119226	4.062926

41	1	0	1.903061	0.771160	4.598732
42	1	0	0.700730	-0.182685	4.610459
43	8	0	0.538159	4.172056	-1.143963
44	1	0	1.408890	3.790501	-1.354773
45	1	0	0.674337	5.124700	-1.092863

SCF Done: E(RB3LYP) = -1069.17722194 A.U. after 1 cycles

Zero-point correction= 0.377929 (a.u.)
Thermal correction to Energy= 0.413606
Thermal correction to Enthalpy= 0.414551
Thermal correction to Gibbs Free Energy= 0.304302
Sum of electronic and zero-point Energies= -1068.799293
Sum of electronic and thermal Energies= -1068.763616
Sum of electronic and thermal Enthalpies= -1068.762671
Sum of electronic and thermal Free Energies= -1068.872919

	E (Thermal)	CV	S
	KCal/Mol	Cal/Mol-Kelvin	Cal/Mol-Kelvin
Total	259.542	116.175	232.037

==bold orange 4==

str14bg.higha.log

Standard orientation:

Center Number	Atomic Number	Atomic Type	Coordinates (Angstroms) X	Y	Z
1	6	0	-3.071801	-1.675139	-0.420937
2	7	0	-4.138289	-1.146845	-1.285482
3	6	0	-3.680398	-2.315784	0.835317
4	1	0	-4.319035	-3.154899	0.549910
5	1	0	-2.896949	-2.693198	1.493103
6	1	0	-4.283578	-1.588481	1.383620
7	1	0	-2.473213	-2.434574	-0.938385
8	6	0	-2.108784	-0.564812	-0.026588

9	7	0	-2.313105	0.688922	-0.058854
10	1	0	-3.768713	-0.921637	-2.206447
11	1	0	-4.833309	-1.873998	-1.433030
12	1	0	-5.217218	0.324197	-0.777022
13	8	0	-0.939214	-1.077598	0.417514
14	1	0	-0.346462	-0.377326	0.764558
15	1	0	0.193043	-2.463809	-0.358228
16	8	0	0.899057	-3.046233	-0.685819
17	1	0	0.520396	-3.518137	-1.436194
18	1	0	2.449224	-2.164017	-0.964215
19	8	0	3.250719	-1.610471	-1.071713
20	1	0	3.140239	-0.189491	-0.323421
21	1	0	4.018765	-2.168240	-0.838033
22	8	0	3.041320	0.687569	0.158854
23	1	0	3.162138	1.417054	-0.484716
24	1	0	1.946152	0.778473	0.821978
25	8	0	-5.658960	1.183572	-0.587819
26	1	0	-3.253310	0.900896	-0.390145
27	1	0	-6.120404	1.054817	0.247754
28	8	0	3.320864	2.846811	-1.608736
29	1	0	4.027635	3.446276	-1.334268
30	1	0	3.544861	2.585837	-2.512027
31	8	0	5.535183	-3.103329	-0.444703
32	1	0	5.586885	-3.530055	0.418775
33	1	0	5.816429	-3.775599	-1.076629
34	8	0	0.974392	0.842772	1.409060
35	1	0	0.412884	1.696417	1.139804
36	1	0	1.137523	0.822404	2.393187
37	8	0	-0.517568	2.683249	0.661768
38	1	0	-1.235494	2.085968	0.322607
39	1	0	-0.144861	3.181273	-0.101235
40	8	0	1.453881	0.719733	4.018506
41	1	0	1.630783	1.544843	4.486764
42	1	0	0.815432	0.242016	4.562162
43	8	0	0.698169	3.964708	-1.434136
44	1	0	1.620358	3.659277	-1.526217

45	1	0	0.732720	4.927330	-1.462622

SCF Done: E(RB3LYP) = -1069.17424941 A.U. after 1 cycles

	1	2	3
	A	A	A
Frequencies --	-254.3719	13.6836	19.2992

Zero-point correction= 0.375716 (a.u.)
Thermal correction to Energy= 0.410788
Thermal correction to Enthalpy= 0.411732
Thermal correction to Gibbs Free Energy= 0.303602
Sum of electronic and zero-point Energies= -1068.798534
Sum of electronic and thermal Energies= -1068.763462
Sum of electronic and thermal Enthalpies= -1068.762518
Sum of electronic and thermal Free Energies= -1068.870647

	E (Thermal)	CV	S
	KCal/Mol	Cal/Mol-Kelvin	Cal/Mol-Kelvin
Total	257.773	114.814	227.578

==bold orange 5==

str14bg.for.higha.log

Standard orientation:

Center Number	Atomic Number	Atomic Type	Coordinates (Angstroms) X	Y	Z
1	6	0	-2.755851	-1.836958	-0.667535
2	7	0	-3.874184	-1.373406	-1.499356
3	6	0	-3.272457	-2.714838	0.484734
4	1	0	-3.745787	-3.607417	0.070818
5	1	0	-2.451334	-3.032253	1.127857
6	1	0	-4.007251	-2.173196	1.083879
7	1	0	-2.032570	-2.421186	-1.246648
8	6	0	-1.959202	-0.679106	-0.090360

9	7	0	-2.473435	0.500134	0.087861
10	1	0	-3.533838	-1.036543	-2.397023
11	1	0	-4.478639	-2.164182	-1.705143
12	1	0	-5.076937	-0.019374	-0.908638
13	8	0	-0.741497	-0.983287	0.231845
14	1	0	-0.160092	-0.280259	0.780435
15	1	0	0.556384	-2.338412	-0.513751
16	8	0	1.313201	-2.855544	-0.830740
17	1	0	0.974497	-3.388237	-1.559415
18	1	0	2.801749	-1.817960	-1.151465
19	8	0	3.532312	-1.179984	-1.270540
20	1	0	3.189444	0.274175	-0.380086
21	1	0	4.355189	-1.652093	-1.042150
22	8	0	2.922697	1.049821	0.168899
23	1	0	2.859000	1.815552	-0.428673
24	1	0	1.573177	0.738460	1.025558
25	8	0	-5.313492	0.868409	-0.557156
26	1	0	-3.459207	0.667994	-0.139604
27	1	0	-5.969073	0.718443	0.133213
28	8	0	2.657180	3.397765	-1.498360
29	1	0	3.358994	4.034766	-1.310655
30	1	0	2.737886	3.204367	-2.441548
31	8	0	5.986266	-2.425619	-0.639855
32	1	0	6.088260	-2.823387	0.232900
33	1	0	6.334186	-3.079728	-1.257402
34	8	0	0.718210	0.558999	1.520127
35	1	0	-0.309420	2.172685	1.452574
36	1	0	0.944120	0.214618	2.414824
37	8	0	-1.068679	2.684775	1.123291
38	1	0	-1.934042	1.305565	0.467319
39	1	0	-0.697458	3.297938	0.453934
40	8	0	1.346518	-0.387454	4.013223
41	1	0	1.379492	0.260504	4.727217
42	1	0	0.831400	-1.125697	4.360267
43	8	0	0.046843	4.323954	-0.831573
44	1	0	0.956769	4.061412	-1.069344

45	1	0	0.077001	5.277071	-0.693103

SCF Done: E(RB3LYP) = -1069.19573751 A.U. after 1 cycles

Zero-point correction= 0.378020 (a.u.)

Thermal correction to Energy= 0.414620

Thermal correction to Enthalpy= 0.415564

Thermal correction to Gibbs Free Energy= 0.302978

Sum of electronic and zero-point Energies= -1068.817717

Sum of electronic and thermal Energies= -1068.781118

Sum of electronic and thermal Enthalpies= -1068.780173

Sum of electronic and thermal Free Energies= -1068.892759

	E (Thermal)	CV	S
	KCal/Mol	Cal/Mol-Kelvin	Cal/Mol-Kelvin
Total	260.178	119.108	236.957

==bold orange 6==

str18ccc.high.log

Standard orientation:

Center Number	Atomic Number	Atomic Type	Coordinates (Angstroms) X	Y	Z
1	6	0	-3.545171	0.921914	0.701128
2	7	0	-4.435047	0.097444	1.532170
3	6	0	-4.368138	1.767620	-0.283962
4	1	0	-5.008209	2.453237	0.275216
5	1	0	-3.713875	2.359512	-0.924737
6	1	0	-4.997794	1.130728	-0.908773
7	1	0	-2.934477	1.596885	1.309964
8	6	0	-2.544822	0.081293	-0.081020
9	7	0	-2.808827	-1.159398	-0.401760
10	1	0	-3.933487	-0.257574	2.343147
11	1	0	-5.179090	0.686094	1.897291
12	1	0	-5.346082	-1.410619	0.837080

13	8	0	-1.467066	0.683471	-0.419781
14	1	0	-0.661079	0.201596	-1.154875
15	1	0	-0.702479	2.394123	0.143701
16	8	0	-0.144131	3.171345	0.315711
17	1	0	-0.548721	3.625036	1.063608
18	1	0	1.630122	2.742751	0.423286
19	8	0	2.540851	2.385553	0.423850
20	1	0	2.563403	0.929540	-0.449231
21	1	0	3.126045	3.100438	0.108408
22	8	0	2.481977	0.096669	-0.981774
23	1	0	2.826419	-0.659313	-0.440864
24	1	0	1.207994	-0.077362	-1.486805
25	8	0	-5.435221	-2.278461	0.380745
26	1	0	-3.704558	-1.582784	-0.147877
27	1	0	-6.186277	-2.191555	-0.216768
28	8	0	3.370091	-2.019347	0.413675
29	1	0	4.220479	-2.409328	0.134348
30	1	0	3.397119	-1.955930	1.387523
31	8	0	4.345364	4.353606	-0.471869
32	1	0	4.164246	4.810281	-1.301895
33	1	0	4.604333	5.046910	0.146626
34	8	0	0.217211	-0.212628	-1.892376
35	1	0	-0.293423	-2.127468	-2.073703
36	1	0	0.175328	0.224575	-2.753736
37	8	0	-1.003681	-2.778449	-1.952464
38	1	0	-2.150737	-1.740926	-0.937827
39	1	0	-0.585288	-3.564201	-1.582580
40	8	0	5.809112	-3.148169	-0.426827
41	1	0	5.775198	-4.018162	-0.841833
42	1	0	6.369324	-2.614736	-1.002970
43	8	0	3.426385	-1.833914	3.222933
44	1	0	3.478086	-0.951871	3.609720
45	1	0	2.736259	-2.288548	3.720237

SCF Done: E(RB3LYP) = -1069.18689152   A.U. after   1 cycles

1	2	3
A	A	A

Frequencies -- -701.6425 7.3516 8.4491

Zero-point correction= 0.372481 (a.u.)

Thermal correction to Energy= 0.409426

Thermal correction to Enthalpy= 0.410370

Thermal correction to Gibbs Free Energy= 0.294083

Sum of electronic and zero-point Energies= -1068.814410

Sum of electronic and thermal Energies= -1068.777466

Sum of electronic and thermal Enthalpies= -1068.776522

Sum of electronic and thermal Free Energies= -1068.892808

	E (Thermal)	CV	S
	KCal/Mol	Cal/Mol-Kelvin	Cal/Mol-Kelvin
Total	256.918	118.645	244.746

==bold orange 7==

str18ccc.revhigh.log

Standard orientation:

Center Number	Atomic Number	Atomic Type	Coordinates (Angstroms) X	Y	Z
1	6	0	-3.599832	0.933739	0.696947
2	7	0	-4.558639	0.132876	1.478945
3	6	0	-4.351287	1.811119	-0.314699
4	1	0	-5.007706	2.505567	0.215081
5	1	0	-3.649527	2.395276	-0.911318
6	1	0	-4.960149	1.198130	-0.983250
7	1	0	-3.003881	1.586243	1.344048
8	6	0	-2.576983	0.069468	-0.044673
9	7	0	-2.902017	-1.171964	-0.382234
10	1	0	-4.096599	-0.282104	2.285047
11	1	0	-5.272311	0.752109	1.854931
12	1	0	-5.531573	-1.268916	0.707555

13	8	0	-1.470693	0.583810	-0.337843
14	1	0	-0.313160	0.030237	-1.439218
15	1	0	-0.666524	2.070379	0.276557
16	8	0	-0.087926	2.825791	0.518160
17	1	0	-0.434786	3.178063	1.345073
18	1	0	1.617662	2.481180	0.456440
19	8	0	2.561492	2.206842	0.370582
20	1	0	2.694707	0.987752	-0.399963
21	1	0	3.067755	2.975088	0.030471
22	8	0	2.758007	0.101701	-0.959216
23	1	0	3.075168	-0.705322	-0.384420
24	1	0	1.865798	-0.086321	-1.383148
25	8	0	-5.740254	-2.119364	0.253380
26	1	0	-3.824265	-1.549996	-0.183096
27	1	0	-6.432205	-1.915363	-0.385161
28	8	0	3.517986	-1.878922	0.381116
29	1	0	4.423528	-2.206945	0.195288
30	1	0	3.411831	-1.831250	1.355028
31	8	0	4.100430	4.297677	-0.578407
32	1	0	3.809888	4.768386	-1.368741
33	1	0	4.357837	4.984998	0.047620
34	8	0	0.401430	-0.324615	-2.033401
35	1	0	-0.300791	-2.102799	-2.076331
36	1	0	0.298808	0.118312	-2.885219
37	8	0	-0.978197	-2.789411	-1.936476
38	1	0	-2.240712	-1.759350	-0.896523
39	1	0	-0.514844	-3.544111	-1.556651
40	8	0	6.054044	-2.836192	-0.151897
41	1	0	6.123524	-3.589640	-0.750204
42	1	0	6.724761	-2.210284	-0.450014
43	8	0	3.198839	-1.754510	3.123277
44	1	0	3.208878	-0.882857	3.536320
45	1	0	2.440838	-2.211251	3.507179

SCF Done: E(RB3LYP) = -1069.19912593   A.U. after   1 cycles

Zero-point correction= 0.376676 (a.u.)

Thermal correction to Energy= 0.413710

Thermal correction to Enthalpy= 0.414654

Thermal correction to Gibbs Free Energy= 0.296176

Sum of electronic and zero-point Energies= -1068.822450

Sum of electronic and thermal Energies= -1068.785416

Sum of electronic and thermal Enthalpies= -1068.784472

Sum of electronic and thermal Free Energies= -1068.902950

	E (Thermal)	CV	S
	KCal/Mol	Cal/Mol-Kelvin	Cal/Mol-Kelvin
Total	259.607	118.479	249.358

==bold orange 8==

str18n.high.log

Standard orientation:

Center Number	Atomic Number	Atomic Type	Coordinates (Angstroms) X	Y	Z
1	6	0	-3.154775	-0.773024	0.093084
2	7	0	-2.164860	-0.845918	-0.992148
3	6	0	-4.327905	0.138137	-0.312061
4	1	0	-4.804455	-0.271802	-1.204473
5	1	0	-5.069940	0.184937	0.485639
6	1	0	-3.985362	1.150368	-0.538421
7	1	0	-3.561474	-1.758660	0.346241
8	6	0	-2.570385	-0.254671	1.404860
9	7	0	-1.376907	0.560855	1.344746
10	1	0	-1.485047	-1.584899	-0.809411
11	1	0	-2.644301	-1.107902	-1.856756
12	1	0	-1.347521	0.684007	-1.442671
13	8	0	-3.058831	-0.504368	2.477194
14	1	0	-0.367474	-0.136262	1.406680
15	8	0	0.702567	-0.848945	1.493442
16	1	0	0.612975	-1.640569	0.916552

17	1	0	-1.541307	2.114371	4.428695
18	1	0	1.519284	-0.330985	1.191419
19	8	0	-1.263949	2.409018	3.552978
20	1	0	-1.337248	1.191270	2.166715
21	1	0	-0.448950	2.904580	3.697218
22	8	0	0.134745	-2.939785	-0.200225
23	1	0	-0.103417	-3.789176	0.192149
24	1	0	0.751579	-3.148959	-0.912925
25	8	0	-3.465888	-1.663009	-3.644420
26	1	0	-4.090261	-2.396712	-3.685515
27	1	0	-3.826007	-0.997685	-4.242398
28	8	0	2.726620	0.574872	0.720525
29	1	0	2.516154	1.014953	-0.135987
30	1	0	3.611829	0.162909	0.631683
31	8	0	1.913955	1.730924	-1.619145
32	1	0	0.936495	1.710942	-1.607908
33	1	0	2.169084	2.654631	-1.801302
34	8	0	5.248551	-0.543745	0.487801
35	1	0	5.898289	-0.303140	1.157088
36	1	0	5.350929	-1.507269	0.347873
37	8	0	-0.884509	1.567912	-1.400580
38	1	0	-1.284615	1.101070	0.472606
39	1	0	-1.421507	2.188840	-1.906229
40	8	0	2.679602	4.400616	-2.165254
41	1	0	3.088650	4.904088	-1.451483
42	1	0	3.244049	4.547800	-2.933388
43	8	0	5.541105	-3.280660	0.045067
44	1	0	4.862939	-3.871059	0.394226
45	1	0	5.675646	-3.559533	-0.868484

SCF Done: E(RB3LYP) = -1069.17142236   A.U. after   1 cycles

	1	2	3
	A	A	A
Frequencies --	-740.3525	8.6260	9.6051

Zero-point correction= 0.372009 (a.u.)
Thermal correction to Energy= 0.410476
Thermal correction to Enthalpy= 0.411420
Thermal correction to Gibbs Free Energy= 0.292001
Sum of electronic and zero-point Energies= -1068.799413
Sum of electronic and thermal Energies= -1068.760946
Sum of electronic and thermal Enthalpies= -1068.760002
Sum of electronic and thermal Free Energies= -1068.879422

	E (Thermal)	CV	S
	KCal/Mol	Cal/Mol-Kelvin	Cal/Mol-Kelvin
Total	257.578	121.084	251.340

==bold orange 9==
str18bb.revhigh.log
Standard orientation:

Center Number	Atomic Number	Atomic Type	Coordinates (Angstroms) X	Y	Z
1	6	0	-0.594132	-0.463732	1.956317
2	7	0	0.840399	-0.728805	1.796103
3	6	0	-1.239814	-1.519511	2.875379
4	1	0	-0.772951	-1.459179	3.860436
5	1	0	-2.307037	-1.330677	2.990351
6	1	0	-1.094693	-2.528200	2.483421
7	1	0	-0.785498	0.525185	2.389319
8	6	0	-1.350299	-0.444879	0.634211
9	7	0	-0.730787	-1.036950	-0.544070
10	1	0	1.329241	0.069238	1.378655
11	1	0	1.247738	-0.864378	2.717655
12	1	0	1.356317	-2.205821	0.912777
13	8	0	-2.440270	0.060196	0.519197
14	1	0	-1.572012	3.062190	-2.689120
15	8	0	1.463405	-2.868811	0.176074
16	1	0	-0.052917	-1.788239	-0.329151

17	1	0	1.275591	-3.737863	0.550989
18	1	0	-1.459711	-1.396474	-1.212242
19	8	0	-0.639880	2.926036	-2.896524
20	1	0	-0.156904	-0.240754	-1.087924
21	1	0	-0.573376	3.028320	-3.853494
22	8	0	-2.604377	-1.926154	-2.400877
23	1	0	-2.856304	-2.856541	-2.406800
24	1	0	-3.426592	-1.415571	-2.236806
25	1	0	-3.456246	1.252643	1.786555
26	8	0	-3.966058	1.902374	2.292856
27	1	0	-4.172702	1.460095	3.123313
28	8	0	0.667830	0.770709	-1.763438
29	1	0	0.191836	1.533578	-2.156793
30	1	0	1.329959	1.119440	-1.115019
31	8	0	2.400093	1.375406	0.203849
32	1	0	2.549690	2.286271	0.525879
33	1	0	3.282419	0.981282	0.019347
34	8	0	4.762411	0.070097	-0.350657
35	1	0	4.541025	-0.863114	-0.571016
36	1	0	5.279981	0.400110	-1.093283
37	8	0	2.803269	3.981019	1.156648
38	1	0	2.591656	4.718485	0.572040
39	1	0	2.404541	4.207455	2.005260
40	8	0	3.996746	-2.511302	-0.922394
41	1	0	4.575272	-3.225765	-0.633801
42	1	0	3.115768	-2.706820	-0.536583
43	1	0	-4.023873	0.117733	-0.824118
44	8	0	-4.584137	-0.245317	-1.526457
45	1	0	-4.894262	0.512357	-2.035739

SCF Done: E(RB3LYP) = -1069.17616250 A.U. after 1 cycles

Zero-point correction= 0.376445 (a.u.)

Thermal correction to Energy= 0.413974

Thermal correction to Enthalpy= 0.414918

Thermal correction to Gibbs Free Energy= 0.299788

Sum of electronic and zero-point Energies= -1068.799718

Sum of electronic and thermal Energies= -1068.762188

Sum of electronic and thermal Enthalpies= -1068.761244

Sum of electronic and thermal Free Energies= -1068.876375

	E (Thermal)	CV	S
	KCal/Mol	Cal/Mol-Kelvin	Cal/Mol-Kelvin
Total	259.773	120.612	242.313

==bold orange 10==

str18bb.high.log

Standard orientation:

Center Number	Atomic Number	Atomic Type	Coordinates (Angstroms) X	Y	Z
1	6	0	-0.572996	-0.064176	1.467346
2	7	0	0.632261	-0.849809	1.810247
3	6	0	-1.782659	-0.601667	2.234996
4	1	0	-1.626453	-0.451240	3.305433
5	1	0	-2.692363	-0.075489	1.948327
6	1	0	-1.923959	-1.671691	2.064006
7	1	0	-0.438840	0.989988	1.733185
8	6	0	-0.873430	-0.015332	-0.053946
9	7	0	-1.026735	-1.436366	-0.656894
10	1	0	1.464959	-0.270320	1.720685
11	1	0	0.579227	-1.118882	2.788994
12	1	0	1.073786	-2.340991	0.963583
13	8	0	-1.814666	0.758964	-0.431259
14	1	0	-1.381958	1.821372	-2.221254
15	8	0	1.214303	-3.040550	0.262421
16	1	0	-0.250338	-2.079729	-0.419246
17	1	0	1.007333	-3.891820	0.666847
18	1	0	-1.938985	-1.865342	-0.380612
19	8	0	-0.501864	2.028149	-2.577365
20	1	0	-1.048681	-1.335726	-1.672619

21	1	0	-0.528340	1.812372	-3.517528
22	8	0	-3.610583	-2.494789	-0.326781
23	1	0	-3.980515	-2.927814	0.450372
24	1	0	-4.132034	-1.673182	-0.465348
25	1	0	-2.151444	2.377399	0.382251
26	8	0	-2.385173	3.232840	0.794789
27	1	0	-1.837952	3.884172	0.343744
28	8	0	0.488071	0.378182	-0.778144
29	1	0	0.243347	1.013372	-1.515818
30	1	0	1.364149	0.752948	-0.223843
31	8	0	2.474935	1.112669	0.420018
32	1	0	2.630042	2.081566	0.521700
33	1	0	3.291511	0.685356	0.039997
34	8	0	4.571999	-0.203567	-0.547456
35	1	0	4.316743	-1.145845	-0.690942
36	1	0	4.960470	0.100218	-1.375391
37	8	0	2.938022	3.768973	0.732406
38	1	0	2.546331	4.385864	0.102495
39	1	0	2.774185	4.149751	1.603630
40	8	0	3.732764	-2.778604	-0.881414
41	1	0	4.308042	-3.481572	-0.559931
42	1	0	2.857990	-2.932422	-0.462473
43	1	0	-3.560223	0.397228	-0.710192
44	8	0	-4.479424	0.058875	-0.762385
45	1	0	-4.817257	0.331605	-1.622532

SCF Done: E(RB3LYP) = -1069.15354516   A.U. after   1 cycles

	1	2	3
	A	A	A
Frequencies --	-174.1618	13.7313	22.8007

Zero-point correction= 0.379663 (a.u.)

Thermal correction to Energy= 0.413409

Thermal correction to Enthalpy= 0.414354

Thermal correction to Gibbs Free Energy= 0.311341

Sum of electronic and zero-point Energies= -1068.773882

Sum of electronic and thermal Energies= -1068.740136

Sum of electronic and thermal Enthalpies= -1068.739192

Sum of electronic and thermal Free Energies= -1068.842204

	E (Thermal)	CV	S
	KCal/Mol	Cal/Mol-Kelvin	Cal/Mol-Kelvin
Total	259.418	113.698	216.808

==bold orange 11==

str18x.revhigh.log

Standard orientation:

Center Number	Atomic Number	Atomic Type	Coordinates (Angstroms) X	Y	Z
1	6	0	-0.793872	-0.402579	1.597754
2	7	0	0.421895	-1.229010	1.795980
3	6	0	-2.018113	-1.177891	2.078824
4	1	0	-1.940482	-1.358194	3.153839
5	1	0	-2.929957	-0.610270	1.897656
6	1	0	-2.102435	-2.149428	1.584762
7	1	0	-0.723316	0.526160	2.175850
8	6	0	-0.954138	0.116142	0.139657
9	7	0	-1.081518	-1.126296	-0.854518
10	1	0	1.250669	-0.642678	1.845901
11	1	0	0.358926	-1.684788	2.703280
12	1	0	0.830322	-2.521307	0.685074
13	8	0	-1.969150	0.906608	-0.052646
14	1	0	-0.604522	3.394571	-1.332511
15	8	0	0.992257	-3.106470	-0.114088
16	1	0	-0.333318	-1.828174	-0.749306
17	1	0	0.644193	-3.979721	0.102547
18	1	0	-2.004651	-1.595084	-0.774948
19	8	0	0.170214	2.963099	-1.758894
20	1	0	-1.024610	-0.739834	-1.796604

21	1	0	-0.010407	2.954557	-2.705468
22	8	0	-3.726108	-2.236535	-1.008008
23	1	0	-4.090095	-2.939395	-0.458894
24	1	0	-4.227909	-1.420585	-0.782655
25	1	0	-2.026645	2.606195	-0.055011
26	8	0	-2.021947	3.573550	-0.270516
27	1	0	-1.942825	4.041633	0.567461
28	8	0	0.310596	0.693913	-0.268097
29	1	0	0.147391	1.455592	-0.881417
30	1	0	1.770964	0.748986	0.239214
31	8	0	2.727398	0.771377	0.582556
32	1	0	3.020909	1.715676	0.787989
33	1	0	3.390210	0.280466	-0.072108
34	8	0	4.312934	-0.453054	-0.894197
35	1	0	4.057573	-1.410255	-1.007107
36	1	0	4.455707	-0.088224	-1.775551
37	8	0	3.546796	3.167939	1.139626
38	1	0	3.222743	3.895529	0.594030
39	1	0	3.445995	3.455555	2.055664
40	8	0	3.530453	-2.979869	-1.129424
41	1	0	4.091166	-3.651314	-0.725184
42	1	0	2.633467	-3.094389	-0.737831
43	1	0	-3.627553	0.555939	-0.219760
44	8	0	-4.568666	0.263576	-0.327974
45	1	0	-4.955626	0.842827	-0.993257

SCF Done: E(RB3LYP) = -1069.16375749 A.U. after 1 cycles

Zero-point correction= 0.382443 (a.u.)

Thermal correction to Energy= 0.415276

Thermal correction to Enthalpy= 0.416221

Thermal correction to Gibbs Free Energy= 0.315166

Sum of electronic and zero-point Energies= -1068.781314

Sum of electronic and thermal Energies= -1068.748481

Sum of electronic and thermal Enthalpies= -1068.747537

Sum of electronic and thermal Free Energies= -1068.848591

	E (Thermal)	CV	S
	KCal/Mol	Cal/Mol-Kelvin	Cal/Mol-Kelvin
Total	260.590	112.220	212.687

==bold orange 12==

str18x.high.log

Standard orientation:

Center Number	Atomic Number	Atomic Type	Coordinates (Angstroms) X	Y	Z
1	6	0	-0.730784	-0.498682	1.695034
2	7	0	0.496312	-1.323572	1.725551
3	6	0	-1.939973	-1.359191	2.046779
4	1	0	-1.831815	-1.741753	3.064635
5	1	0	-2.859409	-0.777224	2.004619
6	1	0	-2.031376	-2.212906	1.372347
7	1	0	-0.660844	0.307191	2.440152
8	6	0	-0.914190	0.293012	0.385948
9	7	0	-1.118079	-1.131433	-0.962601
10	1	0	1.322619	-0.737297	1.809150
11	1	0	0.478642	-1.879378	2.577801
12	1	0	0.893068	-2.530164	0.498032
13	8	0	-1.942984	0.997949	0.241319
14	1	0	-0.718085	3.203747	-1.702035
15	8	0	1.103626	-3.133482	-0.272939
16	1	0	-0.412240	-1.869795	-0.947353
17	1	0	0.835990	-4.021487	-0.009545
18	1	0	-2.053383	-1.551645	-0.948310
19	8	0	0.142000	2.761826	-1.883246
20	1	0	-1.023474	-0.618931	-1.835656
21	1	0	0.142062	2.532328	-2.819471
22	8	0	-3.999159	-2.090705	-1.119527
23	1	0	-4.357759	-2.858950	-0.662507
24	1	0	-4.372950	-1.308530	-0.660562

25	1	0	-2.226815	2.635272	-0.404975
26	8	0	-2.289042	3.472266	-0.915311
27	1	0	-2.383720	4.179267	-0.267616
28	8	0	0.293322	0.810003	-0.064820
29	1	0	0.151560	1.505143	-0.768469
30	1	0	1.857339	0.807439	0.364895
31	8	0	2.814842	0.771576	0.654982
32	1	0	3.139080	1.677854	0.965296
33	1	0	3.445328	0.337292	-0.092150
34	8	0	4.296568	-0.295874	-0.998563
35	1	0	4.053415	-1.252387	-1.161823
36	1	0	4.370287	0.136603	-1.857631
37	8	0	3.725445	3.049280	1.477283
38	1	0	3.415281	3.852347	1.040279
39	1	0	3.664054	3.220355	2.425474
40	8	0	3.570827	-2.805344	-1.367522
41	1	0	4.174865	-3.484768	-1.047933
42	1	0	2.696224	-2.987461	-0.946581
43	1	0	-3.672018	0.578901	0.253919
44	8	0	-4.602504	0.270806	0.184782
45	1	0	-5.077400	0.966733	-0.282700

SCF Done: E(RB3LYP) = -1069.16026022   A.U. after   1 cycles

	1	2	3
	A	A	A
Frequencies --	-227.6949	13.4604	21.9733

Zero-point correction= 0.380058 (a.u.)

Thermal correction to Energy= 0.413402

Thermal correction to Enthalpy= 0.414346

Thermal correction to Gibbs Free Energy= 0.312283

Sum of electronic and zero-point Energies= -1068.780202

Sum of electronic and thermal Energies= -1068.746858

Sum of electronic and thermal Enthalpies= -1068.745914

Sum of electronic and thermal Free Energies= -1068.847978

	E (Thermal)	CV	S
	KCal/Mol	Cal/Mol-Kelvin	Cal/Mol-Kelvin
Total	259.414	112.890	214.811

==bold orange 13==

str17r.rev.high.log R-alanine product

Standard orientation:

Center Number	Atomic Number	Atomic Type	Coordinates (Angstroms) X	Y	Z
1	6	0	-2.797216	-1.092078	-0.376019
2	7	0	-2.910038	-0.660444	1.033860
3	6	0	-3.602998	-2.361490	-0.707745
4	1	0	-4.661588	-2.178909	-0.509854
5	1	0	-3.496450	-2.629667	-1.761829
6	1	0	-3.272463	-3.203291	-0.095271
7	1	0	-3.150764	-0.273383	-1.004095
8	6	0	-1.336607	-1.309631	-0.750314
9	7	0	-0.165288	1.961758	-1.394902
10	1	0	-3.894414	-0.663337	1.293425
11	1	0	-2.458781	-1.343855	1.638569
12	1	0	-2.437366	1.042245	1.486525
13	8	0	-0.846685	-0.860809	-1.777873
14	8	0	-0.680515	-2.056406	0.119038
15	1	0	0.948758	-0.910492	-2.590374
16	8	0	1.917494	-0.902211	-2.678550
17	1	0	2.472434	0.737746	-2.359985
18	1	0	2.188356	-1.496284	-1.954185
19	8	0	1.966429	-2.470769	-0.333506
20	1	0	2.202922	-3.406236	-0.353246
21	1	0	2.531859	-2.052803	0.360819
22	1	0	-0.798914	2.774013	-1.274489
23	8	0	-2.179649	3.690612	-0.475892
24	1	0	-2.288238	3.200387	0.366921

25	8	0	-2.175644	1.976613	1.724826
26	1	0	-2.119353	4.622887	-0.240873
27	1	0	-2.660951	2.196390	2.528450
28	8	0	1.144933	-0.835917	3.124101
29	1	0	0.756320	-0.143543	2.546687
30	1	0	1.150001	-0.466968	4.014451
31	8	0	3.347555	-1.104726	1.589119
32	1	0	2.669447	-1.069578	2.307196
33	1	0	4.149042	-1.477956	1.974378
34	1	0	0.035973	1.575161	-0.451926
35	1	0	3.209249	2.079652	-2.696706
36	1	0	0.279986	-2.184381	-0.121146
37	8	0	2.606279	1.671893	-2.064881
38	1	0	-0.627611	1.230390	-1.940298
39	1	0	3.051552	1.694003	-0.210440
40	8	0	0.438437	1.167062	1.310649
41	1	0	1.318781	1.603467	1.302820
42	1	0	-0.240199	1.743615	1.706865
43	8	0	3.085956	1.664088	0.764843
44	1	0	0.737029	2.200740	-1.831702
45	1	0	3.356881	0.754550	0.986084

SCF Done: E(RB3LYP) = -1069.22361071   A.U. after   1 cycles

Zero-point correction= 0.386397 (a.u.)
Thermal correction to Energy= 0.419409
Thermal correction to Enthalpy= 0.420353
Thermal correction to Gibbs Free Energy= 0.323624
Sum of electronic and zero-point Energies= -1068.837214
Sum of electronic and thermal Energies= -1068.804202
Sum of electronic and thermal Enthalpies= -1068.803258
Sum of electronic and thermal Free Energies= -1068.899987

	E (Thermal)	CV	S
	KCal/Mol	Cal/Mol-Kelvin	Cal/Mol-Kelvin
Total	263.183	114.086	203.584

==bold orange 14==

str19aa.high.log

Standard orientation:

Center Number	Atomic Number	Atomic Type	Coordinates (Angstroms) X	Y	Z
1	6	0	2.205898	-1.572155	-0.737141
2	7	0	1.699741	-0.183406	-0.713407
3	6	0	2.701817	-1.968206	-2.135500
4	1	0	3.508803	-1.292768	-2.422808
5	1	0	3.085011	-2.989204	-2.130493
6	1	0	1.899745	-1.894485	-2.871529
7	1	0	3.028388	-1.701433	-0.023545
8	6	0	1.134653	-2.481284	-0.292675
9	7	0	0.296630	-3.194535	0.045628
10	1	0	1.558889	0.114914	0.261795
11	1	0	2.424969	0.425395	-1.110670
12	1	0	0.370205	0.110570	-1.445243
13	8	0	1.175762	0.662930	2.075495
14	1	0	0.208455	0.653799	2.207678
15	1	0	1.559190	0.186726	2.833598
16	8	0	-0.545246	0.387762	-1.902007
17	1	0	-1.601176	0.306737	-1.207302
18	1	0	-0.619773	-0.051180	-2.757907
19	8	0	-2.543898	0.295975	-0.550651
20	1	0	-3.045209	-0.584044	-0.608455
21	1	0	-3.168298	1.054842	-0.791905
22	8	0	3.870218	1.516815	-1.890574
23	1	0	3.699675	2.015136	-2.697139
24	8	0	2.297526	-0.730259	4.293412
25	1	0	2.749219	-1.564026	4.117470
26	1	0	2.885287	-0.245430	4.884674
27	8	0	-3.844914	-1.939517	-0.604647
28	1	0	-4.248079	-2.199149	-1.440776

29	1	0	-3.357881	-2.730919	-0.267911
30	8	0	-4.165858	2.238307	-1.118794
31	1	0	-4.115565	3.038748	-0.549301
32	1	0	-4.178334	2.550300	-2.030769
33	8	0	-2.439176	-4.070100	0.314922
34	1	0	-1.483623	-3.870157	0.303301
35	1	0	-2.636564	-4.377308	1.206992
36	8	0	-4.042214	4.463879	0.479645
37	1	0	-4.759162	4.594406	1.111914
38	1	0	-3.229858	4.619248	0.976206
39	8	0	-1.639600	0.606079	2.225466
40	1	0	-2.119872	1.327745	2.646606
41	1	0	-2.003789	0.539436	1.326306
42	1	0	4.383518	2.117883	-1.315175
43	8	0	5.348681	3.218317	-0.217205
44	1	0	4.952800	3.459363	0.628773
45	1	0	6.253959	2.958809	-0.008535

SCF Done: E(RB3LYP) = -1069.16065833 A.U. after 1 cycles

	1	2	3
	A	A	A
Frequencies --	-388.0720	2.8266	9.2771

Zero-point correction= 0.367646 (a.u.)

Thermal correction to Energy= 0.406948

Thermal correction to Enthalpy= 0.407892

Thermal correction to Gibbs Free Energy= 0.282915

Sum of electronic and zero-point Energies= -1068.793013

Sum of electronic and thermal Energies= -1068.753710

Sum of electronic and thermal Enthalpies= -1068.752766

Sum of electronic and thermal Free Energies= -1068.877743

	E (Thermal)	CV	S
	KCal/Mol	Cal/Mol-Kelvin	Cal/Mol-Kelvin
Total	255.364	123.344	263.036

==bold orange 15==

str19aa.forhigh.log

Standard orientation:

Center Number	Atomic Number	Atomic Type	Coordinates (Angstroms) X	Y	Z
1	6	0	2.470511	-1.348467	-0.761936
2	7	0	1.916265	0.039074	-0.717990
3	6	0	2.941829	-1.726522	-2.170905
4	1	0	3.705263	-1.016731	-2.490674
5	1	0	3.376551	-2.725878	-2.156894
6	1	0	2.113892	-1.711150	-2.880732
7	1	0	3.313018	-1.380640	-0.067831
8	6	0	1.458943	-2.291251	-0.260995
9	7	0	0.673152	-3.044088	0.110868
10	1	0	1.665366	0.317837	0.268646
11	1	0	2.634937	0.708757	-1.075913
12	1	0	1.052588	0.128287	-1.301774
13	8	0	1.186286	0.776533	1.818427
14	1	0	0.210103	0.763794	1.926991
15	1	0	1.567279	0.327932	2.597653
16	8	0	-0.482712	0.335429	-2.115348
17	1	0	-1.301154	0.230699	-1.574104
18	1	0	-0.658829	-0.092304	-2.960403
19	8	0	-2.680546	0.117721	-0.495199
20	1	0	-3.083549	-0.780614	-0.472339
21	1	0	-3.397499	0.743343	-0.746548
22	8	0	3.921556	1.726545	-1.685705
23	1	0	3.752102	2.227164	-2.491555
24	8	0	2.300706	-0.500626	4.066340
25	1	0	2.730649	-1.355038	3.942404
26	1	0	2.903683	0.008456	4.620858
27	8	0	-3.760623	-2.407349	-0.347344
28	1	0	-4.179776	-2.765816	-1.137234

29	1	0	-3.136102	-3.099184	-0.035535
30	8	0	-4.679595	1.871452	-1.184319
31	1	0	-4.838773	2.647880	-0.608705
32	1	0	-4.687540	2.205433	-2.087951
33	8	0	-1.942731	-4.286241	0.536205
34	1	0	-1.031307	-3.948414	0.474930
35	1	0	-2.045335	-4.605643	1.439791
36	8	0	-5.147513	4.062457	0.466636
37	1	0	-5.863218	3.989531	1.109257
38	1	0	-4.399722	4.422764	0.957942
39	8	0	-1.566553	0.702325	1.969094
40	1	0	-2.039406	1.450179	2.349822
41	1	0	-1.999259	0.520466	1.104062
42	1	0	4.378711	2.341709	-1.073515
43	8	0	5.223686	3.448910	0.048572
44	1	0	4.791324	3.662496	0.884037
45	1	0	6.136696	3.239970	0.279309

SCF Done: E(RB3LYP) = -1069.18398567 A.U. after 1 cycles

Zero-point correction= 0.373568 (a.u.)
Thermal correction to Energy= 0.414049
Thermal correction to Enthalpy= 0.414993
Thermal correction to Gibbs Free Energy= 0.288431
Sum of electronic and zero-point Energies= -1068.810418
Sum of electronic and thermal Energies= -1068.769937
Sum of electronic and thermal Enthalpies= -1068.768993
Sum of electronic and thermal Free Energies= -1068.895555

	E (Thermal)	CV	S
	KCal/Mol	Cal/Mol-Kelvin	Cal/Mol-Kelvin
Total	259.820	126.575	266.372

==bold orange 16A==

str06da.high.log

Standard orientation:

Center Number	Atomic Number	Atomic Type	Coordinates (Angstroms) X	Y	Z
1	6	0	-4.178255	0.545029	-0.750882
2	7	0	-5.585948	0.235451	-0.416035
3	6	0	-3.755963	-0.029173	-2.106232
4	1	0	-4.331491	0.470581	-2.886849
5	1	0	-2.697208	0.147370	-2.286125
6	1	0	-3.952807	-1.102213	-2.150433
7	1	0	-3.979497	1.617232	-0.726148
8	6	0	-3.414671	-0.073386	0.370737
9	7	0	-3.304197	-0.744073	1.319897
10	1	0	-6.070204	1.067759	-0.095343
11	1	0	-6.074148	-0.115312	-1.233794
12	1	0	-5.899332	-1.090173	1.082345
13	8	0	-1.520407	0.922174	-0.134214
14	1	0	-1.203493	1.320028	0.694771
15	1	0	-0.837699	0.233484	-0.306003
16	8	0	0.689941	-0.703728	-0.108435
17	1	0	0.872683	-1.630723	-0.359481
18	1	0	1.473318	-0.231391	-0.470880
19	8	0	3.070553	0.444984	-0.970542
20	1	0	3.076069	1.252602	-0.406284
21	1	0	3.679138	-0.204528	-0.566628
22	8	0	2.013711	-2.917892	-1.267236
23	1	0	2.109091	-2.414191	-2.098054
24	1	0	2.852610	-2.745495	-0.802049
25	8	0	-5.577708	-1.570047	1.884928
26	1	0	-4.240651	-1.266610	1.743158
27	1	0	-5.796517	-2.505667	1.774918
28	8	0	2.741971	2.500719	0.805067
29	1	0	2.559458	3.419287	0.529765
30	1	0	1.953104	2.189182	1.285899
31	8	0	4.419596	-1.856757	-0.050015
32	1	0	5.255036	-2.087515	-0.473012

33	1	0	4.563041	-1.984911	0.913366
34	8	0	2.514814	-0.980644	-3.319199
35	1	0	1.843315	-0.634162	-3.916863
36	1	0	2.684588	-0.273207	-2.668192
37	8	0	4.819491	-2.217937	2.658278
38	1	0	4.691168	-1.455795	3.235560
39	1	0	4.348193	-2.943940	3.084183
40	8	0	0.491172	1.147961	1.971028
41	1	0	0.629126	0.275167	1.555206
42	1	0	0.436104	0.991702	2.920827
43	8	0	2.257763	5.163045	0.012562
44	1	0	2.905929	5.823175	0.285429
45	1	0	2.108260	5.328762	-0.925818

SCF Done: E(RB3LYP) = -1069.11108694 A.U. after 1 cycles

	1	2	3
	A	A	A
Frequencies --	-329.4190	10.1163	13.7706

Zero-point correction= 0.372455 (a.u.)

Thermal correction to Energy= 0.409722

Thermal correction to Enthalpy= 0.410666

Thermal correction to Gibbs Free Energy= 0.295226

Sum of electronic and zero-point Energies= -1068.738632

Sum of electronic and thermal Energies= -1068.701365

Sum of electronic and thermal Enthalpies= -1068.700421

Sum of electronic and thermal Free Energies= -1068.815861

	E (Thermal)	CV	S
	KCal/Mol	Cal/Mol-Kelvin	Cal/Mol-Kelvin
Total	257.105	121.694	242.965

==bold orange 16B==

str06dj.high.log

Standard orientation:

Center Number	Atomic Number	Atomic Type	Coordinates (Angstroms) X	Y	Z
1	6	0	-1.335184	-0.281491	-1.001377
2	7	0	-2.028026	-1.520178	-0.511890
3	6	0	-1.580939	-0.086645	-2.498941
4	1	0	-2.648262	0.013129	-2.706823
5	1	0	-1.080341	0.822649	-2.832786
6	1	0	-1.184757	-0.928520	-3.071175
7	1	0	-1.777847	0.546462	-0.446420
8	6	0	0.155758	-0.391077	-0.684659
9	7	0	0.905529	-1.347936	-0.614451
10	1	0	-1.993491	-1.561899	0.527579
11	1	0	-3.024322	-1.519778	-0.795000
12	1	0	-1.573637	-2.378937	-0.878593
13	8	0	0.650880	1.055671	-0.571897
14	1	0	0.090658	1.609828	0.048976
15	1	0	1.844492	1.205329	-0.375387
16	8	0	3.012765	1.342001	-0.166344
17	1	0	3.395620	0.401116	-0.114190
18	1	0	3.471731	1.848475	-0.880249
19	8	0	3.572045	-1.158703	-0.121687
20	1	0	2.593487	-1.340643	-0.274641
21	1	0	3.825721	-1.580402	0.720739
22	8	0	-2.022838	-1.484568	2.321306
23	1	0	-1.502358	-2.140888	2.798106
24	1	0	-1.837720	-0.629304	2.755470
25	8	0	-4.822311	-1.579071	-1.188409
26	1	0	-5.430635	-0.961982	-0.764579
27	1	0	-5.133996	-1.663183	-2.097407
28	8	0	4.341747	-2.374407	2.323298
29	1	0	4.994520	-3.083396	2.284904
30	1	0	4.645528	-1.790127	3.027978
31	8	0	4.318385	2.746430	-2.079547
32	1	0	3.985915	2.710285	-2.984563

33	1	0	4.484497	3.680113	-1.901865
34	8	0	-0.749481	2.515694	1.116634
35	1	0	-1.460908	3.116990	0.817053
36	1	0	-1.065465	2.073683	1.925581
37	8	0	-1.539759	1.073017	3.461868
38	1	0	-0.830312	1.080297	4.117829
39	1	0	-2.313703	1.423179	3.922366
40	8	0	-2.766417	4.243603	0.269420
41	1	0	-2.588113	5.190448	0.315786
42	1	0	-3.154462	4.100340	-0.601965
43	8	0	-0.305878	-3.714402	-1.173595
44	1	0	0.283773	-2.918516	-1.003198
45	1	0	-0.109061	-4.014790	-2.067645

SCF Done: E(RB3LYP) = -1069.12646750   A.U. after   1 cycles

	1	2	3
	A	A	A
Frequencies --	-664.9255	12.9836	16.0340

Zero-point correction= 0.371377 (a.u.)

Thermal correction to Energy= 0.409076

Thermal correction to Enthalpy= 0.410020

Thermal correction to Gibbs Free Energy= 0.294438

Sum of electronic and zero-point Energies= -1068.755090

Sum of electronic and thermal Energies= -1068.717392

Sum of electronic and thermal Enthalpies= -1068.716448

Sum of electronic and thermal Free Energies= -1068.832029

	E (Thermal)	CV	S
	KCal/Mol	Cal/Mol-Kelvin	Cal/Mol-Kelvin
Total	256.699	120.021	243.262

==bold orange 17==

str06dj.rev.high.log

Standard orientation:

Center Number	Atomic Number	Atomic Type	Coordinates (Angstroms) X	Y	Z
1	6	0	-1.398494	-0.298224	-0.854060
2	7	0	-2.089115	-1.572210	-0.470170
3	6	0	-1.606195	0.005642	-2.340141
4	1	0	-2.670126	0.106497	-2.561822
5	1	0	-1.115068	0.946029	-2.594654
6	1	0	-1.183564	-0.784707	-2.963018
7	1	0	-1.862629	0.484587	-0.254912
8	6	0	0.089673	-0.368602	-0.510373
9	7	0	0.764866	-1.420423	-0.730103
10	1	0	-2.038869	-1.699173	0.562168
11	1	0	-3.092565	-1.528207	-0.731433
12	1	0	-1.666946	-2.399812	-0.934915
13	8	0	0.624297	0.772727	-0.043334
14	1	0	-0.051499	1.422814	0.322298
15	1	0	2.489386	1.399295	0.006135
16	8	0	3.451345	1.472946	0.109683
17	1	0	3.837462	-0.315279	-0.063535
18	1	0	3.756473	2.099119	-0.573882
19	8	0	3.772046	-1.280368	-0.206021
20	1	0	1.757886	-1.333056	-0.479874
21	1	0	4.203877	-1.700474	0.558313
22	8	0	-2.029871	-1.565272	2.348000
23	1	0	-1.366599	-2.050611	2.851447
24	1	0	-2.061195	-0.669246	2.735888
25	8	0	-4.878150	-1.516906	-1.093782
26	1	0	-5.462324	-0.893520	-0.645801
27	1	0	-5.204798	-1.570689	-1.999890
28	8	0	5.030777	-2.650712	1.967778
29	1	0	5.990919	-2.589409	2.035020
30	1	0	4.707153	-2.511350	2.865569
31	8	0	4.483693	3.261144	-1.811161
32	1	0	4.255412	3.141283	-2.740586

33	1	0	4.395274	4.207777	-1.648983
34	8	0	-0.985914	2.454606	1.110422
35	1	0	-1.584545	3.088439	0.664191
36	1	0	-1.460344	2.086478	1.878613
37	8	0	-2.189573	1.110423	3.323644
38	1	0	-1.694362	1.262859	4.139292
39	1	0	-3.100656	1.356349	3.531504
40	8	0	-2.670487	4.263216	-0.153405
41	1	0	-2.448641	5.199441	-0.083944
42	1	0	-2.875790	4.118681	-1.084773
43	8	0	-0.527532	-3.705996	-1.581815
44	1	0	0.131286	-2.991023	-1.394046
45	1	0	-0.469821	-3.894019	-2.525088

SCF Done: E(RB3LYP) = -1069.18005860   A.U. after   2 cycles

Zero-point correction= 0.376970 (a.u.)

Thermal correction to Energy= 0.416339

Thermal correction to Enthalpy= 0.417283

Thermal correction to Gibbs Free Energy= 0.296031

Sum of electronic and zero-point Energies= -1068.803089

Sum of electronic and thermal Energies= -1068.763720

Sum of electronic and thermal Enthalpies= -1068.762776

Sum of electronic and thermal Free Energies= -1068.884028

	E (Thermal)	CV	S
	KCal/Mol	Cal/Mol-Kelvin	Cal/Mol-Kelvin
Total	261.256	123.787	255.196

==bold orange 18==

str06dg.high.log

Standard orientation:

Center Number	Atomic Number	Atomic Type	Coordinates (Angstroms) X	Y	Z

1	6	0	-1.462188	-0.295533	-0.783221
2	7	0	-2.146059	-1.561737	-0.394599
3	6	0	-1.702334	0.024719	-2.265214
4	1	0	-2.771233	0.154672	-2.441881
5	1	0	-1.196193	0.951918	-2.537453
6	1	0	-1.331001	-0.777711	-2.904614
7	1	0	-1.876742	0.507232	-0.173831
8	6	0	0.037332	-0.362956	-0.497403
9	7	0	0.720539	-1.424574	-0.741939
10	1	0	-2.129217	-1.653366	0.634878
11	1	0	-3.135149	-1.525809	-0.686888
12	1	0	-1.636114	-2.460280	-0.871526
13	8	0	0.621301	0.727829	-0.060982
14	1	0	-0.017723	1.451437	0.297930
15	1	0	2.601088	1.347169	0.061172
16	8	0	3.558023	1.293870	0.194840
17	1	0	3.750797	-0.496106	-0.102239
18	1	0	3.959326	1.936577	-0.421864
19	8	0	3.562288	-1.432787	-0.315154
20	1	0	1.737292	-1.417485	-0.550938
21	1	0	3.951748	-1.967076	0.401166
22	8	0	-2.067940	-1.358516	2.477185
23	1	0	-1.431054	-1.830769	3.024891
24	1	0	-2.066899	-0.437965	2.799801
25	8	0	-4.961240	-1.537341	-1.139338
26	1	0	-5.571565	-0.887941	-0.770478
27	1	0	-5.248428	-1.668239	-2.050766
28	8	0	4.667146	-3.101321	1.698785
29	1	0	5.628326	-3.160847	1.751802
30	1	0	4.371345	-2.989695	2.609964
31	8	0	4.854129	3.105227	-1.513817
32	1	0	4.736787	3.024773	-2.467819
33	1	0	4.774967	4.048148	-1.327183
34	8	0	-0.803421	2.524948	0.993510
35	1	0	-1.355267	3.161738	0.489622
36	1	0	-1.311408	2.231179	1.774757

37	8	0	-2.124557	1.416729	3.235748
38	1	0	-1.654868	1.608974	4.058169
39	1	0	-3.030723	1.717268	3.385819
40	8	0	-2.338808	4.326634	-0.409430
41	1	0	-2.074015	5.253592	-0.373675
42	1	0	-2.520317	4.147991	-1.339893
43	8	0	-0.693053	-3.409926	-1.364909
44	1	0	0.192617	-2.374128	-1.113137
45	1	0	-0.795546	-3.631230	-2.296484

SCF Done: E(RB3LYP) = -1069.16547556 A.U. after 1 cycles

	1	2	3
	A	A	A
Frequencies --	-507.7551	9.3279	13.7414

Zero-point correction= 0.371086 (a.u.)
Thermal correction to Energy= 0.409183
Thermal correction to Enthalpy= 0.410127
Thermal correction to Gibbs Free Energy= 0.292109
Sum of electronic and zero-point Energies= -1068.794389
Sum of electronic and thermal Energies= -1068.756293
Sum of electronic and thermal Enthalpies= -1068.755349
Sum of electronic and thermal Free Energies= -1068.873367

	E (Thermal)	CV	S
	KCal/Mol	Cal/Mol-Kelvin	Cal/Mol-Kelvin
Total	256.766	120.463	248.390

==bold orange 19==
str18cc.revhigh.log

Standard orientation:

Center Number	Atomic Number	Atomic Type	Coordinates (Angstroms) X	Y	Z

1	6	0	1.455567	-0.030209	0.859754
2	7	0	1.569240	1.401519	0.553673
3	6	0	2.112573	-0.354993	2.213701
4	1	0	1.583712	0.183947	3.001764
5	1	0	2.056833	-1.422546	2.432330
6	1	0	3.159953	-0.046999	2.221590
7	1	0	0.409205	-0.347004	0.906627
8	6	0	2.104353	-0.889193	-0.217295
9	7	0	3.197566	-0.520180	-0.822593
10	1	0	0.919929	1.630061	-0.201868
11	1	0	1.264602	1.935300	1.370338
12	1	0	3.213434	2.092546	0.138625
13	8	0	1.605838	-2.043058	-0.507995
14	1	0	0.632513	-2.236137	-0.140769
15	8	0	-0.678257	1.583792	-1.620321
16	1	0	-0.352977	1.507061	-2.525022
17	1	0	4.823632	-3.014722	-2.594070
18	1	0	-1.154775	2.440848	-1.586050
19	8	0	4.553888	-2.112645	-2.803776
20	1	0	3.630188	-1.135461	-1.523083
21	1	0	4.194675	-2.157055	-3.697910
22	8	0	-2.025709	4.007646	-1.507327
23	1	0	-2.980700	3.980168	-1.374151
24	1	0	-1.700614	4.672713	-0.888858
25	8	0	0.666120	3.153746	2.924948
26	1	0	-0.152066	2.971162	3.401437
27	1	0	1.298319	3.406887	3.607692
28	8	0	-0.720107	-2.572899	0.156157
29	1	0	-0.946194	-2.762926	1.091286
30	1	0	-1.350511	-1.885021	-0.186734
31	8	0	-2.250225	-0.667552	-0.923783
32	1	0	-1.750538	0.140352	-1.155482
33	1	0	-3.097687	-0.376369	-0.527318
34	8	0	-1.375543	-3.135359	2.785578
35	1	0	-1.860958	-3.953205	2.946866
36	1	0	-0.672257	-3.121804	3.445646

37	8	0	4.114836	2.181289	-0.266294
38	1	0	3.638634	0.382340	-0.620469
39	1	0	4.715377	2.398129	0.455344
40	8	0	-4.665980	0.147356	0.172447
41	1	0	-5.488283	-0.183025	-0.243915
42	1	0	-4.790512	0.040915	1.121782
43	8	0	-7.004117	-0.773869	-1.034023
44	1	0	-7.444080	-0.181995	-1.655771
45	1	0	-6.967400	-1.628738	-1.479267

SCF Done: E(RB3LYP) = -1069.18251640 A.U. after 1 cycles

Zero-point correction= 0.373381 (a.u.)

Thermal correction to Energy= 0.413625

Thermal correction to Enthalpy= 0.414570

Thermal correction to Gibbs Free Energy= 0.288549

Sum of electronic and zero-point Energies= -1068.809135

Sum of electronic and thermal Energies= -1068.768891

Sum of electronic and thermal Enthalpies= -1068.767947

Sum of electronic and thermal Free Energies= -1068.893967

	E (Thermal)	CV	S
	KCal/Mol	Cal/Mol-Kelvin	Cal/Mol-Kelvin
Total	259.554	124.397	265.232

==bold orange 20A==

str16bx.high.log

Standard orientation:

Center Number	Atomic Number	Atomic Type	Coordinates (Angstroms) X	Y	Z
1	6	0	-2.068120	1.093043	-0.850917
2	7	0	-2.374363	-0.134454	-1.622874
3	6	0	-1.913105	2.319318	-1.753547
4	1	0	-2.811386	2.425812	-2.365309

5	1	0	-1.795238	3.237317	-1.175868
6	1	0	-1.054763	2.212990	-2.421957
7	1	0	-2.902019	1.260754	-0.165035
8	6	0	-0.844218	0.846538	0.072672
9	7	0	-1.068307	-0.064070	1.156684
10	1	0	-3.350817	-0.099958	-1.904787
11	1	0	-1.839475	-0.114618	-2.489617
12	1	0	-2.201203	-1.920286	-1.328928
13	8	0	-0.313676	1.996964	0.615660
14	8	0	0.217603	0.258808	-0.844760
15	1	0	0.111884	2.595243	-0.047891
16	8	0	1.312085	3.451324	-0.972829
17	1	0	1.574391	4.331616	-0.679955
18	1	0	2.096601	2.883877	-0.877837
19	8	0	2.714748	1.039236	-0.548167
20	1	0	3.139172	0.436410	-1.184327
21	1	0	3.050512	0.717487	0.311955
22	1	0	-1.862790	-0.688177	1.014028
23	8	0	-3.138947	-2.395726	1.359875
24	1	0	-2.794501	-2.847640	0.568776
25	8	0	-1.966980	-2.872170	-1.152397
26	1	0	-2.890440	-2.952292	2.105926
27	1	0	-2.335678	-3.395302	-1.874122
28	8	0	3.488073	-1.608730	-1.370792
29	1	0	2.634282	-2.056689	-1.484605
30	1	0	4.103856	-2.044293	-1.971694
31	8	0	3.615572	-0.840876	1.331414
32	1	0	3.841957	-1.319153	0.510688
33	1	0	4.386995	-0.893528	1.907839
34	1	0	0.332865	-0.872851	-0.832801
35	1	0	-0.683479	2.462034	2.516344
36	1	0	1.135468	0.628914	-0.644598
37	8	0	-0.944511	2.147640	3.395810
38	1	0	-1.222453	0.465453	2.014053
39	1	0	-1.649935	2.738921	3.680621
40	8	0	0.598798	-2.145967	-0.795330

41	1	0	0.794749	-2.301023	0.176626
42	1	0	-0.236607	-2.631986	-1.010123
43	8	0	1.058272	-1.981661	1.790825
44	1	0	0.409315	-1.246121	1.798362
45	1	0	1.941772	-1.567375	1.833834

SCF Done: E(RB3LYP) = -1069.14981662 A.U. after 1 cycles

	1	2	3
	A	A	A
Frequencies --	-354.6854	16.1682	35.4762

Zero-point correction= 0.381341 (a.u.)
Thermal correction to Energy= 0.413646
Thermal correction to Enthalpy= 0.414590
Thermal correction to Gibbs Free Energy= 0.318494
Sum of electronic and zero-point Energies= -1068.768475
Sum of electronic and thermal Energies= -1068.736171
Sum of electronic and thermal Enthalpies= -1068.735227
Sum of electronic and thermal Free Energies= -1068.831323

	E (Thermal)	CV	S
	KCal/Mol	Cal/Mol-Kelvin	Cal/Mol-Kelvin
Total	259.567	112.291	202.251

==bold orange 20B==
str18cc.high.log
Standard orientation:

Center Number	Atomic Number	Atomic Type	Coordinates (Angstroms) X	Y	Z
1	6	0	1.517271	0.058786	0.876384
2	7	0	1.663865	1.428880	0.363551
3	6	0	2.269070	-0.106596	2.207891
4	1	0	1.835173	0.565478	2.950824

5	1	0	2.183822	-1.128898	2.580520
6	1	0	3.326761	0.138885	2.091663
7	1	0	0.465400	-0.188480	1.050622
8	6	0	2.032571	-0.980558	-0.116948
9	7	0	3.118739	-0.746220	-0.817622
10	1	0	0.984165	1.577132	-0.384829
11	1	0	1.420585	2.084849	1.108553
12	1	0	3.304611	1.974644	-0.213736
13	8	0	1.445263	-2.104582	-0.226504
14	1	0	0.322290	-2.240730	0.185503
15	8	0	-0.710315	1.451550	-1.716013
16	1	0	-0.428080	1.316192	-2.628274
17	1	0	4.652497	-3.485230	-2.342142
18	1	0	-1.166552	2.320428	-1.709024
19	8	0	4.290356	-2.657849	-2.680719
20	1	0	3.475795	-1.462535	-1.457130
21	1	0	3.808136	-2.902555	-3.479422
22	8	0	-1.995906	3.909007	-1.675246
23	1	0	-2.950724	3.912411	-1.538124
24	1	0	-1.649635	4.583283	-1.078639
25	8	0	0.939159	3.538515	2.507203
26	1	0	0.090171	3.516631	2.963791
27	1	0	1.578121	3.785824	3.185732
28	8	0	-0.827382	-2.451153	0.456799
29	1	0	-1.015476	-2.483532	1.427073
30	1	0	-1.429906	-1.769538	0.012570
31	8	0	-2.261393	-0.708972	-0.811990
32	1	0	-1.762395	0.076858	-1.117528
33	1	0	-3.116666	-0.392896	-0.447223
34	8	0	-1.376396	-2.546632	3.101108
35	1	0	-1.956307	-3.259596	3.395083
36	1	0	-0.636707	-2.543400	3.720720
37	8	0	4.194782	1.997231	-0.653272
38	1	0	3.616704	0.142512	-0.749523
39	1	0	4.818708	2.290162	0.019847
40	8	0	-4.679442	0.152518	0.183152

41	1	0	-5.485905	-0.229033	-0.221253
42	1	0	-4.813647	0.104011	1.135954
43	8	0	-6.961910	-0.926038	-0.989907
44	1	0	-7.452895	-0.366701	-1.603539
45	1	0	-6.864270	-1.773361	-1.440408

SCF Done: E(RB3LYP) = -1069.18182514 A.U. after 1 cycles

	1	2	3
	A	A	A
Frequencies --	-471.6637	7.1708	11.8921

Zero-point correction= 0.370337 (a.u.)

Thermal correction to Energy= 0.409916

Thermal correction to Enthalpy= 0.410860

Thermal correction to Gibbs Free Energy= 0.286679

Sum of electronic and zero-point Energies= -1068.811488

Sum of electronic and thermal Energies= -1068.771909

Sum of electronic and thermal Enthalpies= -1068.770965

Sum of electronic and thermal Free Energies= -1068.895146

	E (Thermal)	CV	S
	KCal/Mol	Cal/Mol-Kelvin	Cal/Mol-Kelvin
Total	257.226	122.670	261.362

==bold orange 21==

str15a.high.log

Standard orientation:

Center Number	Atomic Number	Atomic Type	Coordinates (Angstroms) X	Y	Z
1	6	0	2.295126	-0.509433	-0.900131
2	7	0	2.205808	0.774316	-1.632203
3	6	0	2.542628	-1.701669	-1.829147
4	1	0	3.438960	-1.512365	-2.423138

5	1	0	2.697921	-2.623903	-1.267823
6	1	0	1.702472	-1.844222	-2.514040
7	1	0	3.129978	-0.428543	-0.199280
8	6	0	1.014022	-0.708159	-0.048762
9	7	0	1.074771	0.190109	1.177585
10	1	0	3.133887	1.010265	-1.975102
11	1	0	1.631795	0.629888	-2.460978
12	1	0	1.531961	2.494933	-1.328543
13	8	0	0.907648	-1.978983	0.503185
14	8	0	-0.090324	-0.366711	-0.824299
15	1	0	0.473901	-2.610082	-0.122935
16	8	0	-0.625613	-3.611942	-1.033113
17	1	0	-0.758916	-4.523533	-0.749360
18	1	0	-1.480925	-3.158682	-0.915287
19	8	0	-2.495311	-1.600890	-0.488719
20	1	0	-3.016365	-1.013674	-1.064962
21	1	0	-2.899428	-1.454933	0.387130
22	1	0	1.539323	1.104835	1.020893
23	8	0	2.200969	2.832640	1.326421
24	1	0	1.831050	3.330573	0.571295
25	8	0	1.119693	3.376803	-1.143242
26	1	0	1.902585	3.286228	2.122760
27	1	0	1.456293	3.983789	-1.812646
28	8	0	-3.892754	0.854810	-1.102325
29	1	0	-3.166922	1.508259	-1.150461
30	1	0	-4.580154	1.160526	-1.704684
31	8	0	-3.865774	-0.169464	1.495139
32	1	0	-4.189286	0.311352	0.707999
33	1	0	-4.618997	-0.288565	2.084630
34	1	0	-0.900461	1.351555	-0.988407
35	1	0	2.149917	-2.334720	2.457576
36	1	0	-0.903901	-0.856268	-0.544941
37	8	0	2.290544	-1.584360	3.050991
38	1	0	1.592396	-0.308016	1.927269
39	1	0	3.215550	-1.629647	3.319738
40	8	0	-1.423046	2.157764	-0.825559

41	1	0	-1.535473	1.786685	1.090318
42	1	0	-0.790788	2.874581	-1.015178
43	8	0	-1.439829	1.261818	1.907005
44	1	0	0.112405	0.405886	1.521746
45	1	0	-2.263110	0.740521	1.953105

SCF Done: E(RB3LYP) = -1069.18236229 A.U. after 1 cycles

Zero-point correction= 0.386944 (a.u.)

Thermal correction to Energy= 0.419842

Thermal correction to Enthalpy= 0.420786

Thermal correction to Gibbs Free Energy= 0.324640

Sum of electronic and zero-point Energies= -1068.795418

Sum of electronic and thermal Energies= -1068.762520

Sum of electronic and thermal Enthalpies= -1068.761576

Sum of electronic and thermal Free Energies= -1068.857722

	E (Thermal)	CV	S
	KCal/Mol	Cal/Mol-Kelvin	Cal/Mol-Kelvin
Total	263.455	114.769	202.357

==bold orange 22==

str17r.high.log

Standard orientation:

Center Number	Atomic Number	Atomic Type	Coordinates (Angstroms) X	Y	Z
1	6	0	2.610767	-0.833317	-0.908813
2	7	0	2.610643	0.526645	-1.470512
3	6	0	3.086126	-1.903605	-1.914879
4	1	0	4.086650	-1.637993	-2.261056
5	1	0	3.130682	-2.888665	-1.449266
6	1	0	2.421728	-1.950962	-2.781385
7	1	0	3.274279	-0.845618	-0.045293
8	6	0	1.228010	-1.226071	-0.417355

9	7	0	1.174025	0.052973	1.401646
10	1	0	3.564184	0.750017	-1.746751
11	1	0	2.064693	0.541465	-2.330433
12	1	0	2.173200	2.026840	-0.607041
13	8	0	1.141921	-2.370505	0.160481
14	8	0	0.222072	-0.687847	-1.066189
15	1	0	0.192178	-2.638919	0.536555
16	8	0	-1.075323	-2.996007	1.123990
17	1	0	-1.262955	-2.365217	1.863965
18	1	0	-1.740096	-2.799511	0.438271
19	8	0	-2.153921	-1.780818	-1.131631
20	1	0	-2.327622	-2.266652	-1.947695
21	1	0	-2.870497	-1.101334	-1.043563
22	1	0	2.072473	0.294586	1.819120
23	8	0	3.405980	2.159622	2.192461
24	1	0	2.888691	2.568335	1.472951
25	8	0	1.875686	2.843159	-0.110509
26	1	0	3.232138	2.697782	2.972016
27	1	0	2.210697	3.599736	-0.606894
28	8	0	-3.125334	2.452546	-1.915532
29	1	0	-2.283247	2.558828	-1.428456
30	1	0	-3.613575	3.274192	-1.791504
31	8	0	-3.992695	0.164200	-0.732271
32	1	0	-3.822444	0.994926	-1.240520
33	1	0	-4.931829	-0.036384	-0.820723
34	1	0	-0.499544	1.270526	-0.831679
35	1	0	-1.708932	-1.131265	3.838014
36	1	0	-0.664704	-1.173864	-1.024617
37	8	0	-1.384190	-1.026786	2.937019
38	1	0	0.619832	-0.448830	2.095732
39	1	0	-1.880805	-0.271895	2.535462
40	8	0	-0.798704	2.090809	-0.410005
41	1	0	-1.948670	1.503659	1.081563
42	1	0	0.026441	2.608895	-0.300670
43	8	0	-2.603675	1.023823	1.620179
44	1	0	0.694684	0.931745	1.226757

45	1	0	-3.234261	0.678651	0.961546

SCF Done: E(RB3LYP) = -1069.17473491 A.U. after 1 cycles

	1	2	3
	A	A	A
Frequencies --	-181.5511	28.8467	33.6068

Zero-point correction= 0.383279 (a.u.)
Thermal correction to Energy= 0.415315
Thermal correction to Enthalpy= 0.416259
Thermal correction to Gibbs Free Energy= 0.321849
Sum of electronic and zero-point Energies= -1068.791456
Sum of electronic and thermal Energies= -1068.759420
Sum of electronic and thermal Enthalpies= -1068.758476
Sum of electronic and thermal Free Energies= -1068.852886

	E (Thermal)	CV	S
	KCal/Mol	Cal/Mol-Kelvin	Cal/Mol-Kelvin
Total	260.614	111.992	198.703

[3] Two transition states, A and B, with stoichiometry C11H36N2O11, which are shown in Figure 3

==TS A==
strts1.high.log

Standard orientation:

Center Number	Atomic Number	Atomic Type	Coordinates (Angstroms) X	Y	Z
1	6	0	-0.014818	-2.567574	-0.419846
2	8	0	0.988350	-1.912582	-0.054580
3	6	0	-0.293222	-2.813158	-1.875884
4	1	0	-1.318920	-3.141094	-2.043876
5	1	0	-0.078815	-1.925694	-2.472600

6	1	0	0.375865	-3.617079	-2.205304
7	1	0	-0.453094	-3.286903	0.279890
8	7	0	-1.721075	-1.250491	-0.002475
9	6	0	-3.121483	-1.718974	-0.078496
10	1	0	-1.493825	-0.920400	0.938128
11	1	0	-1.568211	-0.472757	-0.651484
12	1	0	1.421217	-1.686127	1.562908
13	8	0	1.660297	-1.328308	2.459609
14	1	0	1.987207	-2.070589	2.980786
15	1	0	2.864416	-0.001921	1.970799
16	8	0	3.378023	0.673327	1.479913
17	1	0	3.223220	0.446361	0.532279
18	1	0	2.493823	2.352677	1.596356
19	6	0	1.877147	3.232708	1.403386
20	7	0	1.205180	4.128615	1.141931
21	1	0	2.123872	-0.805690	-0.891035
22	8	0	2.765701	-0.094926	-1.113558
23	1	0	3.549206	-0.527048	-1.523405
24	8	0	6.094001	0.452190	1.846938
25	1	0	5.119388	0.569751	1.763223
26	1	0	6.451596	1.325015	2.043144
27	8	0	5.043065	-1.240764	-2.152934
28	1	0	5.343417	-0.929989	-3.013884
29	1	0	5.803035	-1.116886	-1.538946
30	8	0	-0.145345	3.743646	-1.511012
31	1	0	-0.405405	4.534584	-1.997529
32	1	0	0.177034	4.059343	-0.651423
33	8	0	7.080387	-0.850974	-0.355882
34	1	0	6.765979	-0.386028	0.453587
35	1	0	7.552415	-1.631937	-0.047076
36	8	0	-0.908504	-0.289057	2.911417
37	1	0	0.017219	-0.603919	2.894170
38	1	0	-0.854158	0.649628	3.121004
39	8	0	1.318763	1.587845	-2.827980
40	1	0	1.106916	2.419356	-2.372515
41	1	0	1.885822	1.071358	-2.218378

42	8	0	-1.341313	1.049233	-2.032329
43	1	0	-1.355446	1.947774	-1.670421
44	1	0	-0.454595	1.017758	-2.442735
45	6	0	-4.142761	-0.589312	0.033265
46	6	0	-4.124304	0.304047	1.112469
47	6	0	-5.075442	1.317778	1.213677
48	6	0	-6.064182	1.455225	0.237479
49	6	0	-5.136468	-0.441034	-0.939272
50	6	0	-6.092192	0.571730	-0.840248
51	1	0	-3.360900	0.212840	1.878027
52	1	0	-5.045628	2.001237	2.055165
53	1	0	-6.802869	2.244836	0.316141
54	1	0	-5.162586	-1.121997	-1.784233
55	1	0	-6.853290	0.671255	-1.606268
56	6	0	-3.370867	-2.806989	0.974316
57	1	0	-2.694715	-3.652588	0.829625
58	1	0	-3.220171	-2.414012	1.983494
59	1	0	-4.396530	-3.174658	0.903350
60	1	0	-3.247073	-2.167036	-1.069060

SCF Done: E(RB3LYP) = -1378.46093096   A.U. after   2 cycles

	1	2	3
	A	A	A
Frequencies --	-84.0999	11.7378	16.7597

Zero-point correction=   0.498519 (a.u.)

Thermal correction to Energy=   0.542460

Thermal correction to Enthalpy=   0.543404

Thermal correction to Gibbs Free Energy=   0.414246

Sum of electronic and zero-point Energies=   -1377.962412

Sum of electronic and thermal Energies=   -1377.918471

Sum of electronic and thermal Enthalpies=   -1377.917527

Sum of electronic and thermal Free Energies=   -1378.046685

E (Thermal)   CV   S

	KCal/Mol	Cal/Mol-Kelvin	Cal/Mol-Kelvin
Total	340.399	148.096	271.836

==TS B==

strts4.higha.log

Standard orientation:

Center Number	Atomic Number	Atomic Type	Coordinates (Angstroms) X	Y	Z
1	6	0	-0.043214	-2.267074	-1.034319
2	8	0	0.968331	-1.737653	-0.509130
3	7	0	-1.696973	-1.262000	-0.118901
4	6	0	-0.385746	-3.705916	-0.761758
5	1	0	0.323682	-4.315950	-1.333792
6	1	0	-0.267339	-3.950540	0.294736
7	1	0	-1.390236	-3.958088	-1.100105
8	1	0	-0.393865	-1.876802	-1.997560
9	6	0	-3.110402	-1.698768	-0.214783
10	1	0	-1.403549	-1.155853	0.855604
11	1	0	-1.569787	-0.360361	-0.587693
12	1	0	1.551262	-1.966947	1.075825
13	8	0	1.853736	-1.828568	2.011440
14	1	0	2.231353	-2.663710	2.310553
15	1	0	2.985097	-0.368115	1.810411
16	8	0	3.448979	0.429237	1.479394
17	1	0	3.246805	0.433597	0.513410
18	1	0	2.497129	1.993936	2.016612
19	6	0	1.830092	2.857544	2.036428
20	7	0	1.104295	3.748438	1.992368
21	1	0	2.039835	-0.451754	-1.072876
22	8	0	2.692252	0.278825	-1.179118
23	1	0	3.440896	-0.073479	-1.711540
24	8	0	6.182814	0.175215	1.635353
25	1	0	5.205189	0.297965	1.634711
26	1	0	6.552928	0.980753	2.012530

27	8	0	4.885116	-0.665962	-2.553110
28	1	0	5.130055	-0.219632	-3.370878
29	1	0	5.686474	-0.645924	-1.981052
30	8	0	-0.322402	3.953984	-0.637092
31	1	0	-0.647733	4.821082	-0.905735
32	1	0	0.019524	4.069924	0.264286
33	8	0	7.039952	-0.589687	-0.854544
34	1	0	6.774257	-0.318464	0.054415
35	1	0	7.544130	-1.404082	-0.751478
36	8	0	-0.707323	-1.041433	2.857110
37	1	0	0.226782	-1.292395	2.714603
38	1	0	-0.678706	-0.193009	3.312390
39	8	0	1.195923	2.255467	-2.470540
40	1	0	0.954540	2.949682	-1.835416
41	1	0	1.791657	1.639076	-1.995444
42	8	0	-1.374122	1.364195	-1.688366
43	1	0	-1.420881	2.171341	-1.154233
44	1	0	-0.514409	1.477607	-2.138861
45	6	0	-4.099343	-0.539287	-0.135503
46	6	0	-4.066755	0.371959	0.927878
47	6	0	-4.991913	1.411144	1.004332
48	6	0	-5.969950	1.555528	0.018309
49	6	0	-5.081395	-0.383708	-1.118145
50	6	0	-6.012366	0.653984	-1.043838
51	1	0	-3.312104	0.274951	1.701391
52	1	0	-4.950663	2.108494	1.833780
53	1	0	-6.688956	2.364743	0.077345
54	1	0	-5.117624	-1.078318	-1.951502
55	1	0	-6.764990	0.759025	-1.817417
56	6	0	-3.419160	-2.754203	0.855465
57	1	0	-2.743310	-3.606906	0.776943
58	1	0	-3.318283	-2.328134	1.856961
59	1	0	-4.442781	-3.116207	0.740471
60	1	0	-3.232770	-2.158762	-1.201227

SCF Done: E(RB3LYP) = -1378.45844669   A.U. after   2 cycles

	1	2	3
	A	A	A
Frequencies --	-101.9780	13.4815	16.1868

Zero-point correction= 0.498930 (a.u.)
Thermal correction to Energy= 0.542638
Thermal correction to Enthalpy= 0.543583
Thermal correction to Gibbs Free Energy= 0.415526
Sum of electronic and zero-point Energies= -1377.959516
Sum of electronic and thermal Energies= -1377.915808
Sum of electronic and thermal Enthalpies= -1377.914864
Sum of electronic and thermal Free Energies= -1378.042920

	E (Thermal)	CV	S
	KCal/Mol	Cal/Mol-Kelvin	Cal/Mol-Kelvin
Total	340.511	147.818	269.517

[4] The geometry of 8ext in Figure S2 with
with stoichiometry C3H48N2O21 along with that of 7ext

==Intermediate 7ext prior to 8ext(TS)==
str09dk.extfor.log
Standard orientation:

Center Number	Atomic Number	Atomic Type	Coordinates (Angstroms) X	Y	Z
1	6	0	1.892239	-0.150738	3.253368
2	7	0	0.828980	0.442053	2.348833
3	6	0	2.495326	0.935624	4.121751
4	1	0	2.976589	1.698704	3.505513
5	1	0	1.728966	1.407275	4.739307
6	1	0	3.245085	0.491655	4.777327
7	1	0	1.376408	-0.896651	3.863941
8	8	0	2.892462	-0.740495	2.475180

9	1	0	2.546692	-1.538095	1.990134
10	1	0	4.162858	0.217739	1.319114
11	1	0	0.193144	-0.280656	1.965365
12	1	0	0.210792	1.111854	2.855629
13	8	0	4.537191	0.804135	0.638883
14	1	0	2.940072	1.355039	0.049611
15	1	0	4.956027	0.218131	-0.031774
16	8	0	1.963868	1.380613	-0.076301
17	1	0	1.263352	0.925479	1.533294
18	1	0	1.715726	2.216730	-0.540353
19	6	0	-3.403562	1.366738	1.843490
20	7	0	-4.203531	1.138721	1.020810
21	8	0	-0.413359	-3.808670	1.784086
22	1	0	-0.853649	-4.417287	1.165608
23	1	0	-0.910499	-2.969729	1.730511
24	8	0	2.011211	-2.741906	0.941209
25	1	0	1.186690	-3.196797	1.222278
26	1	0	1.778986	-2.243541	0.129871
27	8	0	-1.366670	-1.241371	-1.401790
28	1	0	-1.956691	-0.505820	-1.674106
29	1	0	-1.755626	-2.081754	-1.732112
30	8	0	1.401057	-1.062786	-1.257052
31	1	0	1.564117	-0.161058	-0.896690
32	1	0	0.437711	-1.111896	-1.433771
33	1	0	-3.282959	1.104017	-0.831555
34	8	0	-2.968665	0.998195	-1.748505
35	1	0	-3.791906	0.983098	-2.290488
36	1	0	-1.837243	3.742814	-2.956131
37	8	0	-1.276625	3.012200	-2.673660
38	1	0	-1.886834	2.328495	-2.321785
39	1	0	-2.111287	-0.694667	1.630155
40	8	0	-1.341063	-1.204712	1.340292
41	1	0	-1.383334	-1.207491	0.353103
42	8	0	1.127846	3.601708	-1.393804
43	1	0	0.262034	3.434174	-1.820201
44	1	0	1.028461	4.421338	-0.874839

45	8	0	-1.146143	2.040220	3.578876
46	1	0	-1.083312	2.997453	3.671044
47	1	0	-1.954089	1.868441	3.033847
48	1	0	-5.085928	-0.689366	0.916092
49	8	0	-5.521917	-1.555091	0.837343
50	1	0	-5.041669	-2.126285	1.446358
51	1	0	-5.774247	1.633275	0.041915
52	8	0	-6.523189	1.796222	-0.568191
53	1	0	-6.820709	2.695282	-0.390008
54	8	0	-1.662065	-5.581843	-0.144253
55	1	0	-1.039531	-6.256531	-0.445424
56	1	0	-2.419267	-6.072285	0.201392
57	8	0	5.640170	-0.808653	-1.308894
58	1	0	4.944694	-1.140947	-1.913289
59	1	0	6.329790	-0.410705	-1.871406
60	8	0	7.667734	0.354841	-2.918903
61	1	0	7.729382	1.317337	-2.913655
62	1	0	8.572225	0.043747	-2.794869
63	8	0	3.514185	-1.700214	-2.899921
64	1	0	3.487449	-2.638883	-3.114902
65	1	0	2.695656	-1.519684	-2.385760
66	8	0	-5.411970	1.153225	-3.003234
67	1	0	-5.828059	0.367365	-3.374314
68	1	0	-5.937296	1.388769	-2.206182
69	8	0	0.888285	5.993448	0.105663
70	1	0	0.960577	5.930344	1.065372
71	1	0	1.471867	6.719714	-0.143834
72	8	0	-2.383478	-3.688829	-2.137583
73	1	0	-2.187891	-4.052669	-3.008176
74	1	0	-2.136384	-4.382506	-1.495841

SCF Done: E(RB3LYP) = -1833.49554196   A.U. after   1 cycles

Zero-point correction=   0.614929 (a.u.)

Thermal correction to Energy=   0.680000

Thermal correction to Enthalpy=   0.680944

Thermal correction to Gibbs Free Energy= 0.500939
Sum of electronic and zero-point Energies= -1832.880613
Sum of electronic and thermal Energies= -1832.815542
Sum of electronic and thermal Enthalpies= -1832.814598
Sum of electronic and thermal Free Energies= -1832.994603

	E (Thermal)	CV	S
	KCal/Mol	Cal/Mol-Kelvin	Cal/Mol-Kelvin
Total	426.707	210.331	378.853

==TS 8ext==

str09dk.ext.log

Standard orientation:

Center Number	Atomic Number	Atomic Type	Coordinates (Angstroms) X	Y	Z
1	6	0	1.583996	0.097853	-3.118148
2	7	0	0.736823	-0.523196	-2.082766
3	6	0	2.268389	-0.960836	-3.963896
4	1	0	2.863612	-1.628566	-3.335344
5	1	0	1.528303	-1.556968	-4.501566
6	1	0	2.926657	-0.485778	-4.692595
7	1	0	0.978721	0.751863	-3.755771
8	8	0	2.599473	0.908071	-2.511895
9	1	0	2.175498	1.677582	-2.051673
10	1	0	3.795340	0.253324	-1.448659
11	1	0	-0.012317	0.107417	-1.789637
12	1	0	0.277686	-1.364697	-2.443164
13	8	0	4.189012	-0.243376	-0.681471
14	1	0	3.218942	-0.676846	-0.190016
15	1	0	4.658667	0.411751	-0.062482
16	8	0	2.057888	-0.984951	0.144863
17	1	0	1.501651	-0.883870	-0.737088
18	1	0	1.906981	-1.891786	0.546959
19	6	0	-3.120440	-2.233684	-0.950907

20	7	0	-3.888405	-1.422121	-0.601519
21	8	0	-1.071271	3.350630	-2.100874
22	1	0	-1.608380	4.124821	-1.850610
23	1	0	-1.521458	2.563460	-1.735085
24	8	0	1.461010	2.912204	-1.094593
25	1	0	0.566669	3.150207	-1.428779
26	1	0	1.321670	2.498906	-0.219823
27	8	0	-1.475552	1.199477	1.897871
28	1	0	-1.888302	0.357683	2.187270
29	1	0	-1.939235	1.922580	2.368115
30	8	0	1.224080	1.443948	1.359794
31	1	0	1.472127	0.550198	1.051603
32	1	0	0.291644	1.370199	1.659468
33	1	0	-3.309481	-1.276525	1.527975
34	8	0	-2.732000	-1.252955	2.308808
35	1	0	-3.310284	-1.383715	3.091039
36	1	0	-1.661291	-3.369245	0.434281
37	8	0	-1.242147	-3.445176	1.305929
38	1	0	-1.665040	-2.727589	1.818396
39	1	0	-2.517649	0.301726	-0.969806
40	8	0	-1.836579	0.985603	-0.878646
41	1	0	-1.728702	1.109186	0.088253
42	8	0	1.517323	-3.296702	1.274425
43	1	0	0.540196	-3.401788	1.280609
44	1	0	1.892239	-4.122419	0.912392
45	8	0	-0.923236	-2.989012	-2.829800
46	1	0	-0.610176	-3.892144	-2.707935
47	1	0	-1.661804	-2.880920	-2.193715
48	1	0	-4.986094	-0.970085	-2.348404
49	8	0	-5.543662	-0.709587	-3.099228
50	1	0	-5.125314	-1.116498	-3.865699
51	1	0	-5.380846	-0.422280	0.169678
52	8	0	-6.088965	0.095158	0.596514
53	1	0	-6.895593	-0.404514	0.432330
54	8	0	-2.608502	5.631278	-1.409463
55	1	0	-2.191364	6.291201	-0.843130

56	1	0	-2.993740	6.132001	-2.138299
57	8	0	5.352227	1.427292	0.909820
58	1	0	4.731365	1.813289	1.567476
59	1	0	6.133980	1.104268	1.400548
60	8	0	7.609467	0.521561	2.290973
61	1	0	7.786186	-0.426690	2.295265
62	1	0	8.451752	0.935670	2.068434
63	8	0	3.450218	2.422266	2.645698
64	1	0	3.393774	3.379015	2.744465
65	1	0	2.587110	2.137613	2.270685
66	8	0	-4.333640	-1.639734	4.536361
67	1	0	-4.021832	-2.263332	5.203050
68	1	0	-4.606406	-0.855748	5.027808
69	8	0	2.621432	-5.678191	0.255418
70	1	0	3.255114	-5.616130	-0.469139
71	1	0	3.025959	-6.276430	0.894766
72	8	0	-2.773644	3.271623	3.238260
73	1	0	-2.223240	3.891706	3.731200
74	1	0	-3.377344	3.820690	2.723947

SCF Done: E(RB3LYP) = -1833.45698641 A.U. after 1 cycles

	1	2	3
	A	A	A
Frequencies --	-525.1347	10.6385	12.2839

Zero-point correction= 0.603894 (a.u.)
Thermal correction to Energy= 0.671300
Thermal correction to Enthalpy= 0.672244
Thermal correction to Gibbs Free Energy= 0.482317
Sum of electronic and zero-point Energies= -1832.853092
Sum of electronic and thermal Energies= -1832.785687
Sum of electronic and thermal Enthalpies= -1832.784743
Sum of electronic and thermal Free Energies= -1832.974669

E (Thermal) CV S

	KCal/Mol	Cal/Mol-Kelvin	Cal/Mol-Kelvin
Total	421.247	211.794	399.734

[5] The TS geometry of 16B(Me) in bold orange color in Figure S6 along with that of the preceding species, 2-methylpropanonitrile and $(H_2O)_{11}$.

==2-methylpropanonitrile and (H2O)11==

str06djj.rev.high.log

Standard orientation:

Center Number	Atomic Number	Atomic Type	Coordinates (Angstroms) X	Y	Z
1	6	0	2.971292	-3.443066	-0.542530
2	6	0	4.282903	-4.073708	-0.033771
3	6	0	2.559118	-3.968835	-1.931977
4	1	0	2.430756	-5.051157	-1.875687
5	1	0	1.618065	-3.525884	-2.261407
6	1	0	3.329204	-3.749541	-2.674420
7	1	0	2.171820	-3.669593	0.171155
8	6	0	3.081702	-1.982217	-0.561872
9	7	0	3.174526	-0.832678	-0.584043
10	1	0	4.544063	-3.702503	0.958412
11	1	0	4.154185	-5.155885	0.024899
12	1	0	5.107425	-3.859604	-0.716919
13	8	0	-2.266268	-1.187024	-1.090880
14	1	0	-2.932979	-1.357487	-0.398898
15	1	0	-1.907701	-0.297140	-0.903063
16	8	0	-1.223803	1.371574	-0.577861
17	1	0	-0.282242	1.436983	-0.316288
18	1	0	-1.370653	2.059291	-1.255540
19	8	0	1.448445	1.553280	0.193796
20	1	0	2.023712	0.805600	-0.027200
21	1	0	1.624468	1.764343	1.134587
22	8	0	1.932759	2.190293	2.855126

23	1	0	2.199534	3.111672	3.051095
24	1	0	1.235093	1.979043	3.485015
25	8	0	-1.677216	3.350860	-2.530032
26	1	0	-1.908927	3.045777	-3.413885
27	1	0	-2.314164	4.063209	-2.319723
28	8	0	-4.205405	-1.719649	0.895758
29	1	0	-5.130678	-1.843283	0.616217
30	1	0	-4.018216	-2.425033	1.541517
31	8	0	-3.526488	-3.719657	2.793649
32	1	0	-3.927511	-3.669526	3.669326
33	1	0	-3.601383	-4.645740	2.534953
34	8	0	-6.901823	-2.052544	0.061130
35	1	0	-7.522814	-1.349995	0.287029
36	1	0	-7.049948	-2.227582	-0.875744
37	8	0	5.155535	1.282203	-1.438867
38	1	0	4.482551	0.643622	-1.155334
39	1	0	4.707943	2.134904	-1.418123
40	8	0	2.721238	4.812611	3.390819
41	1	0	3.656937	4.958214	3.574654
42	1	0	2.484577	5.479276	2.735049
43	8	0	-3.474398	5.399862	-1.902259
44	1	0	-4.162166	5.193723	-1.258074
45	1	0	-3.093323	6.235531	-1.607434

SCF Done: E(RB3LYP) = -1052.66778960 A.U. after 1 cycles

Zero-point correction= 0.366581 (a.u.)
Thermal correction to Energy= 0.410286
Thermal correction to Enthalpy= 0.411230
Thermal correction to Gibbs Free Energy= 0.266538
Sum of electronic and zero-point Energies= -1052.301209
Sum of electronic and thermal Energies= -1052.257503
Sum of electronic and thermal Enthalpies= -1052.256559
Sum of electronic and thermal Free Energies= -1052.401252

E (Thermal) CV S

	KCal/Mol	Cal/Mol-Kelvin	Cal/Mol-Kelvin
Total	257.459	131.241	304.531

==16B(Me), TS==

str06djj.high.log

Standard orientation:

Center Number	Atomic Number	Atomic Type	Coordinates (Angstroms) X	Y	Z
1	6	0	-1.389000	-2.373754	-0.458802
2	6	0	-0.877141	-3.774972	-0.111452
3	6	0	-2.149364	-2.375660	-1.799862
4	1	0	-2.986812	-3.077702	-1.759297
5	1	0	-2.545222	-1.387077	-2.040292
6	1	0	-1.487480	-2.685303	-2.614376
7	1	0	-2.092612	-2.063973	0.321639
8	6	0	-0.252312	-1.361273	-0.490167
9	7	0	0.957117	-1.453751	-0.621439
10	1	0	-0.361897	-3.782787	0.852032
11	1	0	-1.715724	-4.475042	-0.056255
12	1	0	-0.178119	-4.137098	-0.868419
13	8	0	-0.909287	0.050072	-0.394002
14	1	0	-1.775903	0.067202	0.082383
15	1	0	-0.148018	1.088706	-0.161677
16	8	0	0.558870	1.940984	0.028214
17	1	0	1.495545	1.532707	-0.153105
18	1	0	0.388056	2.706880	-0.587307
19	8	0	2.627020	0.614067	-0.483373
20	1	0	2.044669	-0.230420	-0.548119
21	1	0	3.284210	0.476004	0.228181
22	8	0	4.522534	0.237163	1.555104
23	1	0	5.467708	0.192277	1.305519
24	1	0	4.479105	0.836365	2.308175
25	8	0	0.139135	4.025763	-1.559408
26	1	0	-0.237071	3.860354	-2.431130

27	1	0	-0.365867	4.776916	-1.179607
28	8	0	-3.277612	0.354169	0.925140
29	1	0	-4.100517	0.042692	0.503081
30	1	0	-3.323287	0.080650	1.860846
31	8	0	-3.273437	-0.362785	3.652851
32	1	0	-3.971314	-0.022770	4.225218
33	1	0	-3.192716	-1.296623	3.880187
34	8	0	-5.642352	-0.547541	-0.327971
35	1	0	-6.315437	0.108101	-0.545549
36	1	0	-5.529793	-1.069725	-1.131108
37	8	0	2.608487	-3.614102	-1.083492
38	1	0	1.966668	-2.867248	-0.904194
39	1	0	3.449013	-3.179679	-1.260933
40	8	0	7.216818	0.080740	0.830329
41	1	0	7.630419	-0.789215	0.880380
42	1	0	7.461542	0.428287	-0.035586
43	8	0	-1.259086	6.148397	-0.478131
44	1	0	-1.760779	5.985600	0.329568
45	1	0	-0.760011	6.956705	-0.310573

SCF Done: E(RB3LYP) = -1052.61200073 A.U. after 1 cycles

NFock= 1 Conv=0.16D-08 -V/T= 2.0047

	1	2	3
	A	A	A
Frequencies --	-476.5053	6.3112	8.6964

Zero-point correction= 0.365861 (a.u.)

Thermal correction to Energy= 0.404522

Thermal correction to Enthalpy= 0.405466

Thermal correction to Gibbs Free Energy= 0.281926

Sum of electronic and zero-point Energies= -1052.246139

Sum of electronic and thermal Energies= -1052.207479

Sum of electronic and thermal Enthalpies= -1052.206535

Sum of electronic and thermal Free Energies= -1052.330075

	E (Thermal)	CV	S
	KCal/Mol	Cal/Mol-Kelvin	Cal/Mol-Kelvin
Total	253.841	120.394	260.013

[6] Two TS geometries along with the geometry of the precursor in the extended model with stoichiometry C3H49N2O21(1+) in Figure S7

==precursor==

str06dcc.extrev.log

Standard orientation:

Center Number	Atomic Number	Atomic Type	Coordinates (Angstroms) X	Y	Z
1	6	0	-0.163037	-0.722409	4.912467
2	7	0	-0.641521	-1.937507	5.570349
3	6	0	-0.835251	0.524452	5.501320
4	1	0	-0.614354	0.567764	6.568271
5	1	0	-0.462358	1.431906	5.024057
6	1	0	-1.918236	0.478496	5.367167
7	1	0	0.916330	-0.655097	5.075731
8	6	0	-0.349758	-0.757172	3.439896
9	7	0	-0.508858	-0.818555	2.299913
10	1	0	-0.156118	-2.760677	5.227879
11	1	0	-0.712540	-1.114465	0.434546
12	1	0	-1.634660	-2.074452	5.405656
13	8	0	-0.813787	-1.276721	-0.525498
14	1	0	-0.844250	-2.266098	-0.643008
15	1	0	-1.945340	-0.418691	-1.147694
16	8	0	-2.634471	0.196050	-1.578784
17	1	0	-3.509970	0.221157	-1.025590
18	1	0	-2.234551	1.149624	-1.707130
19	8	0	-4.780554	0.198809	-0.259561
20	1	0	-4.844879	0.820887	0.494165
21	1	0	-5.607218	0.285015	-0.788320

22	8	0	-1.630227	2.465666	-1.953508
23	1	0	-0.911027	2.748719	-1.334434
24	1	0	-2.240235	3.221670	-2.073144
25	8	0	4.013900	0.074150	1.062059
26	1	0	3.720625	-0.793257	0.718996
27	1	0	4.972148	0.092004	0.873891
28	8	0	6.717761	-0.019564	0.182767
29	1	0	6.911163	0.785633	-0.355483
30	1	0	7.434753	-0.099109	0.822499
31	8	0	3.295251	-2.280292	-0.411244
32	1	0	2.740744	-1.747142	-1.029306
33	1	0	4.184560	-2.358920	-0.818281
34	8	0	1.629935	-0.641203	-1.861446
35	1	0	0.766869	-0.782224	-1.426403
36	1	0	1.932557	0.242503	-1.570292
37	8	0	5.909889	-2.215348	-1.352319
38	1	0	6.318150	-1.497317	-0.825436
39	1	0	6.508200	-2.967683	-1.288724
40	8	0	2.710468	1.713504	-0.735370
41	1	0	3.208431	1.221055	-0.040535
42	1	0	3.373686	2.188333	-1.269014
43	8	0	-7.073230	0.432911	-1.719062
44	1	0	-6.984789	0.697127	-2.641335
45	1	0	-7.700303	-0.320102	-1.712604
46	8	0	-0.753234	-3.937126	-0.774483
47	1	0	-0.942966	-4.313665	-1.654837
48	1	0	0.152658	-4.227093	-0.532215
49	8	0	-1.333903	-5.034144	-3.308773
50	1	0	-2.156421	-5.529726	-3.399006
51	1	0	-1.310708	-4.442015	-4.069802
52	8	0	-8.859221	-1.694914	-1.677023
53	1	0	-8.515009	-2.558688	-1.420272
54	1	0	-9.667479	-1.579940	-1.163280
55	8	0	-3.366119	4.614360	-2.302090
56	1	0	-3.378430	5.045853	-3.164717
57	1	0	-4.293041	4.487747	-2.067239

58	8	0	7.097839	2.221190	-1.309666
59	1	0	7.750078	2.197113	-2.018712
60	1	0	6.272618	2.552843	-1.714696
61	8	0	4.624605	3.124579	-2.354926
62	1	0	4.502287	2.914661	-3.290098
63	1	0	4.513070	4.082399	-2.293322
64	8	0	0.396432	3.216311	-0.350041
65	1	0	0.270513	3.459767	0.585854
66	1	0	1.224680	2.696660	-0.404284
67	8	0	-4.962507	1.948909	1.891059
68	1	0	-4.961662	1.565856	2.776348
69	1	0	-4.337785	2.682789	1.930129
70	1	0	2.418579	-3.788746	-0.118378
71	8	0	1.825506	-4.557497	0.050116
72	1	0	2.276997	-5.325259	-0.316760
73	8	0	0.029550	3.980181	2.344832
74	1	0	0.045178	4.926037	2.532868
75	1	0	-0.740679	3.641464	2.816062

SCF Done: E(RB3LYP) = -1833.93877635 A.U. after 1 cycles

Zero-point correction= 0.620453 (a.u.)

Thermal correction to Energy= 0.688505

Thermal correction to Enthalpy= 0.689450

Thermal correction to Gibbs Free Energy= 0.491525

Sum of electronic and zero-point Energies= -1833.318323

Sum of electronic and thermal Energies= -1833.250271

Sum of electronic and thermal Enthalpies= -1833.249327

Sum of electronic and thermal Free Energies= -1833.447252

	E (Thermal)	CV	S
	KCal/Mol	Cal/Mol-Kelvin	Cal/Mol-Kelvin
Total	432.044	214.868	416.568

==16Bext TS in the left og Figure S7==

str06dj.ext.log

Standard orientation:

Center Number	Atomic Number	Atomic Type	Coordinates (Angstroms) X	Y	Z
1	6	0	-1.632538	-0.817837	0.179349
2	7	0	-2.399508	0.378559	-0.298174
3	6	0	-2.428003	-1.570381	1.247853
4	1	0	-3.391658	-1.897281	0.851696
5	1	0	-1.870218	-2.452120	1.566104
6	1	0	-2.599135	-0.936608	2.120744
7	1	0	-1.503890	-1.454362	-0.696175
8	6	0	-0.274380	-0.356351	0.704275
9	7	0	0.066186	0.644849	1.304554
10	1	0	-1.885440	0.841752	-1.077987
11	1	0	-3.328736	0.080560	-0.669140
12	1	0	-2.521316	1.074578	0.463985
13	8	0	0.704590	-1.520871	0.416470
14	1	0	0.571549	-1.901114	-0.495197
15	1	0	1.913716	-1.242719	0.585825
16	8	0	3.036635	-0.943326	0.706789
17	1	0	3.028610	-0.050447	1.209959
18	1	0	3.583744	-1.635159	1.202073
19	8	0	2.641659	1.337591	1.779848
20	1	0	1.652922	1.169898	1.658702
21	1	0	2.894066	1.999769	1.104565
22	8	0	-1.036507	1.460057	-2.525268
23	1	0	-0.865257	2.416540	-2.574869
24	1	0	-0.215542	1.013794	-2.814120
25	8	0	-4.753144	-0.503741	-1.531243
26	1	0	-4.535289	-1.140912	-2.241751
27	1	0	-5.534366	-0.856281	-1.065450
28	8	0	3.429915	2.949000	-0.406484
29	1	0	2.799766	3.653733	-0.673123
30	1	0	4.309376	3.372081	-0.336439
31	8	0	4.477935	-2.698791	1.957749

32	1	0	4.069817	-3.137766	2.730947
33	1	0	4.878514	-3.400663	1.406405
34	8	0	0.524820	-2.402898	-2.107920
35	1	0	-0.227099	-2.876517	-2.506774
36	1	0	0.717028	-1.625820	-2.671258
37	8	0	1.247772	0.015372	-3.317264
38	1	0	2.070823	0.252255	-2.815161
39	1	0	1.475907	0.064092	-4.252836
40	8	0	-1.702295	-3.885706	-3.166384
41	1	0	-1.456646	-4.347545	-3.979000
42	1	0	-1.920861	-4.586477	-2.537711
43	8	0	-2.100494	2.323427	1.759182
44	1	0	-1.248396	1.811869	1.748586
45	1	0	-2.445904	2.292898	2.671824
46	8	0	-0.666747	4.325104	-2.636846
47	1	0	-0.606999	4.651011	-3.544443
48	1	0	-1.452745	4.749492	-2.268385
49	8	0	-4.038448	-2.276846	-3.566611
50	1	0	-3.995026	-1.912210	-4.457495
51	1	0	-3.226306	-2.806805	-3.457543
52	8	0	-7.025744	-1.507088	-0.183463
53	1	0	-6.971287	-1.640055	0.770224
54	1	0	-7.866520	-1.057326	-0.328528
55	8	0	1.668709	4.948749	-1.138780
56	1	0	0.858991	4.738612	-1.642753
57	1	0	1.386415	5.528999	-0.423019
58	8	0	5.959297	4.133006	-0.194614
59	1	0	6.392547	4.099234	0.666504
60	1	0	6.632539	3.858293	-0.828369
61	8	0	5.643869	-4.686842	0.381887
62	1	0	5.228042	-4.891046	-0.464168
63	1	0	6.585311	-4.603943	0.188899
64	8	0	3.291565	-3.922751	4.171523
65	1	0	3.775653	-3.894364	5.005327
66	1	0	2.393268	-3.647940	4.390595
67	8	0	3.436466	0.555784	-1.832066

68	1	0	3.440222	1.436485	-1.396506
69	1	0	3.455870	-0.081065	-1.099796
70	8	0	-3.112200	2.287521	4.401085
71	1	0	-3.020118	3.109323	4.895203
72	1	0	-4.032104	1.990638	4.551592
73	8	0	-5.739242	1.420267	4.819409
74	1	0	-6.293450	1.288279	4.041124
75	1	0	-5.864892	0.629759	5.357589

SCF Done: E(RB3LYP) = -1833.89612398 A.U. after 1 cycles

	1	2	3
	A	A	A
Frequencies --	-661.1972	7.2091	9.2884

Zero-point correction= 0.620068 (a.u.)
Thermal correction to Energy= 0.686168
Thermal correction to Enthalpy= 0.687112
Thermal correction to Gibbs Free Energy= 0.499692
Sum of electronic and zero-point Energies= -1833.276056
Sum of electronic and thermal Energies= -1833.209956
Sum of electronic and thermal Enthalpies= -1833.209012
Sum of electronic and thermal Free Energies= -1833.396432

	E (Thermal)	CV	S
	KCal/Mol	Cal/Mol-Kelvin	Cal/Mol-Kelvin
Total	430.577	211.278	394.458

==2ext TS in the right of Figure S7==
str06dc.ext.log
Standard orientation:

Center Number	Atomic Number	Atomic Type	Coordinates (Angstroms) X	Y	Z
1	6	0	0.654897	-0.915560	-2.493549

2	7	0	1.581379	-1.956515	-2.886112
3	6	0	0.743507	0.301327	-3.425878
4	1	0	0.495302	-0.022189	-4.437230
5	1	0	0.051872	1.090160	-3.129489
6	1	0	1.757181	0.707386	-3.426579
7	1	0	-0.358612	-1.321107	-2.522824
8	6	0	0.886388	-0.489849	-1.063884
9	7	0	1.689526	-0.527622	-0.186877
10	1	0	1.517318	-2.742151	-2.242690
11	1	0	1.727335	-0.177237	0.811349
12	1	0	2.542817	-1.617053	-2.921376
13	8	0	2.046438	0.252529	2.375708
14	1	0	2.056971	-0.589495	2.887407
15	1	0	2.978686	0.584737	2.321804
16	8	0	4.582916	1.079012	1.891307
17	1	0	4.793751	0.516087	1.123137
18	1	0	4.577551	2.005586	1.568168
19	8	0	4.762361	-0.866401	-0.222239
20	1	0	5.266680	-1.642801	0.104727
21	1	0	3.825923	-1.109060	-0.166472
22	8	0	4.420434	3.729122	1.125041
23	1	0	3.589229	3.997141	0.673925
24	1	0	5.135245	4.159548	0.644011
25	8	0	-0.673051	0.409216	-0.608438
26	1	0	-0.955975	0.050124	0.293548
27	1	0	-1.481305	0.284747	-1.194378
28	8	0	-2.992721	0.128978	-1.836249
29	1	0	-3.305189	0.926458	-2.316746
30	1	0	-3.175081	-0.642394	-2.415637
31	8	0	-1.553434	-0.423133	1.721485
32	1	0	-1.247549	0.313618	2.305468
33	1	0	-2.528374	-0.329589	1.564890
34	8	0	-0.293772	1.676739	2.936955
35	1	0	0.598317	1.280405	2.862088
36	1	0	-0.328740	2.320579	2.203600
37	8	0	-4.066954	-0.124355	0.830677

38	1	0	-3.880404	-0.075468	-0.124759
39	1	0	-4.782710	-0.783431	0.952154
40	8	0	-0.309588	3.137650	0.468763
41	1	0	-0.436965	2.337537	-0.068731
42	1	0	-1.090015	3.708830	0.301960
43	8	0	-6.111958	-1.954996	1.203809
44	1	0	-5.898493	-2.778046	1.656792
45	1	0	-6.930369	-1.622077	1.626012
46	8	0	1.832790	-2.224082	3.528341
47	1	0	1.671529	-2.328905	4.472546
48	1	0	1.030028	-2.562205	3.077396
49	8	0	4.576563	-0.949422	-3.037742
50	1	0	4.821158	-0.123420	-3.468672
51	1	0	4.789567	-0.825283	-2.092115
52	8	0	0.460557	-3.771907	-0.576708
53	1	0	0.158795	-3.500483	0.311238
54	1	0	0.002823	-4.600305	-0.755618
55	8	0	-3.569304	-2.048281	-3.442875
56	1	0	-2.880984	-2.426391	-4.003020
57	1	0	-3.995296	-2.801963	-3.017293
58	8	0	-3.862959	2.385386	-3.176560
59	1	0	-4.634753	2.843330	-2.822560
60	1	0	-3.212011	3.076869	-3.346045
61	8	0	-2.498787	4.777123	0.000952
62	1	0	-3.205033	4.763268	0.657990
63	1	0	-2.318329	5.711172	-0.159256
64	8	0	2.053458	4.497266	-0.063204
65	1	0	2.066559	4.674263	-1.010060
66	1	0	1.221106	4.003390	0.105225
67	8	0	6.224203	-3.036231	0.694634
68	1	0	6.876695	-3.415166	0.093621
69	1	0	6.683935	-2.929633	1.535939
70	1	0	-0.896512	-2.046264	1.960130
71	8	0	-0.418110	-2.906050	2.024790
72	1	0	-1.039642	-3.526689	2.424057
73	8	0	-8.444236	-0.989319	2.385302

74	1	0	-8.370678	-0.184084	2.911300
75	1	0	-9.207230	-0.849641	1.811880

SCF Done: E(RB3LYP) = -1833.89231314 A.U. after 1 cycles

	1	2	3
	A	A	A
Frequencies --	-270.8036	8.0715	9.7874

Zero-point correction= 0.621689 (a.u.)

Thermal correction to Energy= 0.688424

Thermal correction to Enthalpy= 0.689368

Thermal correction to Gibbs Free Energy= 0.503274

Sum of electronic and zero-point Energies= -1833.270624

Sum of electronic and thermal Energies= -1833.203890

Sum of electronic and thermal Enthalpies= -1833.202945

Sum of electronic and thermal Free Energies= -1833.389039

	E (Thermal)	CV	S
	KCal/Mol	Cal/Mol-Kelvin	Cal/Mol-Kelvin
Total	431.992	213.335	391.667
